# γ-Butyrolactone-induced coelimycin synthesis inhibits AtrA-dependent actinorhodin overproduction in *Streptomyces coelicolor* A3(2)

**DOI:** 10.1128/mbio.00630-26

**Published:** 2026-04-15

**Authors:** Bartosz Bednarz, Magdalena Kotowska, Mateusz Wenecki, Michał Tracz, Marta Derkacz, Adrianna Jastrzemska, Jarosław Ciekot, Lizaveta Karpovich, Krzysztof Pawlik

**Affiliations:** 1 Laboratory of Biological Chemistry, Faculty of Biotechnology, University of Wroclaw, Wroclaw, Poland; 2 Polish Academy of Sciences, Hirszfeld Institute of Immunology and Experimental Therapy, Wroclaw, Poland; 3 Laboratory of Mass Spectrometry, Faculty of Biotechnology, University of Wroclaw, Wroclaw, Poland; University of Strathclyde, Glasgow, United Kingdom

**Keywords:** *Streptomyces*, secondary metabolism, antibiotic biosynthesis regulation, γ-butyrolactones, coelimycin, actinorhodin, TetR family, quorum sensing

## Abstract

*Streptomyces coelicolor* A3(2) is a highly potent model antibiotic producer, with a chromosome harboring at least 20 secondary metabolite biosynthetic gene clusters (BGCs). Precise control of these multiple pathways is provided by a complex network of regulatory proteins and small signaling molecules. In this work, we show that coelimycin (CPK) and actinorhodin (ACT) production are exclusive processes. Induction of CPK synthesis by γ-butyrolactones inhibited ACT production when we simultaneously overexpressed the ACT upper activator protein AtrA; yet, this effect was not observed under overexpression of ActII-orf4—the direct activator of the ACT BGC and the direct AtrA target. We conclude that CPK intermediates inhibit the cascade of ACT induction at the level of AtrA pleiotropic regulator activity. Our proteomic data reveal that, besides being an ACT inducer, AtrA is a high-level regulator that activates arsenopolyketide synthesis but inhibits the production of undecylprodigiosin and calcium-dependent antibiotic. AtrA also downregulates primary metabolism of carbohydrates, lipids, and amino acids, along with the tricarboxylic acid cycle.

**IMPORTANCE:**

Bacteria from the genus *Streptomyces* possess one of the most complex gene regulation machineries in the bacterial kingdom, allowing them to coordinate production of multiple compounds at discrete timing. Navigating through these regulatory networks to unlock the biosynthetic potential is challenging. Expression of silent biosynthetic gene clusters (BGCs) often occurs only under strict culture conditions and, as in the case of coelimycin, can be coordinated via quorum sensing. Here, we have investigated the interplay between coelimycin and actinorhodin production in the model organism *Streptomyces coelicolor* A3(2). We show that even the products of BGCs can have a regulatory function. The richness of potentially competing biosynthetic pathways may hinder high-yield biosynthesis of the desired product under fermentation conditions. Our results show the importance of designing host organisms with lowered background regulation by γ-butyrolactones for the desired biosynthetic pathway activation.

## INTRODUCTION

*Streptomyces* are renowned for producing two-thirds of currently used antibiotics as well as anthelmintic, anticancer, and immunosuppressant secondary metabolites. They are also producers of biocontrol and plant growth-promoting agents and industrially relevant enzymes (1, 2). The study of antibiotic production in *Streptomyces* has contributed greatly to the understanding of the field of bacterial communication.

The synchronized behavior of bacteria is regulated by cell-to-cell signaling systems called quorum sensing, consisting of membrane-diffusible molecules and their receptor proteins, which usually act as response regulators. *Streptomyces* use γ-butyrolactones (GBLs) and related classes of butenolides and furans as signal molecules (3–6). GBL receptors generally belong to the TetR family regulators, typically repressors, which dissociate from DNA upon GBL binding.

*Streptomyces coelicolor* A3(2) produces four compounds with antimicrobial activities: coelimycin A (CPK A), calcium-dependent antibiotic (CDA), undecylprodigiosin (RED), and actinorhodin (ACT) (7). The production of all secondary metabolites must be tightly controlled, taking into account the timing (cell density and differentiation state), nutrient availability, and competition or symbiosis with other organisms. The regulation is exerted in a cascade manner through pleiotropic and pathway-specific regulators (8). Genes of the most immediate regulators, *Streptomyces* antibiotic regulatory proteins (SARPs), are localized within respective biosynthetic gene clusters (BGCs) (8). Their transcription is controlled by upper-level regulators. One such protein, the TetR-like pleiotropic regulator ScbR (9), the direct repressor of the coelimycin synthesis activator gene *cpkO*, is a well-studied GBL receptor (10, 11).

When the cell density and GBL level reach a threshold, ScbR dissociates from the *cpkO* promoter upon GBL binding, which results in CPK production activation (12–14). We have recently reviewed the precise mechanism of this process (15). SlbR, which does not belong to the TetR family, was found to dissociate from *scbR/scbA* promoter in the presence of GBL SCB1, although no specific regulatory mechanism was assigned (16–18). However, our current results put into question the GBL binding by SlbR.

This work concentrates on the role of the pleiotropic regulator AtrA in the context of the GBL-dependent quorum sensing system of *S. coelicolor* A3(2).

AtrA directly activates the transcription of *actII-orf4* gene encoding the SARP activator of ACT biosynthesis (19). Together with DasR, it takes part in linking carbon utilization with secondary metabolism. Under starvation conditions, AtrA was found to directly activate transcription of the *nagE2* gene (20). NagE2 permease is the transporter of N-acetylglucosamine (GlcNAc)—a molecule indicating cell wall peptidoglycan autolysis, which is crucial for the development and antibiotic production (21, 22). Another direct target of activation by AtrA is SsgR (17), a regulator participating in sporulation (23, 24). Further evidence of AtrA involvement in the regulation of carbon utilization, cell wall biogenesis, and development comes from ChIP-seq and RNA-seq experiments (8, 25). It was also found that AtrA could bind to the intergenic region between its own gene (*SCO4118*) and *SCO4119* and activate transcription of *SCO4119,* encoding a putative NADH dehydrogenase (26). However, the possibility of AtrA autoregulation was left unverified.

*S. coelicolor* A3(2) AtrA can bind to the promoter sequence of the *Streptomyces griseus strR* gene encoding the final regulator of streptomycin production (19). Later, it was shown that it influenced streptomycin production in this strain, but surprisingly, the effect was inhibitory (27).

The AtrA homolog from *Streptomyces avermitilis*, AveI, directly regulates 35 genes. It positively regulates morphological differentiation and melanin synthesis but inhibits oligomycin and avermectin production. AveI can replace AtrA in *S. coelicolor* A3(2) and activate actinorhodin synthesis, while AtrA functions in *S. avermitilis* in the same way as AveI in downregulating avermectin production (28).

AtrA homologs from *Streptomyces pristinaespiralis*, *Streptomyces lincolnensis*, *Streptomyces roseosporus,* and *Streptomyces globisporus* positively influence synthesis of pristinamycin (29), lincomycin (30), daptomycin (31), and lidamycin (32), respectively.

An important revelation was that *S. globisporus* AtrA was a part of a negative feedback loop in which the protein directly activated the transcription of a lidamycin BGC activator, and this interaction was in turn inhibited by AtrA binding to heptaene, a lidamycin production intermediate. In the same paper, the protein was shown to bind *S. coelicolor* A3(2) actinorhodin, which caused its dissociation from the DNA. However, it was not specified which form of actinorhodin could be the actual ligand (32). Later work of Al-Tarawni (25) led to the conclusion that γ-ACT was a nonspecific inhibitor of multiple transcription factors, including AtrA from *S. coelicolor* A3(2).

In his effort to assess the ability of ACT to interact with *S. coelicolor* AtrA, Hassan (33) unexpectedly observed that a crude extract from the M1146 strain (Δ*act*, Δ*red*, Δ*cpk*, and Δ*cda*) (34) inhibited the DNA-binding activity of AtrA. However, the active compound was not identified. The M1146 strain is a potent producer of GBLs due to the lack of *scbR2* gene and the inability of ScbR2 to repress *scbA* (the GBL synthase gene) (35).

In the present study, we investigated whether γ-butyrolactone(s) could be the mysterious signal molecule(s) interacting with AtrA and dissociating it from the DNA. Based on the results, we have rejected this initial hypothesis. Surprisingly, we have discovered that ACT synthesis is inhibited not directly by GBLs but by the coelimycin synthesis induced by GBLs. We have determined that CPK intermediates inhibit the cascade of ACT induction at the upper level of AtrA pleiotropic regulator activity. Moreover, we provide proteomic profiles of AtrA overexpression in a GBL-free background strain and an AtrA deletion strain.

## MATERIALS AND METHODS

### Bacterial strains and growth conditions

Bacterial strains used in this study are listed in Table S1. Detailed growth conditions are provided in the Supplemental material.

### *S. coelicolor* A3(2) *atrA* deletion and overexpression mutants

The construct creation history and oligonucleotides used in this study are listed in Tables S2 and S3, respectively. To obtain the *atrA* deletion strain ∆*atrA* (P332), the *atrA* gene was replaced with the *aac3(IV*) apramycin resistance cassette. The pIJ10257-atrA_OE_ construct containing the *atrA* gene under the control of the ermEp* promoter was introduced into the wild-type (M145), ∆*scbA* (M751) (11), ∆*cpkC* (P100) (13), and ∆*cpkF* (P112) (36) strains to obtain the M145-*atrA*_OE_ (P330), ∆*scbA-atrA*_OE_ (P331), ∆*cpkC-atrA*_OE_ (P337), and ∆*cpkF-atrA*_OE_ (P340) strains, respectively. The empty vector pIJ10257 was introduced into the M145, ∆*scbA*, ∆*cpkC,* and ∆*cpkF* strains to obtain the control strains M145-Φ (P132), ∆*scbA*-Φ (P053), ∆*cpkC*-Φ (P339), and ∆*cpkF*-Φ (P341), respectively.

### Label-free, bottom-up shotgun proteomics

Three biological replicates of *S. coelicolor* A3(2) strains were cultivated on solid medium 79NG for 27 h (∆*scbA*-Φ and ∆*scbA-atrA*_OE_) or 50 h (M145 and ∆*atrA*). The growth conditions and the processing of biomass were performed as described in reference 14. An in-depth description of the shotgun proteomics is provided in the Supplemental material. In order to generate the final list of the most pronounced and confident abundance changes, we focused on proteins meeting all of the following criteria: (i) identified using at least one unique peptide, (ii) showing a statistically significant difference with q value ≤0.05, and (iii) displaying mean abundance ratios of at least 1.5 between the strains. data were visualized using the pheatmap library in R (37). The mass spectrometry proteomics data have been deposited to the ProteomeXchange Consortium via the PRIDE (38) partner repository with the data set identifier PXD066756 and 10.6019/PXD066756.

### Measurement of actinorhodin production

The actinorhodin assay was adapted from Kieser et al. (39). Whole agar medium (20 mL) from a petri plate, after cellophane disk removal, was mixed with 10 mL of a methanol-chloroform (vol. 1:1) mixture. The sample was extracted for 30 min on a roller at room temperature and centrifuged (10 min, 4,800 × *g*). One hundred microliters of the upper phase was mixed with 5 µL of 5 M KOH. The absorbance at 640 nm was measured with a ClarioStar Plus microplate reader (BMG Labtech).

### Detection of actinothodin pathway compounds by LC-MS/MS

Extracts from the solid medium with biomass were analyzed by LC-MS/MS in a manner similar to (40). A detailed description of the procedure is provided in the Supplemental material.

### RNA isolation, reverse transcription, and quantitative PCR

Detailed steps are provided in the Supplemental material. The assay was performed for three biological replicates with at least three technical replicates for each biological replicate. In the reactions with 0.5 μM final primer concentration and an annealing temperature of 60°C, we achieved *R*^2^ = 0.99 and efficiencies of 97.71% for *hrdB*, 101.56% for *atrA*, 93.69% for *actII-orf4,* and 91.69% for *cpkO*. The relative gene expression ratio to *hrdB* was calculated using the Pfaffl formula (41).

### Purification of recombinant proteins

Purification of His-tagged ScbR, ScbR2, and HypR proteins was performed as described previously (14, 42). Glutathione S-transferase (GST) was a kind gift from Jakub Muraszko. Detailed purification protocols for His-tagged AtrA and SlbR proteins are provided in the Supplemental material.

### γ-Butyrolactone extraction

GBL extracts were prepared from 450 mL of *S. coelicolor* A3(2) culture supernatant as described previously (43). Cultures grown in liquid Glu-MM medium were centrifuged at 24,000 × *g* for 10 min. The supernatants were mixed with three volumes of ethyl acetate (POCH) and shaken 10 times. The upper ethyl acetate phase was collected and evaporated under vacuum. The residue was dissolved in 500 µL of methanol and stored at −20°C. For the electrophoretic mobility shift assays (EMSAs), 500 µL of the extracts were once again evaporated in a SpeedVac and dissolved in 250 µL of methanol. For the GBL capture experiment, GBL-containing extract was evaporated in a SpeedVac and mixed with an equal volume of water. The water-soluble fraction was used for the experiment.

### GBL affinity capture

The binding of GBLs to recombinant receptor proteins was assessed by the affinity capture method adapted from reference 44. Equimolar quantities of ScbR, ScbR2, AtrA, SlbR, and GST proteins, corresponding to 1.5 mg of ScbR, were washed three times with 3 mL of water using Amicon Ultra-4 (3 kDa cut-off, Millipore) (centrifugation at 4°C, 4,800 × *g*). The concentrated protein sample (approx. 150 μL) was mixed with water (up to 710 μL), 40 μL of 10× LI-COR buffer (100 mM Tris pH 7.5, 500 mM KCl, 10 mM DTT), and 50 μL of GBLs solution (extract from the M1154 strain grown for 72 h). After 12 h of gentle agitation at room temperature, the samples were subjected to ultrafiltration on the same Amicon Ultra-4 filters by adding four 3 mL portions of water. The sample volume was adjusted to 800 μL with water, and 2 μL of 6 M HCl was added. The mixtures were denatured at 95°C for 3 min and ultrafiltered. The filtrate was collected, concentrated 40 times with a SpeedVac, and subjected to HPLC-MS for GBL detection. The control sample without protein was processed in the same way.

### HPLC-MS analysis of γ-butyrolactones

A detailed description of the HPLC-MS analysis is provided in the Supplemental material.

### Two-plasmid green fluorescence protein (GFP) reporter assay

GFP reporter assay of Wilbanks et al. (45) was modified by placing the gene coding the tested protein (potential GBL receptor) on a separate expression plasmid. Utilizing the pKNT25 vector scaffold and the promoter controlling *scbR* in pLW0003, plasmids pMK48-scbR, pMK48-scbR2, pMK48-atrA, pMK-48-scbR, and empty pMK48 were created as described in Table S2. GFP reporter plasmid pMK49-siteR was based on pLW0002 omitting the *scbR* gene and introducing site R flanked by EcoRI and BamHI sites between the promoter and the *GFP* gene. The presence of restriction sites facilitated replacement of site R with other binding sites by ligation of annealed and phosphorylated oligonucleotides to create respective derivatives of pMK49 (Tables S2 and S3). Sequences of site R and site A were as identified by Takano et al. (11); sequences of sites FP1 and FP2 were taken from Uguru et al. (19). *Escherichia coli* TOP10 cells were co-transformed with pMK48 and pMK49 derivatives as shown in Table S4. Biomass from 3 mL of overnight cultures in LB medium supplemented with chloramphenicol (35 μg/mL) and kanamycin (50 μg/mL) was washed twice with supplemented M9 medium (supM9) (45) and suspended in 1 mL of this medium. Cell suspension was diluted to OD_600_ = 0.05 and dispensed into a clear, flat-bottom 96-well microplate (200 μL/well). Additional compounds (extracts containing GBLs and controls) were added in a volume of 5 μL. The plate was sealed with Breathe-Easy sealing membrane (Diversified Biotech) and incubated at 37°C with shaking (180 rpm) for 2 h. The sealing membrane was removed, and OD_600_ was measured on ClarioStar microplate reader. The cultures were transferred to a black microplate for the measurement of GFP fluorescence intensity. The settings of the ClarioStar reader were as follows: excitation 485 nm, emission 535 nm, gain 1800, focal height 7.9 mm.

### EMSA and actinorhodin extraction for EMSA

For a detailed description of protein-DNA assays, please refer to the Supplemental material.

## RESULTS

### Abundant production of GBLs by the Δ*cpkO* and M1154 strains

Deletion of *cpkO*, the main activator of coelimycin BGC, leads to an over 20-fold increase in the abundance of the GBL synthase ScbA, resulting from the absence of the ScbR2 repressor, which normally shuts down its expression (14). Increased GBL production was observed in the antibiotic production superhost *S. coelicolor* M1152 (34), from which four BGCs were deleted, including the *cpk* cluster with the *scbR2* gene, while *scbA* (and the accessory gene *scbB*) were left behind (35). In the present study, we used both M1154 (very similar to M1152) (34) and Δ*cpkO* strains as sources of GBLs.

Extracts from the culture media of Δ*cpkO* and M1154 were analyzed by HPLC-MS, with Δ*scbA* strain as a negative control. The presence of all GBLs (SCB1–8) reported by Sidda et al. (35) was confirmed in extracts from 48 h and 72 h cultures of both strains (Fig. S2). Interestingly, three other putative GBLs, tentatively named SCB9, SCB10, and SCB11, were detected. The observed molecular mass of compound SCB9 was identical to that of SCB1, SCB2, and four others (Acl-2 series) reported by Gruzina et al. (46), while the mass of compounds SCB10 and SCB11 was the same as that of SCB3, SCB7, and two others (Acl-1 series) (46) (Table S5). The putative new GBLs may represent some of the Acl compounds.

### A simple bioassay based on CPK induction via external GBLs

**Fig 1:**
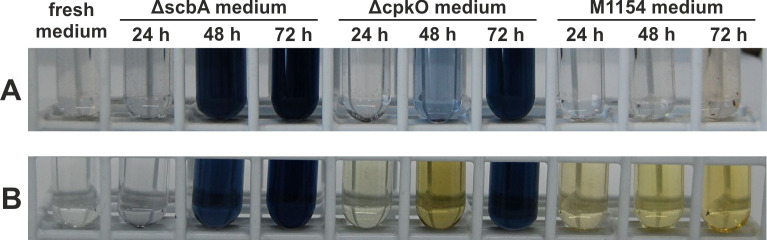
GBLs present in the Δ*cpkO* and M1154 culture media induce coelimycin (yellow pigment) production by the Δ*scbA* strain. (**A**) Supernatants of Δ*scbA,* Δ*cpkO,* and M1154 cultures in Glu-MM medium collected after the indicated times of growth. (**B**) The same supernatants after mixing with Δ*scbA* biomass and further incubation for 8 h.

Here, we developed a simple biological assay for detection of GBL production in liquid cultures. The Δ*scbA* strain, which is unable to synthesize GBLs, served as a reporter responding to external GBLs. A lack of GBLs provides complete repression of the *cpk* gene cluster by the ScbR protein in this strain. The addition of external GBLs leads to ScbR dissociation from DNA and allows coelimycin production. The use of Glu-MM medium ensures that all CPK precursors turn into the yellow glutamate adduct coelimycin P2 (47). Twenty-two-hour cultures of Δ*scbA* were centrifuged, and the biomass was mixed with spent culture media from Δ*cpkO* and M1154, which are not able to produce CPK. Medium from Δ*scbA* culture was used as a negative control. After several hours of additional cultivation, yellow pigment (yCPK) production was visible in the cultures in which GBLs were present (Fig. 1). As expected, media from both Δ*cpkO* and M1154 cultures derepressed CPK production in Δ*scbA*. An obvious limitation of the assay is the high concentration of actinorhodin in the tested culture media. However, the slightly blue color of the medium from the 48 h culture of Δ*cpkO* did not prevent the observation of CPK.

### γ-Butyrolactones are not AtrA ligands

To verify the possibility of GBL binding by AtrA, we applied three approaches: (i) EMSA, (ii) affinity capture method, and (iii) GFP reporter assay *in vivo*.

Initial EMSA results suggested that AtrA could be a γ-butyrolactone receptor that dissociates from its target sequence upon GBL binding, since the addition of GBL-rich extract prevented the binding of AtrA to DNA (Fig. S1A and B). However, this was a non-specific effect. Unexpectedly, addition of the GBL-containing extract prevented DNA binding by ScbR2 (Fig. S1C). This close homolog of ScbR is known as a “pseudo” GBL receptor. It shares some DNA targets with ScbR (9), but does not respond to GBL SCB1 (48). The same effect was observed for two proteins used as additional negative controls: HypR, a repressor belonging to the GntR family not associated with the GBL-dependent regulation (42) (Fig. S1D), and CemR protein from a taxonomically distant bacterium, *Helicobacter pylori* (49) (not shown).

**Fig 2:**
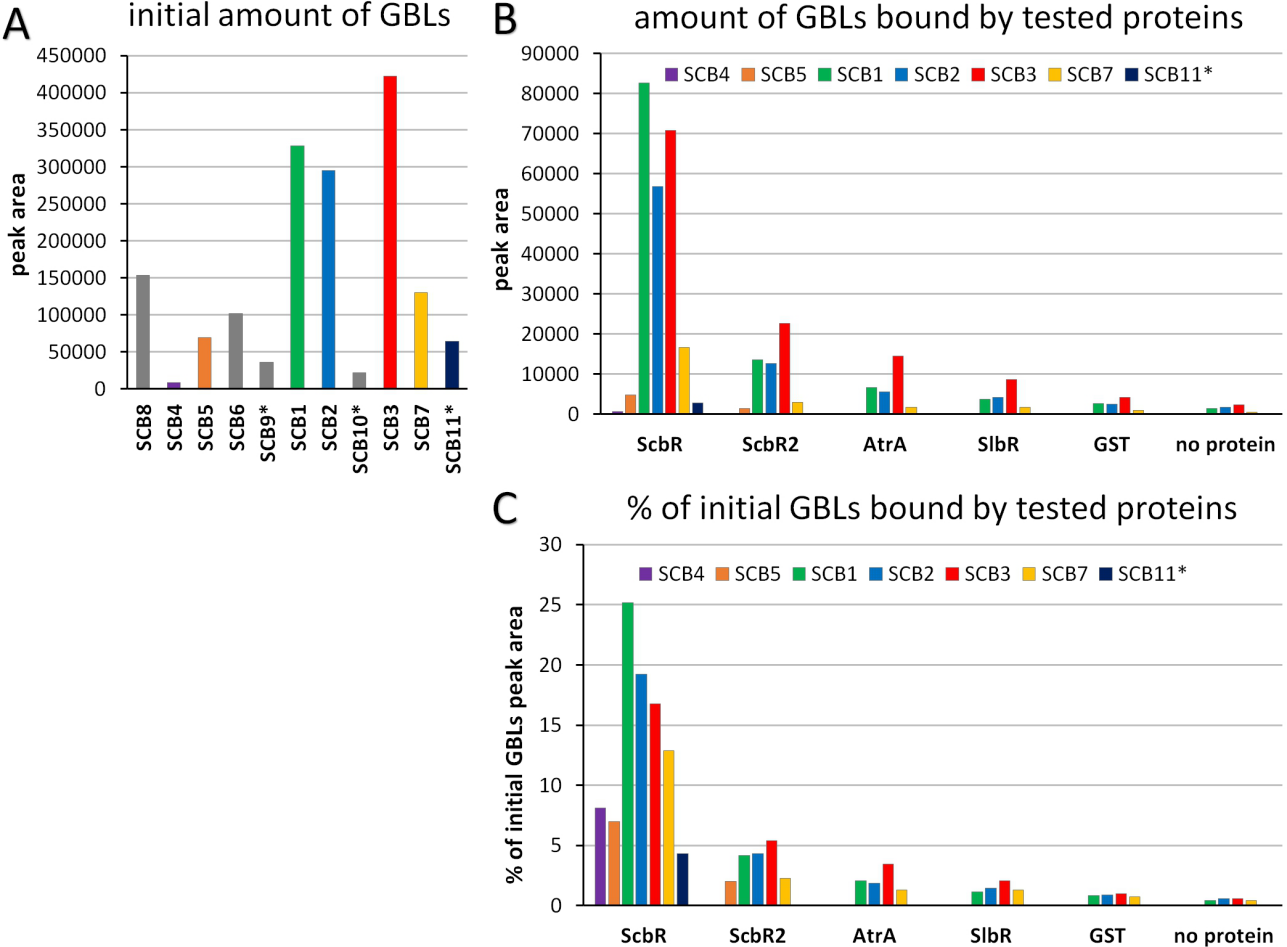
Affinity capture of GBLs by recombinant proteins. (**A**) GBLs detected by HPLC-MS in the extract from M1154 culture, quantified as peak areas from extracted ion chromatograms. The compounds were ordered according to their retention times. The putative GBLs reported for the first time in the current work are marked with asterisks. The gray columns denote GBLs not detected in the protein-bound samples. (**B**) GBLs captured by the recombinant proteins incubated with the GBL-containing extract characterized in panel A, quantified as peak areas from the extracted ion chromatograms. (**C**) GBLs captured by the recombinant proteins; peak areas from panel B are expressed as percentages of the respective peak areas from panel A.

To directly assess the ability of AtrA to bind GBLs, an affinity capture method adapted from (44) was applied. Extract from the M1154 strain grown for 72 h was used as a source of GBLs. It contained a mixture of eleven GBLs (Fig. 2A; Fig. S2). Two reported GBL receptors from *S. coelicolor* A3(2), ScbR and SlbR, were used as positive controls. ScbR2 and GST were chosen as negative controls. As expected, ScbR exhibited the greatest ability to bind different GBLs. (Fig. 2B and C; Fig. S3). Four compounds, namely, SCB6, 8, 9, and 10, were not detected in any of the protein-bound samples. The amounts of GBLs detected in the AtrA-bound sample exceeded those of SlbR; however, they were both lower than that of the negative control, ScbR2. This indicates that neither AtrA nor SlbR is indeed able to bind γ-butyrolactones.

**Fig 3:**
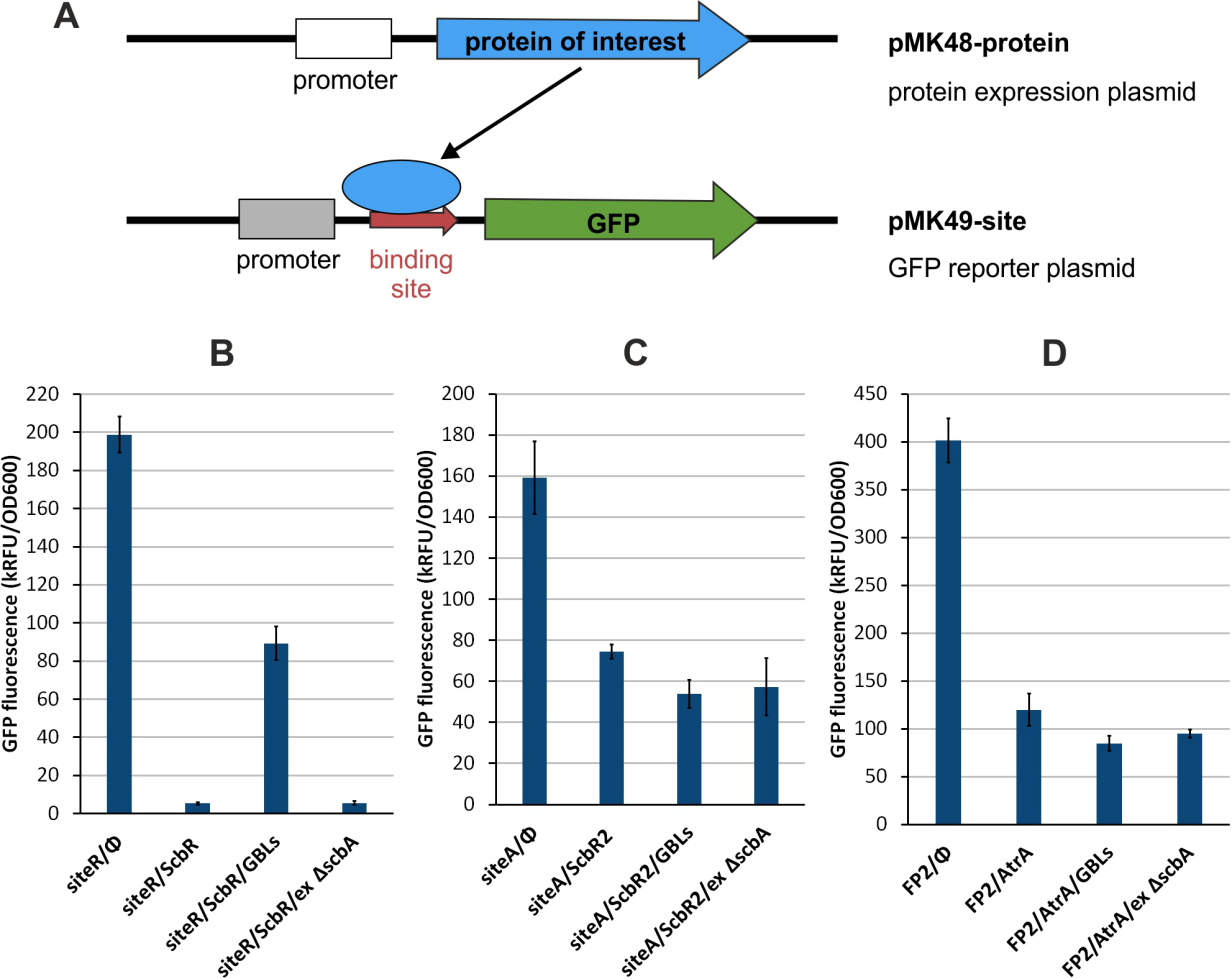
(**A**) Schematic representation of the two-plasmid GFP reporter assay principle. Protein (blue) expressed from one plasmid binds to its target site (red) located upstream of the GFP gene (green) on the other plasmid and inhibits GFP expression. Protein dissociation from DNA upon binding of GBLs leads to the recovery of GFP expression. (**B–D**) Results of the GFP reporter assay for the effect of GBLs on ScbR binding to site R, ScbR2 binding to site A, and AtrA binding to site FP2, respectively. Φ, empty plasmid pMK48; GBLs, extract from M1154 containing γ-butyrolactones; ex Δ*scbA*, control extract from the strain not producing GBLs.

To investigate the effect of γ-butyrolactones on AtrA binding to its target DNA sequences, a two-plasmid GFP reporter assay was applied. *E. coli* TOP10 was co-transformed with plasmid pMK48 derivatives for constitutive expression of a DNA-binding protein under study and pMK49 derivatives carrying oligonucleotide sequences recognized by the respective proteins inserted between the constitutive promoter and the GFP coding gene (Fig. 3A). In principle, protein binding to the inserted sequence interrupts transcription and results in a decrease of GFP fluorescence intensity in comparison to the strain with an empty pMK48 plasmid. If the addition of GBLs prevents protein binding to its target sequence, GFP fluorescence should be restored, as observed in the case of the GBL receptor ScbR binding to site R (Fig. 3B). In the case of the negative control—ScbR2 binding to site A—addition of GBLs did not restore GFP fluorescence (Fig. 3C). The same effect was observed for AtrA binding to site FP2, indicating that AtrA does not respond to GBLs *in vivo* (Fig. 3D).

We have also made an attempt to test SlbR in the same way, using site R and site A as its potential binding sites (18). It turned out that SlbR did not reduce GFP fluorescence in any case; therefore, it was not possible to assess the effect of GBLs on its ability to bind DNA (Fig. S4).

### AtrA-driven actinorhodin overproduction is inhibited by GBL-induced coelimycin synthesis

**Fig 4:**
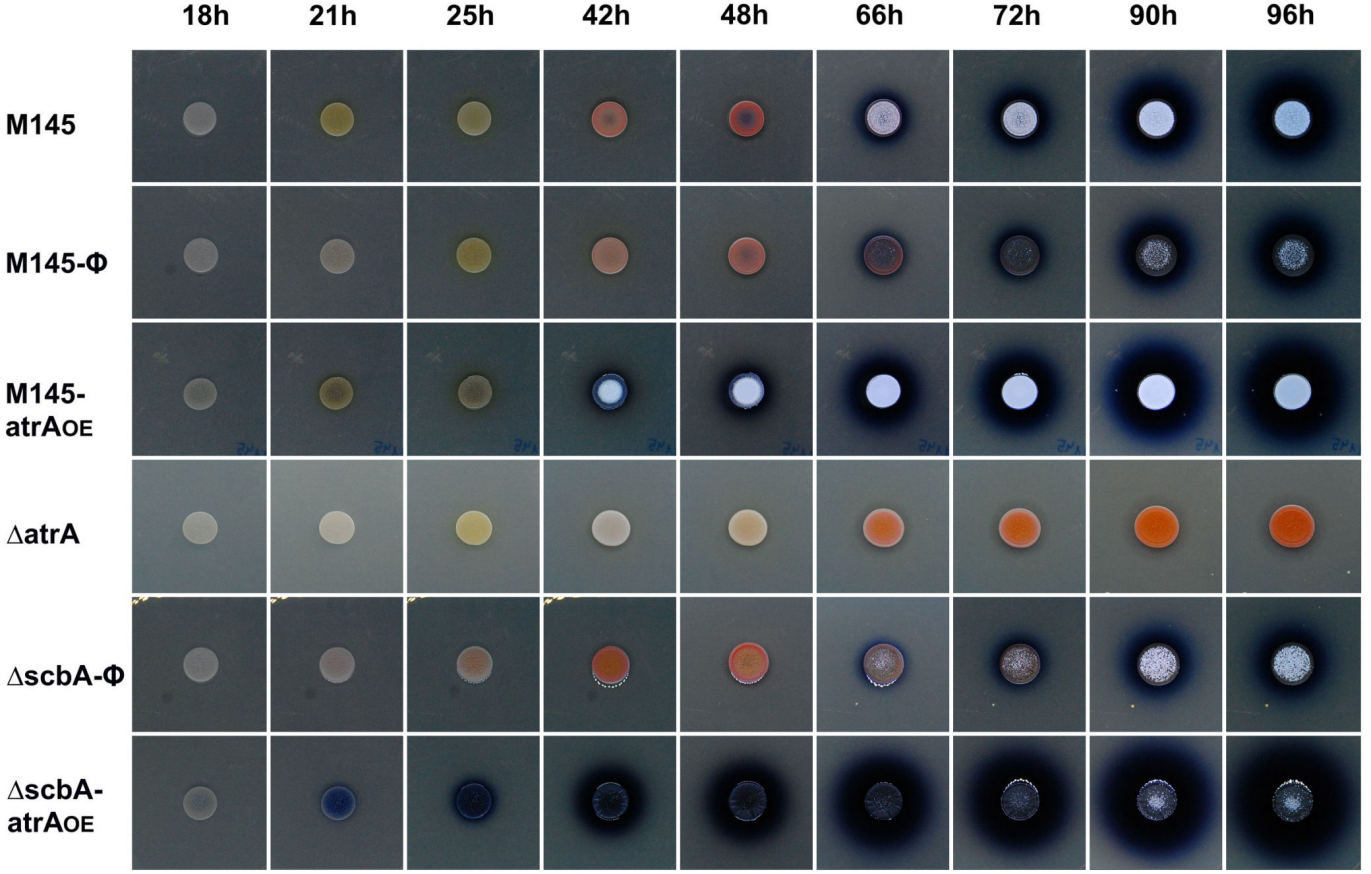
Phenotypes of the *S. coelicolor* A3(2) strains grown on solid medium 79NG as spots. Twenty microliters of spore suspension in water at OD_600_ = 0.30 was used.

**Fig 5:**
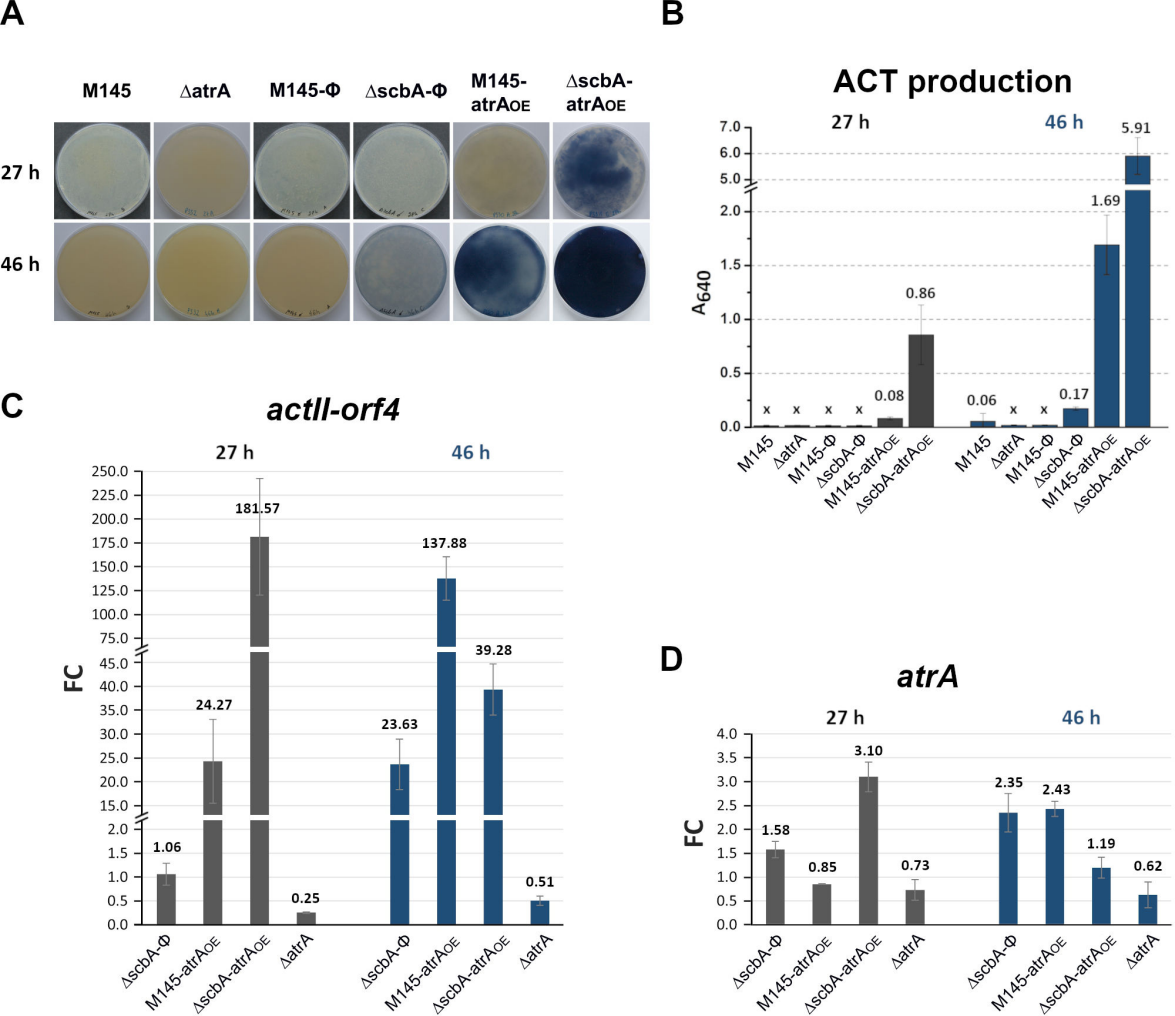
Phenotypes (**A**), actinorhodin yield (**B**), and RT-qPCR transcriptional analysis of the *actII-orf4* (**C**) and *atrA* (**D**) genes in *S. coelicolor* A3(2) mutant strains. Two hundred microliters of spore suspension in water (OD_600_ = 0.3) was spread on solid 79NG medium covered with a cellophane disk and grown at 30°C. “X” in panel B denotes no detectable production of ACT. For the *atrA* gene (panel D), qPCR primers were designed to specifically detect only the native transcript (from the *atrA* promoter) and not that resulting from overexpression. Gene expression fold changes (FC) for strains in panels C and D were calculated as comparisons against their respective control strains: M145 for Δ*atrA* and M145-Φ for M145-*atrA_OE_,* Δ*scbA*-Φ, and Δ*scbA-atrA*_OE_.

According to the hypothesis that GBLs would indeed bind to AtrA and inhibit its activity, the effects of *atrA* overexpression should be reinforced in a double mutant Δ*scbA-atrA_OE_* (not producing GBLs). Indeed, we have observed higher production of ACT and stronger upregulation of *actII-orf4* in Δ*scbA-atrA_OE_* than in the M145-*atrA_OE_* strain (Fig. 4 and 5); yet, as we have demonstrated, AtrA does not bind GBLs. This observation has led us to the conclusion that ACT biosynthesis was impacted by some other factor induced by GBLs. Since GBLs directly activate coelimycin biosynthesis, we have decided to investigate the possible inhibitory role of CPK on ACT biosynthesis.

Detailed phenotypic observations and RT-qPCR results were as follows:

#### Δ*atrA*

The deletion mutant exhibited a complete loss of ACT production when it was grown directly as a spot or on cellophane on solid medium 79NG (Fig. 4 and 5A and B), confirming that AtrA is required for the cascade of actinorhodin synthesis activation. qPCR revealed that the *actII-orf4* transcription of this strain decreased in comparison to that of M145 (Fig. 5C). Although Δ*atrA* was delayed in RED production, it synthesized plenty of this pigment at later time points (Fig. 4).

#### M145*-atrA*_OE_

This strain produced ACT much earlier than M145-Φ (Fig. 4 and 5A and B). The transcription of *actII-orf4* matched these phenotypes (Fig. 5C).

#### Δ*scbA-atrA*_OE_

In a background free of GBLs, the effects of *atrA* overexpression were much more pronounced than in M145-*atrA_OE_*. This became evident as early as at the 18 h time point when the Δ*scbA-atrA*_OE_ spot culture started to precociously produce actinorhodin and continued throughout the 96 h of growth to accumulate the highest levels of ACT among the studied strains (Fig. 4). This strain was also the most potent ACT producer on cellophane disks, with enormous yields (Fig. 5A and B). At the transcriptional level, at the 27 h time point, the Δ*scbA-atrA*_OE_ double mutant’s fold change of *actII-orf4* transcript relative to M145-Φ was much higher than that of the M145-*atrA*_OE_ strain (Fig. 5C). Interestingly, at the 46 h time point, the increase in *actII-orf4* transcription in the Δ*scbA-atrA*_OE_ strain was not as strong as that in the M145-*atrA*_OE_ strain, despite the greatest increase in ACT yield (near-black color of agar plates) in the double mutant (Fig. 5A through C). We suspect that the accumulation of ACT at such a high concentration may interfere with DNA binding by AtrA. Indeed, the addition of ACT prevented the formation of protein-DNA complexes in the case of both AtrA and HypR, as shown by EMSA (Fig. S5).

#### Δ*scbA*-Φ

Even the deletion of the GBL synthase *scbA* gene alone increased *actII-orf4* transcription and ACT production in comparison to the wild-type strain. This effect could be observed on cellophane discs at the 46 h time point (Fig. 5A through C). When bacteria were grown as spots, ACT was produced somewhat faster in M145-Φ than in Δ*scbA*-Φ (Fig. 4).

**Fig 6:**
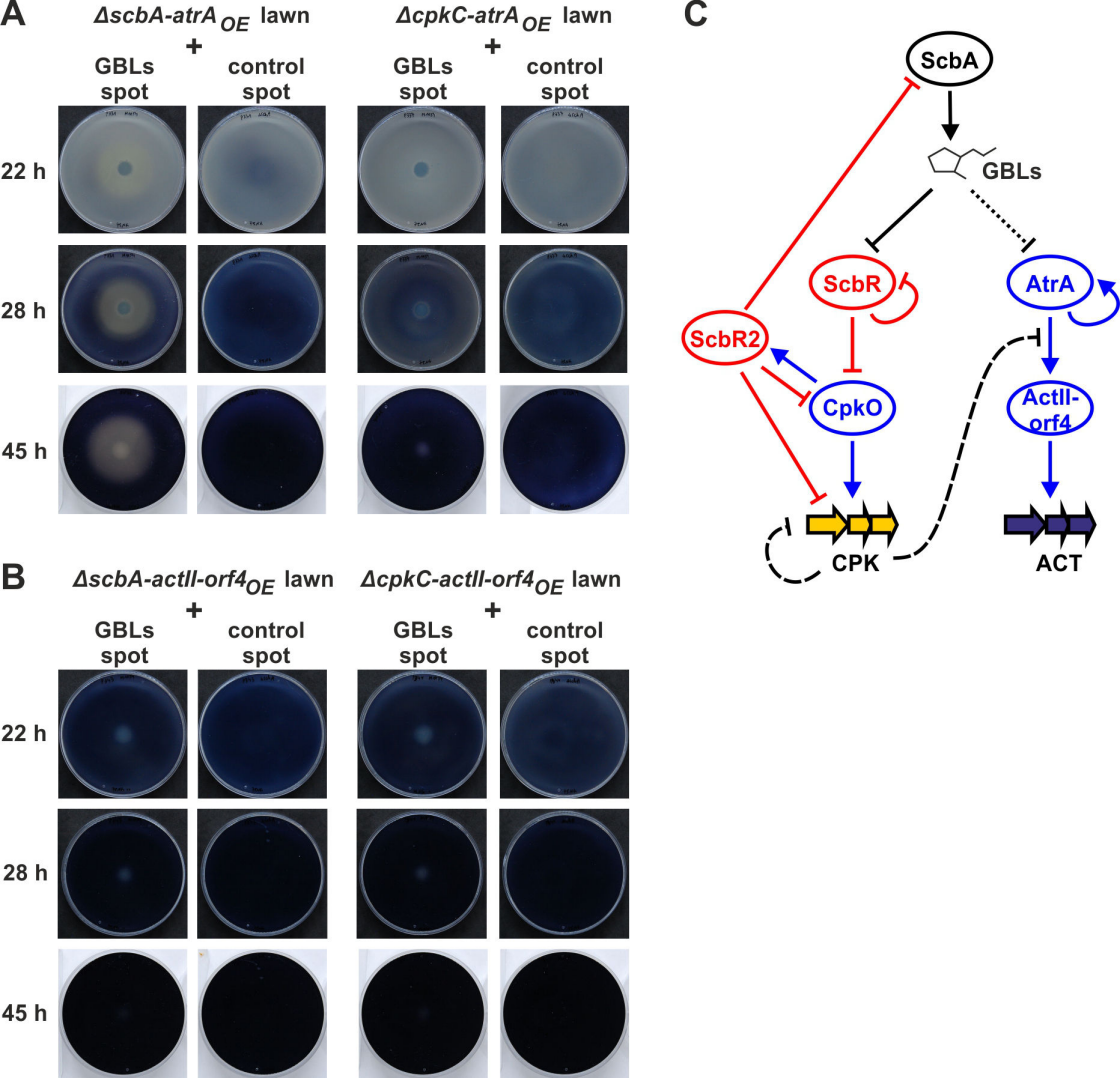
GBL-mediated activation of coelimycin synthesis and inhibition of actinorhodin synthesis. (**A and B**) Effect of GBL-rich extract on *S. coelicolor* A3(2) overproducing ACT due to either *atrA* (**A**) or *actII-orf4* (**B**) overexpression. Strains Δ*scbA-atrA*_OE_ and Δ*scbA-actII-orf4*_OE_ are unable to initiate CPK production without exogenous GBLs. Strains Δ*cpkC-atrA*_OE_ and Δ*cpkC-actII-orf4*_OE_ are unable to produce CPK due to the CPK synthase knockout. (**C**) Schematic representation of the influence of GBLs on CPK and ACT synthesis. The black arrow represents GBL synthesis. The black line with a bar represents the dissociation of ScbR from its target (*cpkO* promoter) upon GBL binding, leading to derepression of CpkO. Transcription activators and repressors are marked in blue and red, respectively, and their direct actions are marked with corresponding solid lines (blue arrows and red lines with bars). Dashed line indicates inhibition by CPK (available data do not allow to state whether the inhibition of AtrA activity is direct or indirect). Dotted line represents indirect inhibition of *atrA* transcription by GBLs (possibly by CPK inhibiting AtrA autoactivation).

Since AtrA was shown by EMSA to bind specifically to a DNA probe encompassing its own promoter region (Fig. S1 and S5), potential autoregulation was investigated by RT-qPCR (Fig. 5D). Deletion of the *atrA* gene did not significantly change the transcription. On the other hand, the deletion of *scbA* resulted in the increase of *atrA* transcription at the 46 h time point, indicating an indirect inhibitory effect of GBLs (dotted line in Fig. 6B). In Δ*scbA-atrA*_OE_ and M145-*atrA*_OE_ strains, general upregulation of the native *atrA* transcript was observed, yet with peaks at different time points (Fig. 5D).

As shown by phenotypic observations and qRT-PCR results, the effect of AtrA overexpression on actinorhodin production was more pronounced in the Δ*scbA* background than in the wild-type. To test the possibility that AtrA inhibition was associated with the induction of coelimycin production by GBLs, we compared the effect of GBLs addition to Δ*scbA-atrA*_OE_ and Δ*cpkC-atrA*_OE_ mutants grown as confluent lawns. The latter strain is unable to produce CPK due to the disruption of one of the main polyketide synthase subunits. The GBL-containing extract promoted CPK synthesis and prevented ACT production, while the Δ*scbA* extract produced no distinct phenotype (Fig. 6A, left panel). In the case of Δ*cpkC-atrA*_OE_, which cannot produce CPK, both the GBL-rich and the control extracts produced no visible ACT-depleted area (Fig. 6A, right panel). This suggests that ACT production is inhibited by CPK molecule(s), or its synthesis, and not the GBLs.

The same test was done with strains overexpressing *actII-orf4*, which is a direct target of AtrA in the activatory cascade. Notably, ACT production in these strains started earlier than in AtrA overproducers. GBL addition before the onset of ACT production (after 6 h of growth) did not inhibit ACT production by Δ*scbA-actII-orf4*_OE_ and Δ*cpkC-actII-orf4*_OE_ (Fig. 6B). Thus, we can conclude that the inhibitory activity of CPK targets AtrA, and not the downstream regulator ActII-orf4. The GBL-mediated regulation of coelimycin and actinorhodin synthesis is summarized in Fig. 6C.

When the wild-type strain (M145) cultures were exposed to GBLs at the growth stage at which CPK synthesis was already switched off (48 h), no changes in ACT production were observed (Fig. S6).

To check if it was the extracellular form of coelimycin or its intracellular precursor(s) (preCPK) that were responsible for the inhibition of ACT production, GBL-containing extract was spotted on the lawns of two strains: (i) M145-*atrA_OE_* overproducing AtrA in the background of wild-type and (ii) Δ*cpkF-atrA*_OE_ overproducing AtrA, unable to produce yCPK due to the lack of the transporter CpkF, but producing preCPK (36). After GBL addition, ACT inhibition zones appeared on both plates, indicating the involvement of preCPK (Fig. S7). Nevertheless, this result does not allow the conclusion of whether extracellular CPK is involved in this process.

**Fig 7:**
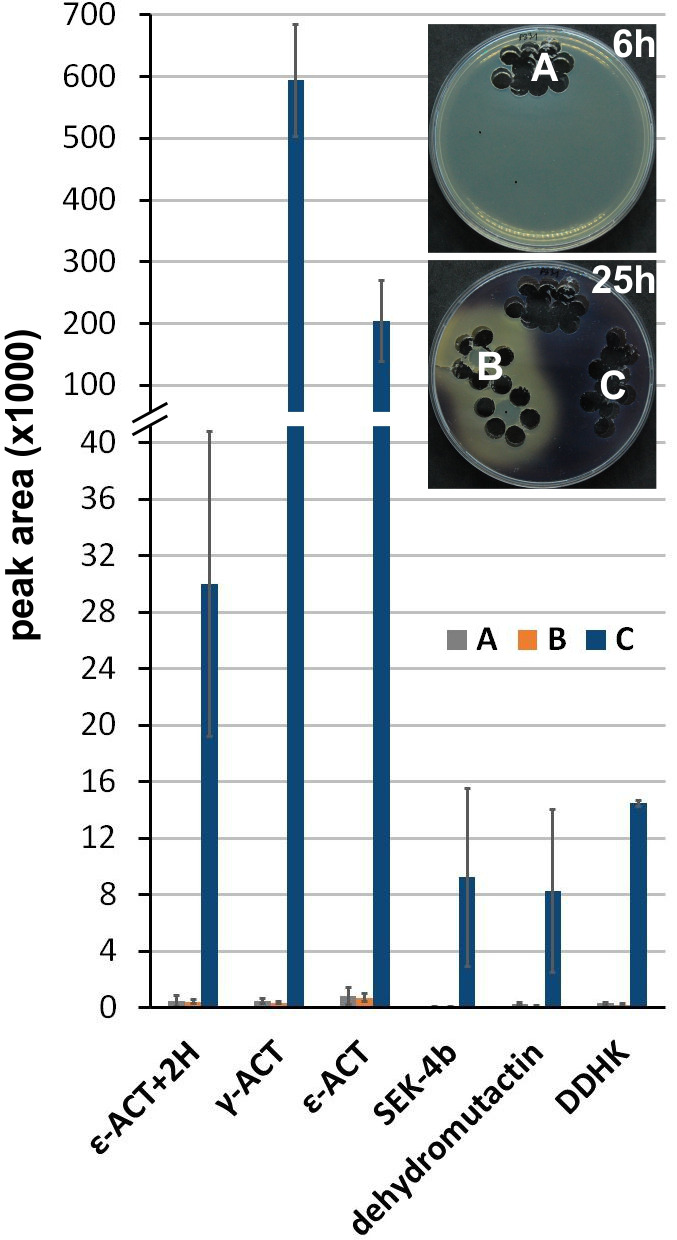
Compounds from the actinorhodin pathway detected by LC-MS/MS in the extracts from a plate culture of Δ*scbA-atrA*_OE_, quantified as peak areas from precursor extracted ion chromatograms. Sample A: control cut out after 6 h of growth, before GBL addition; sample B: cut out after 25 h of growth from the zone of ACT depletion; sample C: cut out after 25 h of growth from the outer zone not influenced by GBLs.

To address the question of whether ACT synthesis is stopped completely in the zone of ACT depletion, or if some unpigmented intermediates (40, 50) can be found there, metabolite identification and their relative quantification via LC-MS/MS were performed similarly as in Bai et al. (40). A control sample of agar for extraction was cut out from a 6 h old culture of ∆*scbA-atrA*_OE_ before the onset of ACT production and before GBLs addition (Fig. 7, sample A). After cutting out the control samples, GBL-rich extract was added. Further samples were taken from the same plate after 25 h of growth from two zones: the zone of GBLs diffusion (Fig. 7, sample B) and from the outer zone not influenced by GBLs (Fig. 7, sample C). Actinorhodin, its intermediate, and shunt products were detected only in the outer zone without external GBLs (Fig. 7; Fig. S8; Table S6).

### Effects of *atrA* deletion and overexpression on the proteome of *S. coelicolor* A3(2)

**Fig 8:**
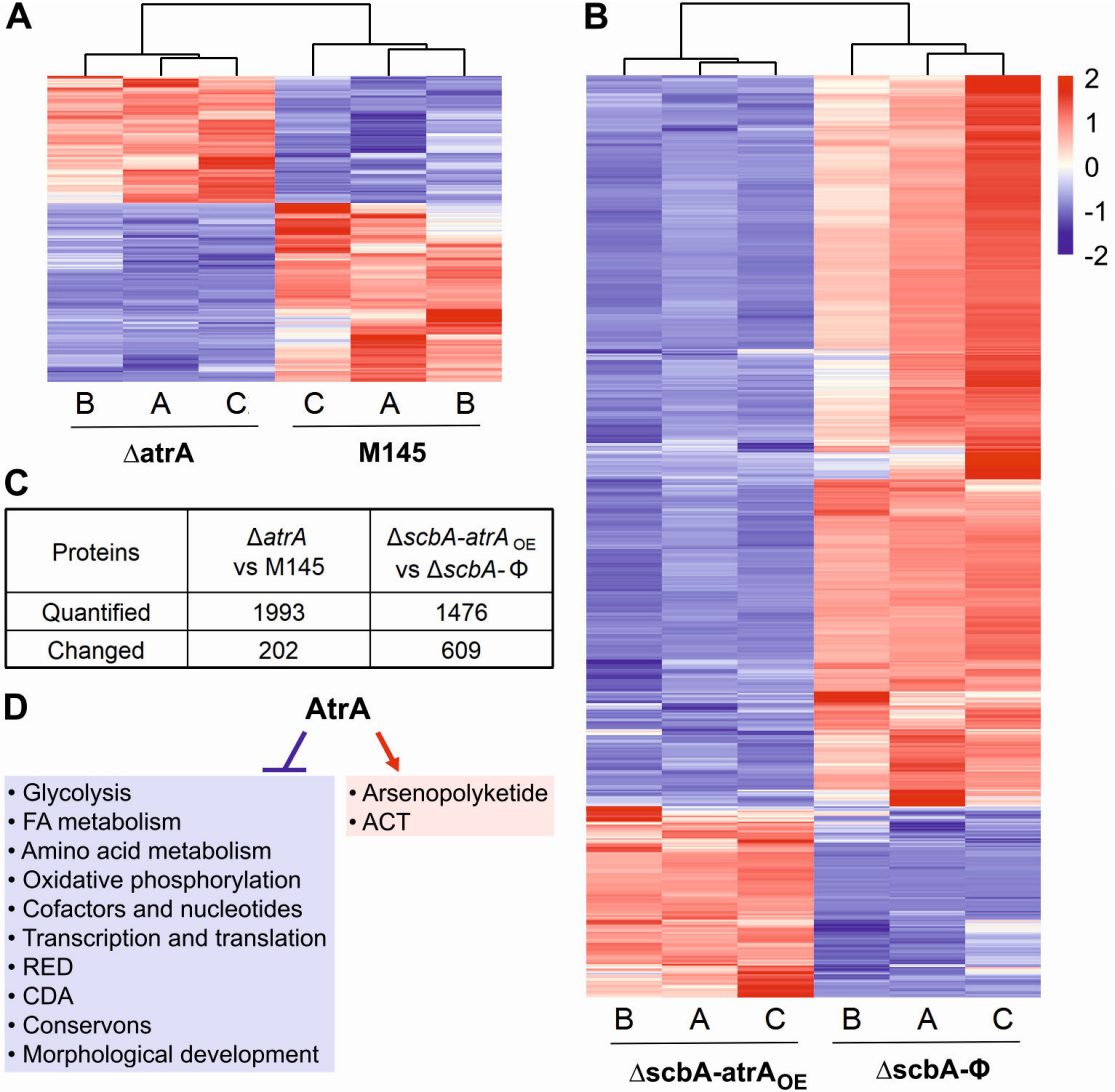
Statistically significant abundance changes in proteins identified with at least one unique peptide, statistical significance (*q* value ≤ 0.05), and mean abundance difference ratios of at least 1.5 between strains in proteomic analyses. The data for heatmaps were row-wise z-score normalized, and hierarchical clustering of columns was performed to group samples with similar abundance patterns. (**A**) Δ*atrA* strain vs M145 strain at the 50 h time point, (**B**) Δ*scbA-atrA*_OE_ strain vs Δ*scbA*-Φ strain at the 27 h time point, (**C**) number of proteins quantified and changed in the two comparisons, and (**D**) summary of the AtrA regulon depicted by the proteomics approach.

In order to better understand the extent of AtrA regulatory influence over the metabolic processes of *S. coelicolor* A3(2), we have performed two comparative proteomic analyses: (I) Δ*atrA* strain vs M145 strain at the 50 h time point, and (II) Δ*scbA-atrA*_OE_ strain vs Δ*scbA-*Φ strain at the 27 h time point. Contrary to the gene deletion analysis I, comparison II was designed to elicit stronger effects by forced *atrA* overexpression in the Δ*scbA* strain*,* which does not produce GBLs, coelimycin, and *cpk* cluster proteins. The lists of proteins quantified and changed are in Tables S7 and S8, respectively, for comparison I, and in Tables S9 and S10 for comparison II. Figure 8 shows the summary of proteomic results.

### Secondary metabolism, precursor flux, and primary metabolism are interconnected

#### Actinorhodin BGC (SCO5071–SCO5092)

**Fig 9:**
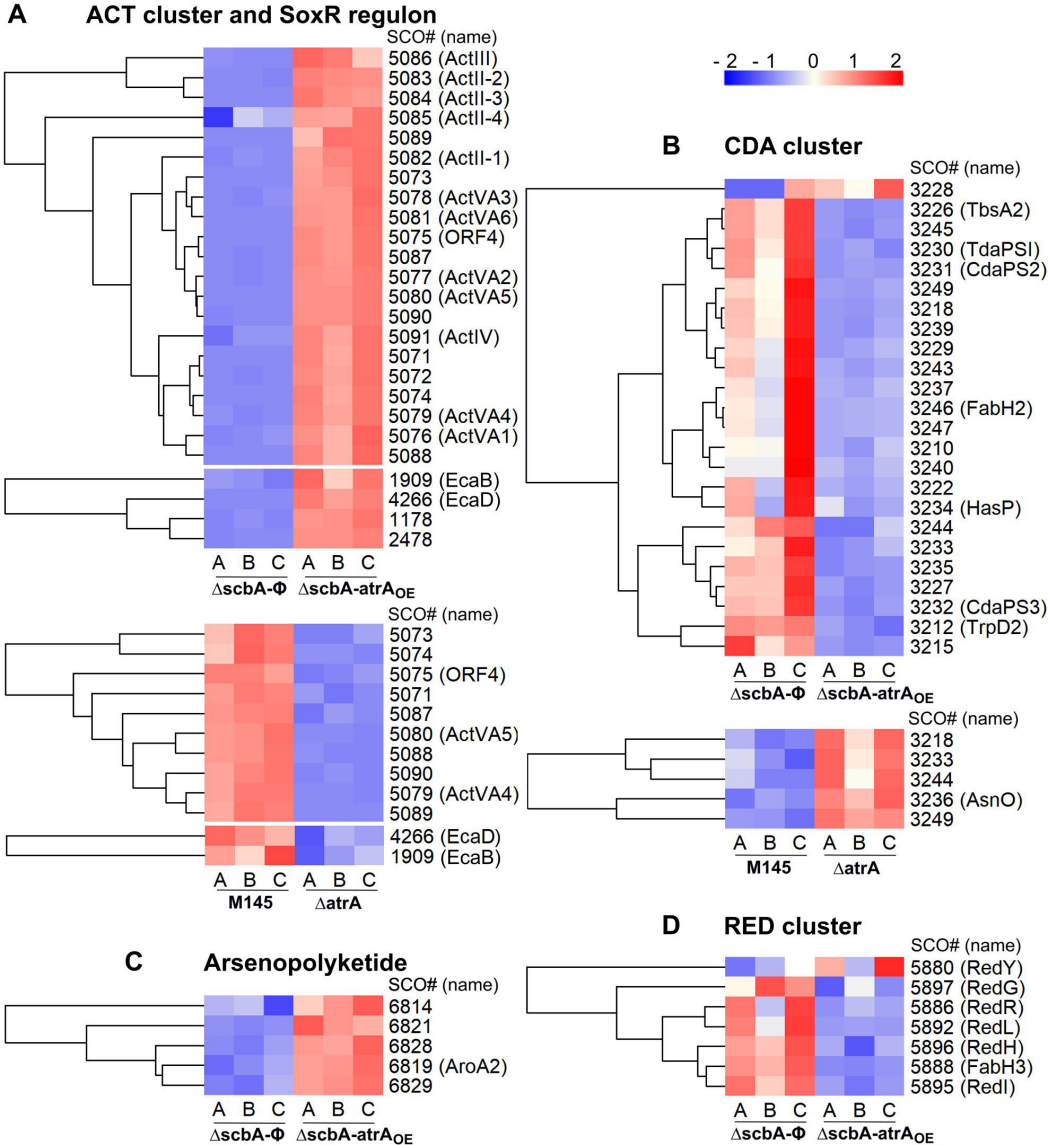
Statistically significant abundance changes in secondary metabolism proteins. (**A**) Actinorhodin BGC and SoxR regulon, (**B**) calcium-dependent antibiotic BGC, (**C**) arsenopolyketide BGC, and (**D**) undecylprodigiosin BGC. Where applicable, results from one or both proteomic comparisons are shown: Δ*atrA* vs M145 strain at the 50 h time point and/or Δ*scbA-atrA*_OE_ vs Δ*scbA*-Φ strain at the 27 h time point. The data were row-wise *z*-score normalized, and hierarchical clustering of rows was performed to group proteins with similar abundance patterns.

AtrA was discovered as the higher-level inducer of actinorhodin synthesis, activating the *actII-orf4* gene (19), which is directly responsible for transcriptional activation of both the early and late ACT biosynthetic genes (51). No visible ACT production in the Δ*atrA* strain (Fig. 4 and 5A and B) was reflected in strongly downregulated *act* cluster proteins (Fig. 9A). On the other hand, the robust ACT overproduction in the Δ*scbA-atrA*_OE_ strain was evident in *act* cluster protein upregulation (Fig. 9A). Actinorhodin was shown to modulate the SoxR protein for activation of a five-protein regulon (epimerase/dehydratase SCO1178, monooxygenase EcaB—SCO1909, oxidoreductase SCO2478, oxidoreductase EcaD—SCO4266, ABC transport protein EcaA—SCO7008) that was proposed to aid in ACT detoxification (52). Indeed, we observed SCO1909 and SCO4266 to be downregulated in the Δ*atrA* strain, while almost the whole regulon was upregulated in the Δ*scbA-atrA*_OE_ strain (Fig. 9A).

**Fig 10:**
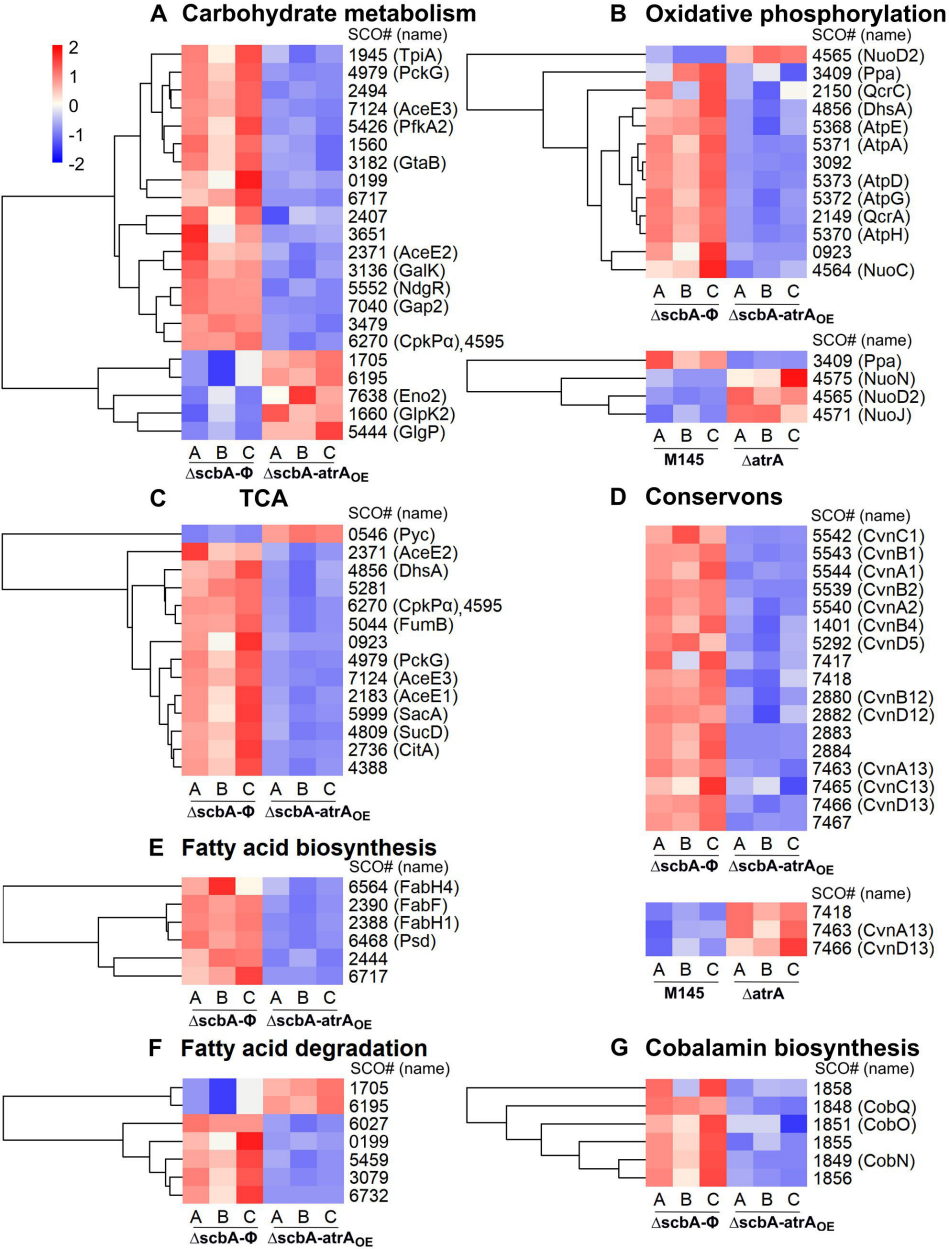
Statistically significant abundance changes in primary metabolism proteins and conservons. (**A**) Carbohydrate metabolism, (**B**) oxidative phosphorylation, (**C**) tricarboxylic acid cycle, (**D**) conservons, (**E**) fatty acid biosynthesis, (**F**) fatty acid degradation, and (**G**) cobalamin biosynthesis. Where applicable, results from one or both proteomic comparisons are shown: Δ*atrA* vs M145 strain at the 50 h time point and/or Δ*scbA-atrA*_OE_ vs Δ*scbA*-Φ strain at the 27 h time point. The data were row-wise *z*-score normalized, and hierarchical clustering of rows was performed to group proteins with similar abundance patterns. In panel **D**, the rows were not clustered.

Actinorhodin synthesis precursors are derivatives of acetyl-CoA. In our data, proteins of acetyl-CoA source processes (glycolysis and fatty acid degradation) were generally downregulated upon *atrA* overexpression, with some exceptions, that is, acetyl-CoA synthetase SCO6195. On the other hand, proteins of the main acetyl-CoA sink—the tricarboxylic acid cycle (TCA)—were also mainly downregulated in the Δ*scbA-atrA*_OE_ strain (Fig. 10). The plausible reason for TCA cycle downregulation may be the scarcity of acetyl-CoA, which is actively being used up for forced ACT synthesis in the *atrA* overexpression strain. Consequently, downregulation of the TCA cycle correlated well with generally downregulated amino acid biosynthesis/metabolism proteins (Fig. S9).

ACT production was also associated with strong downregulation of intracellular ATP (53). Indeed, we have observed oxidative phosphorylation pathway proteins to be generally downregulated upon *atrA* overexpression (robust ACT production) and upregulated in Δ*atrA* (no ACT synthesis) (Fig. 10B).

#### Calcium-dependent antibiotic BGC (SCO3210–SCO3249)

CDA is a peptidic antibiotic, which is synthesized from acetyl-CoA and amino acids: glutamate, glycine, asparagine, aspartate, serine, threonine, and tryptophan as precursors (54). CDA cluster proteins were downregulated upon *atrA* overexpression and upregulated in Δ*atrA* (Fig. 9B). Direct control of the CDA cluster SARP activator CpkR by AtrA was suggested (8), although the AtrA binding site was detected within the coding sequence of the *cdaR* gene (25). On the other hand, proteins belonging to glycine, serine, and threonine metabolism processes were downregulated in *atrA* overexpression and deletion strains (Fig. S9). Glutamate metabolism proteins—SCO1483-84 (CarB-A), SCO2198 (GlnA), SCO2789 (GlmS2), SCO4078 (PurQ), and SCO4086 (PurF)—were also downregulated in *atrA* overexpression strain. It is worth noting that CDA production was also proposed to cause a drop in cellular ATP generation (55); however, we have observed downregulation of oxidative phosphorylation pathway proteins in *atrA* overexpression (decreased CDA production) and upregulation in Δ*atrA* (increased CDA production) (Fig. 10B). A plausible explanation for this discrepancy is that the effects of ACT production on ATP synthesis are much more pronounced and mask those of CDA.

#### Arsenopolyketide BGC (SCO6812–SCO6837)

The function of this polar polyketide is yet unknown (56). We have detected five proteins from this cluster, all of which were upregulated in the *atrA* overexpression strain (Fig. 9C).

#### Undecylprodigiosin BGC (SCO5877–SCO5900)

Biosynthetic gene cluster (*red*) proteins could only be detected in the proteomic comparison of Δ*scbA-atrA*_OE_ vs Δ*scbA-*Φ strain. With one exception (RedY), all other Red proteins were generally downregulated upon *atrA* overexpression (Fig. 9D). We speculate that *red* cluster downregulation is not a direct consequence of *atrA* overexpression, as an earlier study has shown no change in RED synthesis in Δ*atrA* (19). The precursors for RED production include proline, glycine, serine, and acetyl-CoA (54). In our data, we have seen downregulation of the relevant amino acid biosynthesis/degradation proteins. These proteins were SCO1087, SCO6466, SCO3479, SCO4366 (SerC), SCO4837 (GlyA), SCO5469 (SdaA), SCO5470 (GlyA2), SCO5519, and SCO6800 (Fig. S9).

#### Conservons

*S. coelicolor* A3(2) genome encodes 13 conserved operons spread throughout the chromosome (57). They structurally resemble mammalian membrane-associated G-protein-coupled receptor systems and may act as pathway-specific (58) or global regulators (59). In our data, all of the conservon proteins were downregulated upon *atrA* overexpression and upregulated in Δ*atrA* (Fig. 10D). This could indicate the existence of a universal mechanism for conservon expression regulation, perhaps by AtrA itself or some downstream regulator.

Among other processes downregulated upon AtrA overexpression were transcription and translation (Fig. S10A), synthesis of cobalamin (Fig. 10G), other cofactors and nucleotides (Fig. S10B), which could impact the energy state of the cell (lower synthesis of ATP, NAD+) and limit nucleic acid synthesis. Morphological development was also deregulated in Δ*scbA-atrA_OE_* (Fig. S11). The abundances of the phosphotransferase system (PTS) permease complex components EI (PtsI, SCO1391), and IIC (NagE2, SCO2907) (20) were decreased upon AtrA overexpression and not changed in Δ*atrA* (Table S7). Additionally, in Δ*scbA-atrA_OE_*, we have observed downregulation of the extracellular contractile injection system (SCO4246, SCO4252, SCO4253), which has been shown to regulate proper aerial hyphae formation and sporulation (60).

## DISCUSSION

Because of their complex life cycle and diverse ecological niches as saprophytes, *Streptomyces* must integrate multiple signals and adapt to variable environmental conditions. They have become an outstanding model for genome regulation and bacterial communication studies. The majority of previous studies focused on γ-butyrolactone/butenolide regulation of the synthesis of a particular natural product (transcription regulation of its BGC). However, *in silico* predictions reveal the existence of many putative GBL TetR-like receptors in each *Streptomyces* genome. What is more, an accumulating body of evidence suggests that cell-to-cell communication may control multiple primary and secondary metabolism pathways (61).

Our results underline the importance of considering the quorum sensing cascades while optimizing secondary metabolite production and designing perfect chassis for heterologous BGC expression. In some cases, disabling the signaling molecule synthesis may prove beneficial for yield enhancement of selected compounds. In *S. lividans*, deletion of the GBL synthase gene enhanced RED and ACT overproduction, which was already stimulated by constitutive expression of respective *S. coelicolor* activatory genes, *redD* and *actII-orf4* (62). Similarly, here, the activatory effect of *atrA* overexpression on ACT production was stronger in the Δ*scbA* background.

### CPK inhibits ACT synthesis

The quorum sensing system of *S. coelicolor* A3(2) is based on GBLs, which are necessary to unlock the biosynthesis of an early secondary metabolite—coelimycin. We have shown recently that intracellular precursor(s) of CPK act as a negative feedback autoregulator (36). Our current results provide evidence that CPK biosynthesis induced by GBLs inhibits ACT production. There are two possible explanations. One hypothesis is that preCPK may interact as a ligand with either AtrA or another unknown regulator. In *S. globisporus,* a lidamycin production intermediate was reported to interact with an AtrA homolog, dissociate it from DNA, and deactivate lidamycin BGC transcription (32). According to our results, coelimycin intermediate can regulate not only its own but also ACT biosynthesis. Since the direct *act* cluster activator, ActII-orf4, was insensitive to CPK induction by GBLs (Fig. 6), we conclude that AtrA is the target of inhibition by CPK. However, the exact molecular interactions leading to this effect require further investigation. Alternatively, the two polyketides, CPK and ACT, simply compete for biosynthetic precursors. However, as we have shown by metabolomic analysis of *atrA* overexpression with GBLs addition (Fig. 7), ACT production is completely abolished in the GBL-influenced zones of the culture. Based on this evidence, simple competition for the precursor supply seems to be of little significance, marginal to the regulation by preCPK as a putative ligand(s).

### Pleiotropic regulation by AtrA

We conclude that AtrA belongs to the GBL-dependent regulatory network, although it does not directly interact with GBLs. We also show that this protein is an autoactivator and that its transcription is indirectly inhibited by GBLs, possibly through preCPK as a ligand. AtrA homolog in *Streptomyces roseosporus* is a part of a GBL signaling pathway in which GBLs induce ArpA dissociation from the *adpA* gene promoter, resulting in its derepression. AdpA then activates *atrA,* which in turn activates daptomycin synthesis by binding to the *dptE* promoter. AtrA autoactivation of *atrA* gene transcription serves as a positive feedback loop in this pathway (31).

We confirmed the crucial role of AtrA in the activation of ACT biosynthesis and revealed its pleiotropic influence on the regulation of other secondary metabolites. Moreover, general downregulation of primary metabolic pathways by AtrA was observed on the proteomic level. It is important to remember that some of these effects may result from drastic changes in ACT production by the mutant strains.

The intergenic region of *atrA-SCO4119* is bound by ScbR2 (9) and DasR (63) – the master regulator of the nutrient signaling by GlcNAc, which links the onset of antibiotic production with morphological differentiation (22). Moreover, the effect of GlcNAc on the DNA binding by DasR is medium-dependent (63). DasR and AtrA share some targets and apparently act in opposite ways on *actII-orf4* and *nagE2* transcription. Direct activation of *nagE2* by AtrA was shown previously under starvation conditions (MM mannitol agar) (20). On the contrary, here, on a rich medium 79NG, *atrA* overexpression in a GBL-free strain lowered the abundances of NagE2 and PtsI, indicating repression. Similarly, negative regulation of *nagE2* and *ptsI* by AtrA was observed by Al-Tarawni on rich R5 medium (25).

Based on ChIP-seq results, two nucleotide sequence motifs bound by AtrA were found, indicating two modes of interaction with DNA (25). Proteins with changed abundances, which have AtrA binding motifs in their gene promoter regions, are highlighted in Tables S8 and S10. The possible targets of direct activation by AtrA which were not reported previously include: SCO0500 (DUF5302 domain-containing protein), SCO1755 (transferase), SCO4826 (membrane protein), and SCO7590 (KatA2, catalase). The following are possible new targets of direct negative regulation by AtrA: SCO0929 (SAM-dependent methyltransferase), SCO3119 (histidine kinase), SCO3543 (TopA, DNA topoisomerase I), SCO3761 (uncharacterized protein), SCO3794 (glycosyl transferase), SCO5249 (nucleotide-binding protein), and SCO5357 (Rho, transcription termination factor, ATP-dependent helicase).

### Challenges in accurate identification of GBL receptors

This work explores the methodology for accurate identification (or reassessment) of GBL receptors. We have shown that EMSA may produce false-positive results in GBL receptor identification studies, as seems to be the case for AtrA (this study) and SlbR (18). Pull-down approach coupled with HPLC-MS is a useful, direct test of specific protein-GBL interactions.

We propose that these methods should ideally be complemented by an *in vivo* reporter assay. However, this requires precise knowledge of the bound DNA sequence. The two-plasmid approach presented here makes the system more flexible and allows the use of different combinations of plasmids carrying proteins of interest and their target DNA fragments. Here, we could not verify the influence of GBLs on DNA binding by SlbR, as GFP fluorescence was not reduced by this protein. This result suggests that either site R and site A are not bound by SlbR, or the spatial organization of the complex does not interfere with GFP expression. Indeed, an initial experiment aimed at the selection of the binding site of AtrA for which the GFP fluorescence reduction can be observed showed different effects depending on the identity and orientation of the oligonucleotides tested (Fig. S4).

We have developed an easy test for the detection of GBLs exploiting Δ*scbA* as a convenient reporter strain. Addition of GBLs induces production of CPK, visible as the yellow pigment either in the liquid culture or on the solid medium. The bioassay could potentially be used to screen for GBL production simultaneously in multiple different *Streptomyces* species/strains and, consequently, also for new inter-species interactions. However, it is uncertain whether the potential GBLs would be compatible with ScbR as a receptor and present in sufficient amounts.

### Putative new GBLs

Predicted abundant production of GBLs by Δ*cpkO* and M1154 strains, both lacking the ScbR2 repression of the GBL synthase gene *scbA,* was confirmed by HPLC-MS analysis. Interestingly, the extracts from both strains revealed the presence of three potentially new gamma-butyrolactones (tentatively named SCB9, SCB10, and SCB11), which were not reported by Sidda et al. (35). The amount of sample material was too scarce to undertake their structure elucidation. Previously, Efremenkova and co-workers identified six GBLs from *S. coelicolor* A3(2) (3, 46) (Table S5). Three of them (Acl-2a, Acl-2b, and Acl-2d) are stereoisomers of SCB1 (64). Acl-2c has the same molecular mass as SCB1 and SCB2 but a different side chain structure. Acl-1a and Acl-1b are stereoisomers of SCB3. It cannot be excluded that putative SCB9, which has the same molecular mass as SCB1 and SCB2, corresponds to one of the Acl-2 series compounds. Similarly, putative SCB10 and SCB11, which have the same molecular mass as SCB3 and SCB7, may correspond to Acl-1a and Acl-1b.

## Data Availability

All the data generated and analyzed in this study are included in this article and in the supplemental files. The mass spectrometry proteomics data have been deposited to the ProteomeXchange Consortium via the PRIDE partner repository with the data set identifier PXD066756 and 10.6019/PXD066756. LC-MS/MS data are available in the Zenodo repository under the identifier https://doi.org/10.5281/zenodo.18350093.

## SUPPLEMENTAL MATERIAL

Supplemental material mbio.00630-26-s0001.pdf Supplemental text, tables, and figures.

Supplemental tables mbio.00630-26-s0002.xlsx Tables S6 to S10.

ASM does not own the copyrights to Supplemental Material that may be linked to, or accessed through, an article. The authors have granted ASM a non-exclusive, world-wide license to publish the Supplemental Material files. Please contact the corresponding author directly for reuse.

## References

[1] Barka EA, Vatsa P, Sanchez L, Gaveau-Vaillant N, Jacquard C, Meier-Kolthoff JP, Klenk H-P, Clément C, Ouhdouch Y, van Wezel GP. 2016. Taxonomy, physiology, and natural products of Actinobacteria. Microbiol Mol Biol Rev 80:1–43. doi:10.1128/MMBR.00019-15 26609051 PMC4711186

[2] Olanrewaju OS, Babalola OO. 2019. Streptomyces: implications and interactions in plant growth promotion. Appl Microbiol Biotechnol 103:1179–1188. doi:10.1007/s00253-018-09577-y 30594952 PMC6394478

[3] Efremenkova OV. 2016. A-factor-like autoregulators. Russ J Bioorg Chem 42:457–472. doi:10.1134/S1068162016050058

[4] Zhou S, Bhukya H, Malet N, Harrison PJ, Rea D, Belousoff MJ, Venugopal H, Sydor PK, Styles KM, Song L, Cryle MJ, Alkhalaf LM, Fülöp V, Challis GL, Corre C. 2021. Molecular basis for control of antibiotic production by a bacterial hormone. Nature 590:463–467. doi:10.1038/s41586-021-03195-x 33536618

[5] Polkade AV, Mantri SS, Patwekar UJ, Jangid K. 2016. Quorum sensing: an under-explored phenomenon in the phylum Actinobacteria. Front Microbiol 7:131. doi:10.3389/fmicb.2016.00131 26904007 PMC4748050

[6] Rutherford ST, Bassler BL. 2012. Bacterial quorum sensing: its role in virulence and possibilities for its control. Cold Spring Harb Perspect Med 2:a012427. doi:10.1101/cshperspect.a012427 23125205 PMC3543102

[7] van Keulen G, Dyson PJ. 2014. Production of specialized metabolites by Streptomyces coelicolor A3(2). Adv Appl Microbiol 89:217–266. doi:10.1016/B978-0-12-800259-9.00006-8 25131404

[8] van der Heul HU, Bilyk BL, McDowall KJ, Seipke RF, van Wezel GP. 2018. Regulation of antibiotic production in Actinobacteria: new perspectives from the post-genomic era. Nat Prod Rep 35:575–604. doi:10.1039/c8np00012c 29721572

[9] Li X, Wang J, Li S, Ji J, Wang W, Yang K. 2015. ScbR- and ScbR2-mediated signal transduction networks coordinate complex physiological responses in Streptomyces coelicolor. Sci Rep 5:1–14. doi:10.1038/srep14831 PMC4595836 26442964

[10] Takano E, Kinoshita H, Mersinias V, Bucca G, Hotchkiss G, Nihira T, Smith CP, Bibb M, Wohlleben W, Chater K. 2005. A bacterial hormone (the SCB1) directly controls the expression of a pathway-specific regulatory gene in the cryptic type I polyketide biosynthetic gene cluster of Streptomyces coelicolor*.* Mol Microbiol 56:465–479. doi:10.1111/j.1365-2958.2005.04543.x 15813737

[11] Takano E, Chakraburtty R, Nihira T, Yamada Y, Bibb MJ. 2001. A complex role for the gamma-butyrolactone SCB1 in regulating antibiotic production in Streptomyces coelicolor A3(2). Mol Microbiol 41:1015–1028. doi:10.1046/j.1365-2958.2001.02562.x 11555283

[12] Gottelt M, Kol S, Gomez-Escribano JP, Bibb M, Takano E. 2010. Deletion of a regulatory gene within the cpk gene cluster reveals novel antibacterial activity in Streptomyces coelicolor A3(2). Microbiology (Reading) 156:2343–2353. doi:10.1099/mic.0.038281-0 20447997

[13] Pawlik K, Kotowska M, Chater KF, Kuczek K, Takano E. 2007. A cryptic type I polyketide synthase (cpk) gene cluster in Streptomyces coelicolor A3(2). Arch Microbiol 187:87–99. doi:10.1007/s00203-006-0176-7 17009021

[14] Bednarz B, Millan-Oropeza A, Kotowska M, Świat M, Quispe Haro JJ, Henry C, Pawlik K. 2021. Coelimycin synthesis activatory proteins are key regulators of specialized metabolism and precursor flux in Streptomyces coelicolor A3(2). Front Microbiol 12:616050. doi:10.3389/fmicb.2021.616050 33897632 PMC8062868

[15] Bednarz B, Kotowska M, Pawlik KJ. 2019. Multi-level regulation of coelimycin synthesis in Streptomyces coelicolor A3(2). Appl Microbiol Biotechnol 103:6423–6434. doi:10.1007/s00253-019-09975-w 31250060 PMC6667686

[16] Yang YH, Kim JN, Song E, Kim E, Oh MK, Kim BG. 2008. Finding new pathway-specific regulators by clustering method using threshold standard deviation based on DNA chip data of Streptomyces coelicolor. Appl Microbiol Biotechnol 80:709–717. doi:10.1007/s00253-008-1574-3 18654773

[17] Kim SH, Traag BA, Hasan AH, McDowall KJ, Kim B-G, van Wezel GP. 2015. Transcriptional analysis of the cell division-related ssg genes in Streptomyces coelicolor reveals direct control of ssgR by AtrA. Antonie Van Leeuwenhoek 108:201–213. doi:10.1007/s10482-015-0479-2 26002075 PMC4457907

[18] Yang YH, Song E, Kim JN, Lee BR, Kim EJ, Park SH, Kim WS, Park HY, Jeon JM, Rajesh T, Kim YG, Kim BG. 2012. Characterization of a new ScbR-like γ-butyrolactone binding regulator (SlbR) in Streptomyces coelicolor. Appl Microbiol Biotechnol 96:113–121. doi:10.1007/s00253-011-3803-4 22246527

[19] Uguru GC, Stephens KE, Stead JA, Towle JE, Baumberg S, McDowall KJ. 2005. Transcriptional activation of the pathway-specific regulator of the actinorhodin biosynthetic genes in Streptomyces coelicolor. Mol Microbiol 58:131–150. doi:10.1111/j.1365-2958.2005.04817.x 16164554

[20] Nothaft H, Rigali S, Boomsma B, Swiatek M, McDowall KJ, van Wezel GP, Titgemeyer F. 2010. The permease gene nagE2 is the key to N-acetylglucosamine sensing and utilization in Streptomyces coelicolor and is subject to multi-level control. Mol Microbiol 75:1133–1144. doi:10.1111/j.1365-2958.2009.07020.x 20487300

[21] Rigali S, Titgemeyer F, Barends S, Mulder S, Thomae AW, Hopwood DA, van Wezel GP. 2008. Feast or famine: the global regulator DasR links nutrient stress to antibiotic production by Streptomyces. EMBO Rep 9:670–675. doi:10.1038/embor.2008.83 18511939 PMC2475330

[22] Rigali S, Nothaft H, Noens EEE, Schlicht M, Colson S, Müller M, Joris B, Koerten HK, Hopwood DA, Titgemeyer F, van Wezel GP. 2006. The sugar phosphotransferase system of Streptomyces coelicolor is regulated by the GntR-family regulator DasR and links N-acetylglucosamine metabolism to the control of development. Mol Microbiol 61:1237–1251. doi:10.1111/j.1365-2958.2006.05319.x 16925557

[23] Traag BA, Kelemen GH, Van Wezel GP. 2004. Transcription of the sporulation gene ssgA is activated by the IclR-type regulator SsgR in a whi-independent manner in Streptomyces coelicolor A3(2). Mol Microbiol 53:985–1000. doi:10.1111/j.1365-2958.2004.04186.x 15255907

[24] Willemse J, Mommaas AM, van Wezel GP. 2012. Constitutive expression of ftsZ overrides the whi developmental genes to initiate sporulation of Streptomyces coelicolor. Antonie Van Leeuwenhoek 101:619–632. doi:10.1007/s10482-011-9678-7 22113698 PMC3278627

[25] Al-Tarawni AH. 2019. The biosynthesis of natural products: the characterisation and manipulation of AtrA, an evolutionarily conserved transcription factor PhD thesis, University of Leeds. https://etheses.whiterose.ac.uk/id/eprint/25151/.

[26] Ahn SK, Cuthbertson L, Nodwell JR. 2012. Genome context as a predictive tool for identifying regulatory targets of the TetR family transcriptional regulators. PLoS One 7:e50562. doi:10.1371/journal.pone.0050562 23226315 PMC3511530

[27] Hong B, Phornphisutthimas S, Tilley E, Baumberg S, McDowall KJ. 2007. Streptomycin production by Streptomyces griseus can be modulated by a mechanism not associated with change in the adpA component of the A-factor cascade. Biotechnol Lett 29:57–64. doi:10.1007/s10529-006-9216-2 17120093

[28] Chen L, Lu Y, Chen J, Zhang W, Shu D, Qin Z, Yang S, Jiang W. 2008. Characterization of a negative regulator avei for avermectin biosynthesis in Streptomyces avermitilis NRRL8165. Appl Microbiol Biotechnol 80:277–286. doi:10.1007/s00253-008-1545-8 18560830

[29] Wang W, Tian J, Li L, Ge M, Zhu H, Zheng G, Huang H, Ruan L, Jiang W, Lu Y. 2015. Identification of two novel regulatory genes involved in pristinamycin biosynthesis and elucidation of the mechanism for AtrA-p-mediated regulation in Streptomyces pristinaespiralis. Appl Microbiol Biotechnol 99:7151–7164. doi:10.1007/s00253-015-6638-6 25957493

[30] Wu W, Kang Y, Hou B, Ye J, Wang R, Wu H, Zhang H. 2023. Characterization of a TetR-type positive regulator AtrA for lincomycin production in Streptomyces lincolnensis. Biosci Biotechnol Biochem 87:786–795. doi:10.1093/bbb/zbad046 37076767

[31] Mao X-M, Luo S, Zhou R-C, Wang F, Yu P, Sun N, Chen X-X, Tang Y, Li Y-Q. 2015. Transcriptional regulation of the daptomycin gene cluster in Streptomyces roseosporus by an autoregulator, AtrA. J Biol Chem 290:7992–8001. doi:10.1074/jbc.M114.608273 25648897 PMC4367297

[32] Li X, Yu T, He Q, McDowall KJ, Jiang B, Jiang Z, Wu L, Li G, Li Q, Wang S, Shi Y, Wang L, Hong B. 2015. Binding of a biosynthetic intermediate to AtrA modulates the production of lidamycin by Streptomyces globisporus*.* Mol Microbiol 96:1257–1271. doi:10.1111/mmi.13004 25786547

[33] Hasan AH. 2015. Specific and global networks of gene regulation in Streptomyces coelicolor PhD thesis, University of Leeds

[34] Gomez-Escribano JP, Bibb MJ. 2011. Engineering Streptomyces coelicolor for heterologous expression of secondary metabolite gene clusters. Microb Biotechnol 4:207–215. doi:10.1111/j.1751-7915.2010.00219.x 21342466 PMC3818861

[35] Sidda JD, Poon V, Song L, Wang W, Yang K, Corre C. 2016. Overproduction and identification of butyrolactones SCB1-8 in the antibiotic production superhost Streptomyces M1152. Org Biomol Chem 14:6390–6393. doi:10.1039/c6ob00840b 27180870

[36] Kotowska M, Wenecki M, Bednarz B, Ciekot J, Pasławski W, Buhl T, Pawlik KJ. 2024. Coelimycin inside out - negative feedback regulation by its intracellular precursors. Appl Microbiol Biotechnol 108:531. doi:10.1007/s00253-024-13366-1 39656307 PMC11632069

[37] Kolde R. 2025. Pretty Heatmaps [R package pheatmap version 1.0.13]. CRAN: contributed packages. Comprehensive R Archive Network (CRAN). Available from: https://CRAN.R-project.org/package=pheatmap. Retrieved 23 Sep 2025.

[38] Perez-Riverol Y, Bandla C, Kundu DJ, Kamatchinathan S, Bai J, Hewapathirana S, John NS, Prakash A, Walzer M, Wang S, Vizcaíno JA. 2025. The PRIDE database at 20 years: 2025 update. Nucleic Acids Res 53:D543–D553. doi:10.1093/nar/gkae1011 39494541 PMC11701690

[39] Kieser T, Bibb MJ, Buttner MJ, Chater KF, Hopwood DA. 2000. Practical Streptomyces genetics. The John Innes Foundation.

[40] Bai C, Bayona LM, van Wezel GP. 2025. Construction and diversification of natural product biosynthetic gene clusters at high efficiency and accuracy. ACS Synth Biol 14:4574–4585. doi:10.1021/acssynbio.5c00601 41070399 PMC12645565

[41] Pfaffl MW. 2001. A new mathematical model for relative quantification in real-time RT-PCR. Nucleic Acids Res 29:e45. doi:10.1093/nar/29.9.e45 11328886 PMC55695

[42] Kotowska M, Świat M, Zarȩba-Pasławska J, Jaworski P, Pawlik K. 2019. A GntR-like transcription factor HypR regulates expression of genes associated with L-hydroxyproline utilization in Streptomyces coelicolor A3(2). Front Microbiol 10:1451. doi:10.3389/fmicb.2019.01451 31297104 PMC6608401

[43] Hsiao NH, Söding J, Linke D, Lange C, Hertweck C, Wohlleben W, Takano E. 2007. ScbA from Streptomyces coelicolor A3(2) has homology to fatty acid synthases and is able to synthesize gamma-butyrolactones. Microbiology (Reading) 153:1394–1404. doi:10.1099/mic.0.2006/004432-0 17464053

[44] Yang YH, Joo HS, Lee K, Liou KK, Lee HC, Sohng JK, Kim BG. 2005. Novel method for detection of butanolides in Streptomyces coelicolor culture broth, using a His-tagged receptor (ScbR) and mass spectrometry. Appl Environ Microbiol 71:5050–5055. doi:10.1128/AEM.71.9.5050-5055.2005 16151086 PMC1214611

[45] Wilbanks LE, Hennigan HE, Martinez-Brokaw CD, Lakkis H, Thormann S, Eggly AS, Buechel G, Parkinson EI. 2023. Synthesis of gamma-butyrolactone hormones enables understanding of natural product induction. ACS Chem Biol 18:1624–1631. doi:10.1021/acschembio.3c00241 37338162 PMC10368014

[46] Gruzina VD, Gorbatiuk EV, Efremenkova OV, Filippova SN, El’-Registan GI, Dudnik IV. 2003. A new regulatory function of the A-factor--stimulation of the streptomyces spore germination. Mikrobiologiia 72:770–774. 14768543

[47] Kotowska M, Ciekot J, Pawlik K. 2014. Type II thioesterase ScoT is required for coelimycin production by the modular polyketide synthase Cpk of Streptomyces coelicolor A3(2). Acta Biochim Pol 61:141–147. 24660171

[48] Xu G, Wang J, Wang L, Tian X, Yang H, Fan K, Yang K, Tan H. 2010. “Pseudo” γ-butyrolactone receptors respond to antibiotic signals to coordinate antibiotic biosynthesis. J Biol Chem 285:27440–27448. doi:10.1074/jbc.M110.143081 20562102 PMC2930742

[49] Noszka M, Strzałka A, Muraszko J, Kolenda R, Meng C, Ludwig C, Stingl K, Zawilak-Pawlik A. 2023. Profiling of the Helicobacter pylori redox switch HP1021 regulon using a multi-omics approach. Nat Commun 14:6715. doi:10.1038/s41467-023-42364-6 37872172 PMC10593804

[50] Marshall AP, Carlson EE. 2023. Metabolomics Reveals a “Trimeric” γ-Actinorhodin from Streptomyces coelicolor M145. Chembiochem 24:e202200757. doi:10.1002/cbic.202200757 36729633

[51] Arias P, Fernández-Moreno MA, Malpartida F. 1999. Characterization of the pathway-specific positive transcriptional regulator for actinorhodin biosynthesis in Streptomyces coelicolor A3(2) as a DNA-binding protein. J Bacteriol 181:6958–6968. doi:10.1128/JB.181.22.6958-6968.1999 10559161 PMC94170

[52] Dela Cruz R, Gao Y, Penumetcha S, Sheplock R, Weng K, Chander M. 2010. Expression of the Streptomyces coelicolor SoxR regulon is intimately linked with actinorhodin production. J Bacteriol 192:6428–6438. doi:10.1128/JB.00916-10 20952574 PMC3008532

[53] Esnault C, Dulermo T, Smirnov A, Askora A, David M, Deniset-Besseau A, Holland IB, Virolle MJ. 2017. Strong antibiotic production is correlated with highly active oxidative metabolism in Streptomyces coelicolor M145. Sci Rep 7:200. doi:10.1038/s41598-017-00259-9 28298624 PMC5427975

[54] Lejeune C, Abreu S, Guérard F, Askora A, David M, Chaminade P, Gakière B, Virolle MJ. 2024. Consequences of the deletion of the major specialized metabolite biosynthetic pathways of Streptomyces coelicolor on the metabolome and lipidome of this strain. Microb Biotechnol 17:e14538. doi:10.1111/1751-7915.14538 39093579 PMC11296114

[55] Virolle MJ. 2020. A challenging view: antibiotics play a role in the regulation of the energetic metabolism of the producing bacteria. Antibiotics (Basel) 9:83. doi:10.3390/antibiotics9020083 32069930 PMC7168255

[56] Cruz-Morales P, Kopp JF, Martínez-Guerrero C, Yáñez-Guerra LA, Selem-Mojica N, Ramos-Aboites H, Feldmann J, Barona-Gómez F. 2016. Phylogenomic analysis of natural products biosynthetic gene clusters allows discovery of arseno-organic metabolites in model Streptomycetes. Genome Biol Evol 8:1906–1916. doi:10.1093/gbe/evw125 27289100 PMC4943196

[57] Bentley SD, Chater KF, Cerdeño-Tárraga A-M, Challis GL, Thomson NR, James KD, Harris DE, Quail MA, Kieser H, Harper D, et al.. 2002. Complete genome sequence of the model actinomycete Streptomyces coelicolor A3(2). Nature 417:141–147. doi:10.1038/417141a 12000953

[58] Ozaki T, Nishiyama M, Kuzuyama T. 2013. Novel tryptophan metabolism by a potential gene cluster that is widely distributed among actinomycetes. J Biol Chem 288:9946–9956. doi:10.1074/jbc.M112.436451 23430264 PMC3617294

[59] Bonet B, Ra Y, Cantu Morin LM, Soto Bustos J, Livny J, Traxler MF. 2021. The cvn8 conservon system is a global regulator of specialized metabolism in Streptomyces coelicolor during interspecies interactions. mSystems 6:e00281-21. doi:10.1128/mSystems.00281-21 34636667 PMC8510531

[60] Vladimirov M, Zhang RX, Mak S, Nodwell JR, Davidson AR. 2023. A contractile injection system is required for developmentally regulated cell death in Streptomyces coelicolor. Nat Commun 14. doi:10.1038/s41467-023-37087-7 PMC10020575 36927736

[61] D’Alia D, Eggle D, Nieselt K, Hu W-S, Breitling R, Takano E. 2011. Deletion of the signalling molecule synthase ScbA has pleiotropic effects on secondary metabolite biosynthesis, morphological differentiation and primary metabolism in Streptomyces coelicolor A3(2). Microb Biotechnol 4:239–251. doi:10.1111/j.1751-7915.2010.00232.x 21342469 PMC3818864

[62] Butler MJ, Takano E, Bruheim P, Jovetic S, Marinelli F, Bibb MJ. 2003. Deletion of scbA enhances antibiotic production in Streptomyces lividans. Appl Microbiol Biotechnol 61:512–516. doi:10.1007/s00253-003-1277-8 12764566

[63] Świątek-Połatyńska MA, Bucca G, Laing E, Gubbens J, Titgemeyer F, Smith CP, Rigali S, van Wezel GP. 2015. Genome-wide analysis of in vivo binding of the master regulator DasR in Streptomyces coelicolor identifies novel non-canonical targets. PLoS One 10:e0122479. doi:10.1371/journal.pone.0122479 25875084 PMC4398421

[64] Takano E, Nihira T, Hara Y, Jones JJ, Gershater CJ, Yamada Y, Bibb M. 2000. Purification and structural determination of SCB1, a gamma-butyrolactone that elicits antibiotic production in Streptomyces coelicolor A3(2). J Biol Chem 275:11010–11016. doi:10.1074/jbc.275.15.11010 10753903

