# Supplementary material for: γ-Butyrolactone-induced coelimycin synthesis inhibits AtrA-dependent actinorhodin overproduction in Streptomyces coelicolor A3(2)
Source: mBio. 2026 Apr 15;17(5):e00630-26. doi: 10.1128/mbio.00630-26 (PMC13170358; doi:10.1128/mbio.00630-26)
Supplement: Supplemental material — Supplemental text, tables, and figures. [file mbio.00630-26-s0001.pdf]

SUPPLEMENTAL MATERIAL

γ-butyrolactone-induced coelimycin synthesis inhibits AtrA-dependent actinorhodin overproduction in *Streptomyces coelicolor* A3(2)

Bartosz Bednarz, Magdalena Kotowska, Mateusz Wenecki, Michał Tracz, Marta Derkacz, Adrianna Jastrzemska, Jarosław Ciekot, Lizaveta Karpovich, Krzysztof Pawlik

Contents

1. DETAILED MATERIALS AND METHODS DESCRIPTION 1
1 Bacterial strains and growth conditions 1
2 Label-free, bottom-up shotgun proteomics 2
3 Purification of recombinant proteins 3
4 HPLC-MS analysis of γ-butyrolactones 4
5 RNA isolation, reverse transcription and quantitative PCR 4
6 Electrophoretic mobility shift assay (EMSA) and actinorhodin extraction for EMSA 5
7 Metabolite LC-MS/MS 6
2. SUPPLEMENTAL TABLES S1-S5 8
3. SUPPLEMENTAL FIGURES 15
4. SUPPLEMENTAL MATERIAL REFERENCES 29

1. DETAILED MATERIALS AND METHODS DESCRIPTION

1 Bacterial strains and growth conditions

For genetic manipulation, *Escherichia coli* and *Streptomyces coelicolor* A3(2) strains were grown in standard conditions (1, 2). For visual imaging of the phenotypes *S. coelicolor* A3(2) strains were grown on solid medium 79 without glucose (79NG) (3). 20 µl of spore suspension in water (OD_{600}=0.3) was either spotted or spread on the surface of a square (15 mm x 15 mm) and grown at 30°C. To demonstrate the activity of exogenous GBLs, 1 ml of spore suspensions (OD_{600}=0.5) of *S. coelicolor* strains were spread by tilting the plates to obtain confluent lawns. If not indicated otherwise, after 6 h of incubation at 30°C, 10 µl of extract from a 48-h-old culture of either M1154 (GBL-containing extract) or Δ*scbA* (negative control) was added to the center of the plate, incubated further and photographed. Alternatively, the extracts were spotted next to *S. coelicolor* squares, at the distance of 7 mm. For RNA isolation and actinorhodin production studies, 200 µl of spore suspension in water (OD_{600}=0.3) was spread on solid 79NG medium overlaid with perforated cellophane and grown at 30°C. Biomass scraped from half of the surface of the cellophane from each plate and frozen in liquid

nitrogen was used for RNA isolation and qPCR analysis. For liquid cultures, 50 ml of Glu-MM medium (4) in 250 ml Erlenmeyer flasks with springs was inoculated with spores to an OD_{600} of 0.1 and incubated at 30°C on an orbital shaker (220 rpm).

2 Label-free, bottom-up shotgun proteomics

For the proteomic analysis 30 µg of total protein from each sample was denatured for 10min in 65°C in the presence of 5 mM DTT. Subsequently, samples were diluted to 2M urea concentration with 25 mM tris pH 7.5, 2 M urea, 5 mM DTT and 400 ng of trypsin was added for an overnight digestion in 37°C. On the next day the solution was acidified and desalted using the STAGE tip procedure (5). Obtained peptide pellet was reconstituted in a water solution containing 3% acetonitrile (ACN) and 0.1% formic acid (FA).

LC-MS was performed on an M-Class Acquity UPLC connected to a Synapt XS HDMS. Mobile phase A consisted of H_2O with 0.1% FA, while mobile phase B of ACN with 0.1% FA. For Δ*scbA*-*atrAOE* vs Δ*scbA*-Φ analysis a 5–35% B 120 min linear gradient was applied on a HSS C18 75 µm x 150 mm analytical column. For *ΔatrA* vs M145 analysis a 5–35% B 180 min linear gradient was applied on a HSS C18 75 µm x 250 mm analytical column. In each case the flow rate was 300 nL/min. and the column temperature was set to 45°C. A 5-minute sample trapping step was performed prior to sample separation on the analytical column in both cases. MS data were collected in ion mobility DIA (HDMSE) at a scan rate of 0.6 seconds in a 50–2000 m/z range and Resolution Analyser Mode (~45000 FHWM at 785.84 m/z). Source conditions were fine-tuned and kept constant within each analysis. For MS2, a collision energy ramp of 27–47V was set on the instrument's transfer cell. A (Glu1)-Fibrinopeptide B solution was acquired in-parallel in the reference function, and mass correction was applied post-acquisition.

Raw processing was performed using Progenesis QiP v4.2.7. QiP's autovalues were unchanged for the deconvolution, and the auto-optimization of low and high energy ion thresholds was chosen. Signal intensity between samples was normalized according to the "Normalise to All proteins" option (robust standard deviation of each samples ions' intensity estimate followed by a scalar multiplication of all ions based on that estimate). Deconvoluted data was searched via Ion Accounting against the *S. coelicolor* protein sequence databank (UP000001973) to which porcine trypsin and human keratin sequences were appended (UniProt entries). The search parameters were as follows; peptide mass tolerance: 7 ppm; fragment mass tolerance: 20 ppm; min. fragments/peptide: 1; min. fragments/protein: 3; min. peptides/protein: 1; max. protein mass: 1 MDa; digest reagent: trypsin; max. missed cleavages: 2; variable modification: oxidation of methionine; FDR (as set): 1% (protein-level). The estimated peptide-level (PSM) FDR was 0.46% and 0.55% for Δ*scbA*-*atrAOE* vs Δ*scbA*-Φ and *ΔatrA* vs M145

analyses, respectively. Protein grouping was enabled, and the Hi-N method with the top 3 most abundant peptides was chosen for relative protein quantification.

3 Purification of recombinant proteins

For His-tagged AtrA purification, 1 L of LB medium supplemented with kanamycin (50 µg/ml) was inoculated with 10 ml of an overnight culture of *E. coli* BL21(DE3)Star with the pET28a-atrA plasmid and shaken (180 rpm) at 37°C until the OD_{600} reached 0.65. The expression was induced with 1 mM IPTG, and incubation was continued for 3 h. The biomass was centrifuged (5000 × g for 10 min) and frozen at -20°C. The cells were resuspended in 20 ml of lysis buffer (50 mM Tris-HCl pH 8, 300 mM NaCl, 1 mM DTT, 0.01% Tween 20, 20 mM imidazole) supplemented with cOmplete Mini EDTA-Free Protease Inhibitor Cocktail (Sigma) and lysozyme (Fluka) (1 mg/ml) and incubated at room temperature for 15 min. The cells were disrupted at 20 kPsi (one shot) in Cell Disruptor (Constant Systems Ltd.) and centrifuged (20000 × g, 30 min, 4°C). The supernatant was applied to a 1 ml HisTrap FF column (GE Healthcare) by a peristaltic pump. The column was washed with 15 column volumes (CV) of lysis buffer, and then the protein was eluted with lysis buffer supplemented with 50 mM imidazole. The AtrA-containing fractions were pooled, and glycerol was added to a final concentration of 50%. The samples were stored at -20°C.

For His-tagged SlbR purification, two 5 L flasks, each containing 1 L of LB medium with kanamycin (50 µg/ml), were inoculated with 10 ml of an overnight culture of *E. coli* BL21 (DE3)Star containing the pET28a-slbR plasmid and shaken (180 rpm) at 37°C until the OD_{600} reached 0.6. The expression was induced with 1 mM IPTG, and incubation was continued for 6 h at 30°C. Biomass was centrifuged (5000 × g for 10 min) and frozen at -20°C. The cells were resuspended in 80 ml of lysis buffer (50 mM Tris-HCl pH 8, 500 mM NaCl, 1 mM DTT, 0.01% Tween 20, 10% glycerol, 0.01% β-mercaptoethanol, 20 mM imidazole) supplemented with protease inhibitors and lysozyme (1 mg/ml) and incubated at room temperature for 30 min. The cells were disrupted at 20 kPsi (one shot) in a cell disruptor and centrifuged (20000 × g, 30 min, 4°C). One milliliter of equilibrated HIS-Select Nickel Affinity Gel (Sigma) was added to the cleared cell lysate and incubated with mild agitation for 1 h at 4°C. The resin was centrifuged (500 × *g*, 5 min, 4°C), washed three times with lysis buffer and transferred to an empty column. The protein was eluted with lysis buffer supplemented with 100 mM imidazole. SlbR-containing fractions were pooled, and glycerol was added to a final concentration of 50%. The samples were stored at -20°C.

4 HPLC-MS analysis of γ-butyrolactones

The apparatus used was a Dionex 3000 RS-HPLC instrument equipped with a DGP-3600 pump, a WPS-3000 TLS TRS autosampler, a TCC-3000 RS column compartment (Dionex Corporation, USA) and a micrOTOF-QII mass spectrometer as a detector (Bruker Daltonics, Germany). The chromatography column used was a 50 x 2.1 (i.d.)-millimeter Thermo Scientific Hypersil Gold column with 1.9-micron particles (Part No. 25002-052130, Serial No. 0110796A6). The sample injection volume was 1 µl. The flow rate was 0.2 ml/min, and the eluate was monitored by mass spectrometry. The mobile phase was 0.1% formic acid in water (solvent A) and 0.1% formic acid in acetonitrile (solvent B). The ramp was as follows: 0 min – 5% B, 0.5 min – 5% B, 20.5 min – 95% B, 22.5 min – 95% B, and 22.6 min – 5% B. The mass spectrometer was calibrated with 10 mM sodium formate, and the following settings in positive ESI mode were used. Scan range: 100-1500 m/z, end plate offset: -500 V, capillary voltage: -4000 V, nebulizer gas (N2): 1.2 bar, dry gas (N2): 9 L/min, dry temperature: 180°C. To obtain information about GBL1-GBL8 abundance in the analysed samples, positive ion mode chromatograms were extracted based on the calculated m/z values for the four ions ([M+Na]+, [M+H]+, [M-H2O+H]+, [M-2H2O+H]+) of GBLs (SCB1-SCB8), and peak areas were calculated. GBLs with identical molecular masses were distinguished on the basis of their relative retention times according to (6).

5 RNA isolation, reverse transcription and quantitative PCR

S. coelicolor A3(2) RNA was isolated using a GeneJet RNA Purification Kit (Thermo) with a modification in the biomass disruption step. On ice, in a 2 ml Eppendorf tube, the frozen biomass was suspended by pipetting in 100 µl of buffer TE with 15 mg/ml lysozyme. Then, 300 µl of lysis buffer supplemented with DTT (as described in the manual) was added. Next, approximately 200 µl of 1 mm-diameter glass beads was added, and the sample was homogenized using a BeadBug6 homogenizer (Benchmark) in one 30 s cycle at 4000 rpm. The samples were centrifuged at 12000 × g for 1 min, applied to purification columns and processed according to the manufacturer's instructions. Elution was performed in 50 µl of nuclease-free H_2O. The removal of genomic DNA was performed by subsequent on-column purification according to the manufacturer's instructions. RNA integrity was assessed by applying ~200 ng of total RNA to a 0.5× TBE agarose gel (1%). Prior to electrophoresis, the samples were mixed with the loading dye and incubated at 70°C for 5 min.

Reverse transcription was performed with a Maxima First Strand cDNA Synthesis Kit for RT-qPCR (Thermo Fisher Scientific) using random hexamers. The total volume of the reaction was 10 µl, with the amount of DNase-treated RNA ranging from 200 ng to 800 ng. Reaction conditions were

modified for a GC-rich RNA as follows: 10 min at 25°C, 20 min at 65°C, and 5 min at 85°C. After the reaction, depending on the amount of RNA used, samples with less than 270 ng of template RNA were diluted with miliQ water 12 times (120 µl final), whereas samples with more than 270 ng were diluted 20 times (200 µl final). cDNA was stored at -80°C. In qPCR, Cq values for *hrdB* transcripts in samples after reverse transcription (RT+) and in control samples lacking the enzyme in reverse transcription reaction (RT-) were compared for assessment of genomic DNA contamination. For qPCR, RNA/cDNA samples with a Cq above 32 for RT- reactions and at least 5 cycles difference between RT+ and RT- reactions were used.

qPCR was performed on a Bio-Rad CFX96 apparatus in Hard-Shell® 96-well PCR low-profile plates (Bio-Rad, cat. no: HSP9601) with Optical Microseal 'B' PCR Plate Sealing Film (Bio-Rad, cat. no: MSB1001). PowerUp SYBR Green Master Mix (Applied Biosystems) was used for the reactions. The assay was performed for 3 biological replicates with at least 3 technical replicates for each biological replicate. For reactions with a total volume of 15 µl, 4 µl of cDNA per reaction was used. The program settings were as follows: 2 min at 50°C; 2 min at 95°C; 40 cycles of 15 s at 95°C, 30 s at 60°C, and 30 s at 72°C. After each PCR, melting curve analysis was performed. The primer sets for the targeted genes were validated by creating a standard curve with 10-fold dilutions of template material. In the reactions with 0.5 µM final primer conc. and an annealing temperature of 60°C, we achieved R^2 = 0.99 and efficiencies of 97.71% for *hrdB*, 101.56% for *atrA*, 93.69% for *actII-orf4* and 91.69% for *cpkO*. The relative gene expression ratio to that of *hrdB*, which was used as an endogenous control, was calculated using the Pfaffl formula (7).

6 Electrophoretic mobility shift assay (EMSA) and actinorhodin extraction for EMSA

EMSA was performed with AtrA and its two target DNA fragments reported earlier, the *actII-orf4* gene promoter (8) and the intergenic region between *atrA* and its neighbor *SCO4119* (9). The promoter region of *cpkO* was the target sequence of ScbR and ScbR2, and the intergenic region between *SCO6294* and *SCO6295* - of HypR. For labelling, the fragments pactII-orf4 and p6294/p6295 were amplified with the primers pTZBAM700 and pTZXBA700 from plasmids pTZ-pactII-orf4 and pTZ-p6294/p6295 as respective templates. The promoter region of *cpkO* was amplified with the primers EMSAFLUXF700 and EMSAFLUXR700 from the pFLUXH-pcpkO plasmid as a template. The fragment patrA/p4119 was amplified with either FAM or IR Dye 700 labelled primers EMSAFLUXF and EMSAFLUXR from the pFLUXH-patrA plasmid as a template. The fragment psgmA amplified with pTZBAM and pTZXBA primers from pTZ-psgmA plasmid was used as a non-specific competitor. If not indicated otherwise, each EMSA sample contained 0.06 pmol of labelled DNA and variable amounts of either AtrA or HypR protein and GBL or ACT containing/control

extracts. Other buffer components and conditions were as described previously (10). Gels were visualized using a Typhoon FLA9500 (GE Healthcare).

For the ACT extracts, 100 ml of supernatants from 68 h cultures of ACT overproducing strain Δ*scbA-atrA*$_{OE}$ and ACT non-producing strain Δ*atrA* in liquid Glu-MM medium were acidified with 1 ml of 50% acetic acid and extracted with 100 ml of ethyl acetate. 75 ml of the upper phase was vacuum evaporated to dryness and dissolved in 750 µl of methanol. A_{542} was measured and ACT concentration was calculated using the molar absorption coefficient for γ-actinorhodin $\varepsilon_{542}=18600\ M^{-1}\cdot cm^{-1}$ (2).

7 Metabolite LC-MS/MS

Plates with a confluent lawn of Δ*scbA-atrA*$_{OE}$ strain were incubated at 30°C for 6 h and a sample of agar with biomass was cut out with the wider end of a sterile 1 ml pipette tip (control sample A). Two 10 µl spots of GBL-containing extract were made on the surface of the lawn and the incubation was continued up to 25 h. Samples of agar (approx. 1.5 g) were cut out from the zone influenced by GBLs, without visible blue pigmentation (sample B) and from the outer zone where ACT production was observed. The samples were extracted overnight at room temperature with ethyl acetate (10 ml/gram of agar). The solvent was evaporated under vacuum until dryness.

Samples were reconstituted in 50:50 ACN:H_2O+0.1% FA at a ratio of 0.5g of agar mass to 1ml of solution. Prior to analysis each sample was diluted 5-fold with a 0.1% FA solution to a final concentration of 10% ACN.

LC-MS was performed on an M-Class Acquity UPLC connected to a Synapt XS HDMS. Mobile phase A consisted of H_2O with 0.1% FA, while mobile phase B of ACN with 0.1% FA. 3 µL of each sample were injected and a 15 min 5–85% B linear gradient was applied on a C18 BEH 1 mm x 100 mm analytical column for sample separation at 50 µl/min. Data were collected in ESI+ using DDA mode, with MS and MS/MS scan rate of 0.1 s, both in the 100–1700 m/z range. The top 5 precursors from each MS scan were selected for MS/MS with 1 scan allowed per transition and a dynamic exclusion window of 24 s. Collision energy ramp determined specifically for each m/z (start LM, HM: 15–55 V; end LM, HM: 20–65 V) was applied on the trap cell. To minimize background related MS/MS, a precursor exclusion list was generated from precursor peaks present in blank runs performed prior to proper analysis and applied (95 precursors, exclusion criteria: ±20 ppm and ±12 s RT). Source conditions were fine-tuned. A Leucine-Enkephalin solution was acquired in-parallel as lockmass, and correction was applied in-acquisition. Three independent biological replicates were analyzed (n=3), 9 samples in total.

For qualitative analysis raw files were processed in PLGS v3.0.3 (Waters). Obtained spectra were exported as .mgf files. Queries were limited to top 20 ions using MSConvert (11) and then

matched against GNPS libraries (12). The library search parameters were as follows; Precursor Ion Mass Tolerance: 0.01Da, Fragment Ion Mass Tolerance: 0.01Da, Score Threshold: 0.5, Min. Matched Peaks: 4. The 17Da window option filtering was enabled, while the 50Da window filtering option was disabled. Analogs were not considered. The Top Hits list was analyzed for compounds related to actinorhodin biosynthesis (5 out of 100 hits). For actinorhodin biosynthesis compounds not present in GNPS libraries, we manually investigated the .mgf files to compare with MS/MS spectra available in the literature (13) (details are summarized in table S6).

For relative quantitative analysis, an MS1 precursor transition list was generated based on the qualitative workflow results and imported into Skyline (14). The MS1 level was filtered to .mzML format from each of the raw files using MSConvert. In Skyline, XIC traces were extracted from the .mzML files using a 0.01 m/z window around 0.5min of the expected RT (the actual widest RT apex window was 2.97s) according to the imported transition list. Peaks were then integrated and their areas were TIC normalized across samples.

2. SUPPLEMENTAL TABLES S1-S5

Table S1. Bacterial strains used in this work

Strain	Relevant genotype or description	Source or reference
Escherichia coli		
DH5α	F^- *endA1 glnV44 thi-1 recA1 relA1 gyrA96 deoR nupG* Φ80Δ*lacZ*ΔM15 Δ(*lacZYA-argF*)*U169*, *hsdR17*(r_K^- m_K^+), λ^-	Promega
TOP10	*F- mcrA Δ(mrr-hsdRMS-mcrBC) φ80lacZΔM15 ΔlacX74 recA1 araD139 Δ(ara-leu)7697 galU galK rpsL(StrR) endA1 nupG*	Promega
BL21(DE3)Star	F-, *ompT*, *hsdSB* (rB-, mB-), *gal*, *dcm*, *rne131* (DE3)	Novagen
BW25113/pIJ790	*lacI*$^+$*rrnB*$_{T14}$ Δ*lacZWJ16 hsdR*514 Δ*araBAD*AH33 Δ*rhaBAD*$_{LD78}$ *rph-1* Δ*(araB–D)567* Δ*(rhaD–B)568* Δ*lacZ4787*(::*rrnB-3*) *hsdR514 rph-1* pIJ790 Recombineering strain harbouring arabinose-inducible RED genes on the plasmid pIJ790.	(15)
ET12567/pUZ8002	strain for conjugal transfer of DNA from *E. coli* to *Streptomyces* (*dam dcm hsdS* Cam^R Tet^R on the bacterial chromosome; *tra* Kan^R RP4 23 on pUZ8002)	(16)
***Streptomyces coelicolor* A3(2)**		
M145	Wild type strain, *S. coelicolor* A3(2) (SCP1$^-$ SCP2$^-$)	(2)
M1154	Δ*act* Δ*red* Δ*cpk* Δ*cda rpoB*[S433L] *rpsL*[K88E]	(17)
M145-Φ (P132)	M145 with integrated empty pIJ10257 plasmid	This work
Δ*scbA* (M751)	M145 with in-frame deletion of *scbA* gene	Gift from Eriko Takano (18)
Δ*scbA*-Φ (P053)	M751 with integrated empty pIJ10257 plasmid	This work
M145-*atrA*$_{OE}$ (P330)	M145 with integrated pIJ10257-atrA$_{OE}$	This work
Δ*scbA-atrA*$_{OE}$ (P331)	M751 with integrated pIJ10257-atrA$_{OE}$	This work
Δ*scbA-actII-orf4*$_{OE}$ (P343)	M751 with integrated pIJ10257-actII-4	This work
Δ*atrA* (P332)	*atrA* gene replacement in M145 with apramycin resistance cassette *aac3(IV)* with the use of the cosmid StD72A-atrADM (19)	This work
Δ*cpkC* (P100)	Strain unable to produce coelimycin due to *cpkC* gene disruption	(20)
Δ*cpkC-atrA*$_{OE}$ (P337)	P100 with integrated pIJ10257-atrA$_{CO}$	This work
Δ*cpkC-actII-orf4*$_{OE}$ (P344)	P100 with integrated pIJ10257-actII-4	This work
Δ*cpkC*-Φ (P339)	P100 with integrated empty pIJ10257 plasmid	This work
Δ*cpkF* (P112)	Strain unable to produce yCPK due to *cpkF* gene deletion	(21)
Δ*cpkF-atrA*$_{OE}$ (P340)	P112 with integrated pIJ10257apra-atrA$_{CO}$	This work
Δ*cpkF*-Φ (P341)	P112 with integrated empty pIJ10257apra plasmid	This work
Δ*cpkO* (P193)	M145 with a deletion of *cpkO* gene	(10)

Table S2. Plasmids and cosmids used in this work

All of the PCR-generated sequences introduced into plasmids created in this work were verified by sequencing. The constructs were introduced into *S. coelicolor* A3(2) via *E. coli* ET12567/pUZ8002-mediated conjugation (2).

Name	Relevant genotype or description	Source or reference
pUC18	Standard *E. coli* vector with a multiple cloning site (MCS) for DNA cloning	Thermo Scientific
pUC18-atrA	pUC18 with *atrA (SCO4118)* gene amplified with AtrA_F and AtrA_R primers, phosphorylated with T4 PNK (ThermoScientific) and cloned into SmaI site of the vector	This work
pET28a(+)	Novagen pET system overexpression plasmid	Novagen
pET28a-atrA	pET28a(+) containing *atrA* gene excised from pUC18-atrA with NdeI and HindIII and cloned into the same sites of the vector	This work
pUC18-slbR	pUC18 with *slbR (SCO0608)* gene amplified with SlbR_F and SlbR_R primers, phosphorylated with T4 PNK (ThermoScientific) and cloned into SmaI site of the vector	This work
pET28a-slbR	pET28a(+) containing *slbR* gene excised from pUC18-slbR with BamHI and HindIII and cloned into the same sites of the vector	This work
pIJ773	Template to amplify *aac3(IV)* apramycin resistance cassette	(19)
StD72A	SuperCos1 cosmid carrying fragment of *S. coelicolor* A3(2) chromosome (bp 4516145 to 4549188)	http://strepdb.streptomyces.org.uk
StD72A-atrA$_{DM}$	StD72A cosmid, in which *atrA* (*SCO4118*) gene sequence was replaced by means of PCR-targeting (19) with an apramycin resistance gene *aac(3)IV* amplified using primers atrADMF and atrADMR1, and pIJ773 as a template	This work
pIJ10257	ΦBT1 integrating overexpression plasmid containing strong constitutive promoter *ermEp**	(22)
pIJ10257-atrA$_{OE}$	pIJ10257 vector containing *atrA* gene excised from pUC18-atrA with NdeI and HindIII and cloned into the same sites of the vector	This work
pIJ10257-actII-4	pIJ10257 vector containing *actII-orf4* gene amplified with actII-4-F and actII-4-R primers and cloned by Gibson assembly into NdeI and HindIII-digested vector	This work
pIJ10257apra	pIJ10257 plasmid in which hygromycin resistance was replaced with apramycin resistance gene	(21)
pIJ10257apra-atrA$_{OE}$	pIJ10257apra vector containing *atrA* gene excised from pUC18-atrA with NdeI and HindIII and cloned into the same sites of the vector	This work

pTZ57R/T	T-vector from InstT/A Cloning kit for direct cloning of PCR products	Thermo Scientific
pTZ57R-pactII-orf4	pTZ57R/T containing promoter fragment of *actII-orf4*	(23)
pTZ-p6294/p6295	pTZ57R/T containing fragment p6294/p6295	(24)
pTZ57R-psgmA	pTZ57R/T containing promoter region of *sgmA* (*SCO5447*) amplified with 5447FORSHIFT and 5447REVSHIFT primers	This work
pFLUXH	ΦBT1 integrating reporter plasmid with a promoterless luciferase operon *luxCDAEB* and hygromycin resistance cassette	(25)
pFLUXH-patrA	pFLUXH containing promoter fragment of *atrA* gene amplified with atrA-6 and atrA-11 primers and cloned by Gibson assembly (26) into BamHI and NdeI-digested vector	This work
pFLUXH-pcpkO	pFLUXH containing promoter fragment of *cpkO* gene	(10)
pKNT25	Plasmid from Bacterial Two-Hybrid system, p15A origin of replication, kanamycin/neomycin resistance	Euromedex
pLW0002	Plasmid constitutively expressing GFP and ScbR, ColE1/pMB1/pBR322/pUC origin of replication, chloramphenicol resistance	Gift from Elizabeth Parkinson (27)
pLW0003	GFP assay vector, derivative of pLW0002 in which constitutive promoter of *GFP* gene was replaced with the native promoter of *scbR* followed by site R	Gift from Elizabeth Parkinson (27)
pMK48-scbR	Vector scaffold amplified with vec48-F and vec48-R primers from pKNT25 as a template and *scbR* gene with promoter for constitutive expression amplified with scbR-48-F and scbR-48-R primers from pLW0003 as a template were joined by Gibson Assembly	This work
pMK48-scbR2	Vector with promoter for constitutive protein expression amplified with vec48-F and vec48pr-R primers from pMK48-scbR as a template and *scbR2* gene amplified with scbR2-48-F and scbR2-48-R primers were joined by Gibson Assembly	This work
pMK48-atrA	*atrA* gene excised with NdeI and HindIII from pUC18-atrA and cloned into respective sites of the vector obtained from pMK48-scbR2	This work
pMK48-slbR	*slbR* gene excised with NdeI and HindIII from pUC18-slbR and cloned into respective sites of the vector obtained from pMK48-scbR2	This work
pMK48	Cloning vector for constitutive expression of proteins; NdeI and HindIII digested pMK48-scbR2 was ligated with multicloning site MCS-48 obtained by annealing of MCS-48-1 and MCS-48-2 oligonucleotides	This work

pMK49-siteR	Vector carrying *GFP* gene amplified with vec47-F and vec49-R primers on pLW0002 as a template and fragment carrying site R amplified with prom-siteR-F2 and prom-siteR-R primers were joined by Gibson Assembly	This work
pMK49-siteA	Annealed oligonucleotides siteA-1 and siteA-2 were phosphorylated and ligated with EcoRI and BamHI digested pMK49 vector obtained from pMK49-siteR plasmid	This work
pMK49-AtrA-FP1	Annealed oligonucleotides AtrA-FP1-1 and AtrA-FP1-2 were phosphorylated and ligated with EcoRI and BamHI digested pMK49 vector obtained from pMK49-siteR plasmid	This work
pMK49-FP2	Annealed oligonucleotides AtrA-FP2-1 and FP2-2 were phosphorylated and ligated with EcoRI and BamHI digested pMK49 vector obtained from pMK49-siteR plasmid	This work
pMK49-FP1R	Annealed oligonucleotides FP1R-1 and FP1R-2 were phosphorylated and ligated with EcoRI and BamHI digested pMK49 vector obtained from pMK49-siteR plasmid; FP1 site cloned in the opposite orientation than in pMK49-AtrA-FP1	This work
pMK49-FP2R	Annealed oligonucleotides FP2R-1 and FP2R-2 were phosphorylated and ligated with EcoRI and BamHI digested pMK49 vector obtained from pMK49-siteR plasmid; FP2 site cloned in the opposite orientation than in pMK49-FP2	This work

Table S3. Oligonucleotides used in this work. Restriction sites are in bold. The homology arms for Gibson assembly are underlined.

Name	Sequence 5'-3'	Restriction sites	Application/amplified fragment
AtrA_F	**CATATG**CATGTTCAGGATTCTCATTGG	NdeI	Amplification of *atrA* gene for cloning in expression plasmid
AtrA_R	**AAGCTT**TCACACCGGCCGCGACCGC	HindIII	
atrADMF	TCTCCGGGGGGAGACGTCATTACCGGGGGATTGTCTATGATTCCGGGGATCCGTCGACC		Amplification of *aac3(IV)* apramycin resistance cassette for *atrA* gene replacement
atrADMR1	GAAGGAGATACGGGCCCCCGACGACGTCGCCGCGTCTCATGTAGGCTGGAGCTGCTTC		
atrA-5	ATATCGGCATCGATGCATGATCACGCAGGTCAGCGTGGG		Amplification of *atrA* promoter region (fragment patrA) for cloning into pFLUXH
atrA-11	ATATCGGCATCGATGCATGCTAGACGACCGTGATCTCGGCC		
atrAX	GCTCAACTATGTTCTTTCCCACGGTG		Verification of *atrA* deletion
atrAY	TCACTCGTCTGTGCGGACTTCACG		
SlbR_F	**GGATCC**CATATGTCCGAGAGCACGATGCAGTCCG	BamHI	Amplification of *slbR* gene
SlbR_R	**AAGCTT**TCAGCTGCCGTTCCGCGCG	HindIII	

actII-4-F	TCTAGAACAGGAGGCCCCAATGAGATT CAACTTATTGGGACGTG		Amplification of *actII-orf4* gene
actII-4-R	CATGAGAACCTAGGATCCACTACACGA GCACCTTCTCACC		
pTZBAM700	IRDye700-ATGCAGGCCTCTGCA		Amplification and IRDye700 labelling of fragments for EMSA cloned in pTZ57R/T
pTZXBA700	IRDye700-TCGGTACCTCGCGAA		
pTZBAM	ATGCAGGCCTCTGCA		Amplification of fragments cloned in pTZ57R/T
pTZXBA	TCGGTACCTCGCGAA		
EMSAFLUXF-FAM	6FAM-CCAGTACTTCGCGAAAGC		Amplification and FAM labelling of fragments for EMSA cloned in pFLUXH
EMSAFLUXR-FAM	6FAM-ATGATGAACGAGATCTTCTTCG		
EMSAFLUXF700	IRDye700-CCAGTACTTCGCGAAAGC		Amplification and IRDye700 labelling of fragments for EMSA cloned in pFLUXH
EMSAFLUXR700	IRDye700-ATGATGAACGAGATCTTCTTCG		
EMSAFLUXF	CCAGTACTTCGCGAAAGC		Amplification of fragments cloned in pFLUXH
EMSAFLUXR	ATGATGAACGAGATCTTCTTCG		
5447FORSHIFT	GAACGCCCGGTCAACGAACC		Amplification of psgmA fragment
5447REVSHIFT	ACGGCGCGAGGTGTGGGAG		
hrdBrtoutfw	CATGCGCTTCGGACTCA		qRT-PCR (*hrdB, SCO5820*)
hrdBrtoutrv	ACTCGATCTGGCGGATG		
actII-4rtfw	GACGCGGGACTGGATCTCT		qRT-PCR (*actII-orf4, SCO5085*)
actII-4rtrv	TGCGCGATATTGCTTTCG		
atrArtfw	GTGTGTAGGCGGATGTGCTC		qRT-PCR (*atrA, SCO4118*)
atrArtrv	CCATCGAAAGCCGGAG		
vec48-F	CTAAGTAATATGGTGCACTCTCAG		Amplification of vector for pMK48-scbR plasmid construction
vec48-R	ATTGCGTTGCGCTCACTG		
vec48pr-R	ATGTTCTTTCTCCTCTTT**GGATCC**	BamHI	Together with vec48-F for amplification of vector for pMK48-scbR2 plasmid construction
scbR-48-F	CAGTGAGCGCAACGCAATGGCTGCAG TTGACGGCT		Amplification of insert for pMK48-scbR plasmid construction
scbR-48-R	GTGCACCATATTACTTAG**AAGC*TT***AGT CCTTCCCGGTCGGTGC	HindIII, *stop codon*	
scbR2-48-F	TCCAAAGAGGAGAAAGAACATATGAC CAAGCAGGAGCGG		Amplification of insert for pMK48-scbR plasmid construction
scbR2-48-R	GTGCACCATATTACTTAG**AAGC*TT***AGT GCGGCGCGTCCTGC	HindIII, *stop codon*	
MCS-48-1	TATGTA**TCTAGAATTCCCGGGTACCT CGAG**A		Annealing to obtain MCS-48 containing NdeI, XbaI, EcoRI, SmaI, KpnI, XhoI and HindIII restriction sites for pMK48
MCS-48-2	AGCTT**CTCGAGGTACCCGGGAATTCT AGA**TACA		
vec47-F	CCAGAAATCATCCTTAGCGAAAGC		

vec49-R	*CCGGCAATGCGGTTTGTTCGATC***GGATCC**CTAGTAAAGAGGAGAAATAGC	BamHI, *site R*	Amplification of vector for pMK49-siteR plasmid construction
prom-siteR-F2	GCTTTCGCTAAGGATGATTTCTGGTCGCCGTTGATAATAAGC		Amplification of insert for pMK49-siteR plasmid construction
prom-siteR-R	*GAACAAACCGCATTGCCGGTTCC***GAATTC**TTTTATTCGACTATAACAAAC	EcoRI, *site R*	
siteA-1	AATTCGAAAAAAAACCGCTCAGTCTGTATCTTAACGTTCGCGCG		Annealing to obtain insert for pMK49-siteA plasmid construction
siteA-2	GATCCGCGCGAACGTTAAGATACAGACTGAGCGGTTTTTTTTCG		
AtrA-FP1-1	AATTCATCAGGAATGCCAGATTCTATTG		Annealing to obtain insert for pMK49-AtrA-FP1 plasmid construction
AtrA-FP1-2	GATCCAATAGAATCTGGCATTCCTGATG		
AtrA-FP2-1	AATTCTGCGGGATGTGTAATTCCGCTTAAATCC		Annealing to obtain insert for pMK49-FP2 plasmid construction
FP2-2	GATCGGATTTAAGCGGAATTACACATCCCGCAG		
seq_pMK49	TGAACCCAATGTCGTTAGTG		Sequencing of pMK49-siteR

Table S4. List of co-transformations of *E. coli* TOP10 for two-plasmid GFP reporter assay

Number	GFP reporter plasmid	Plasmid for protein expression
**1**	pMK49-siteR	pMK48
**2**	pMK49-siteR	pMK48-scbR
**3**	pMK49-siteR	pMK48-slbR
**4**	pMK49-siteA	pMK48
**5**	pMK49-siteA	pMK48-scbR2
**6**	pMK49-siteA	pMK48-slbR
**7**	pMK49-FP1	pMK48
**8**	pMK49-FP1	pMK48-atrA
**9**	pMK49-FP2	pMK48
**10**	pMK49-FP2	pMK48-atrA
**11**	pMK49-FP1R	pMK48
**12**	pMK49-FP1R	pMK48-atrA
**13**	pMK49-FP2R	pMK48
**14**	pMK49-FP2R	pMK48-atrA

Table S5. Structures of γ-butyrolactones (GBLs) of *Streptomyces coelicolor* A3(2). SCB compounds are listed in the order corresponding to their retention time (compare Fig. S2). Acl compounds are included according to their side chain structures. Asterisks indicate putative new compounds.

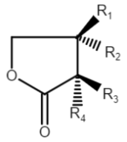

General GBL structure

Molecular formula	Name	R1	R2	R3	R4	Reference
$C_{11}H_{20}O_4$	**SCB8**	$-CH_2-OH$		$-CH(OH)-(CH_2)_4-CH_3$		(6)
$C_{12}H_{22}O_4$	**SCB4**	$-CH_2-OH$		$-CH(OH)-(CH_2)_2-CH(CH_3)-CH_2-CH_3$		(6)
	**SCB5**	$-CH_2-OH$		$-CH(OH)-(CH_2)_3-CH(CH_3)-CH_3$		(6)
	**SCB6**	$-CH_2-OH$		$-CH(OH)-(CH_2)_5-CH_3$		(6)
$C_{13}H_{24}O_4$	**SCB9***	?		?		This work
	**SCB1**	$-CH_2-OH$	$-H$	$-H$	$-CH(OH)-(CH_2)_4-CH(CH_3)-CH_3$	(28, 29)
	**Acl-2a**	$-H$	$-CH_2-OH$	$-CH(OH)-(CH_2)_4-CH(CH_3)-CH_3$	$-H$	(30)
	**Acl-2b**	$-H$	$-CH_2-OH$	$-H$	$-CH(OH)-(CH_2)_4-CH(CH_3)-CH_3$	(30)
	**Acl-2d**	$-CH_2-OH$	$-H$	$-CH(OH)-(CH_2)_4-CH(CH_3)-CH_3$	$-H$	(30)
	**SCB2**	$-CH_2-OH$	$-H$	$-H$	$-CH(OH)-(CH_2)_6-CH_3$	(29, 31)
	**Acl-2c**	$-H$	$-CH_2-OH$	$-CH(OH)-(CH_2)_3-CH(CH_3)-H_2C-CH_3$	$-H$	(30)
$C_{14}H_{26}O_4$	**SCB10***	?		?		This work
	**SCB3**	$-CH_2-OH$	$-H$	$-H$	$-CH(OH)-(CH_2)_4-CH(CH_3)-CH_2-CH_3$	(29, 31)
	**Acl-1a**	$-H$	$-CH_2-OH$	$-CH(OH)-(CH_2)_4-CH(CH_3)-CH_2-CH_3$	$-H$	(30)
	**Acl-1b**	$-H$	$-CH_2-OH$	$-H$	$-CH(OH)-(CH_2)_4-CH(CH_3)-CH_2-CH_3$	(30)
	**SCB7**	$-CH_2-OH$		$-CH(OH)-(CH_2)_7-CH_3$		(6)
	**SCB11***	?		?		This work

3. SUPPLEMENTAL FIGURES

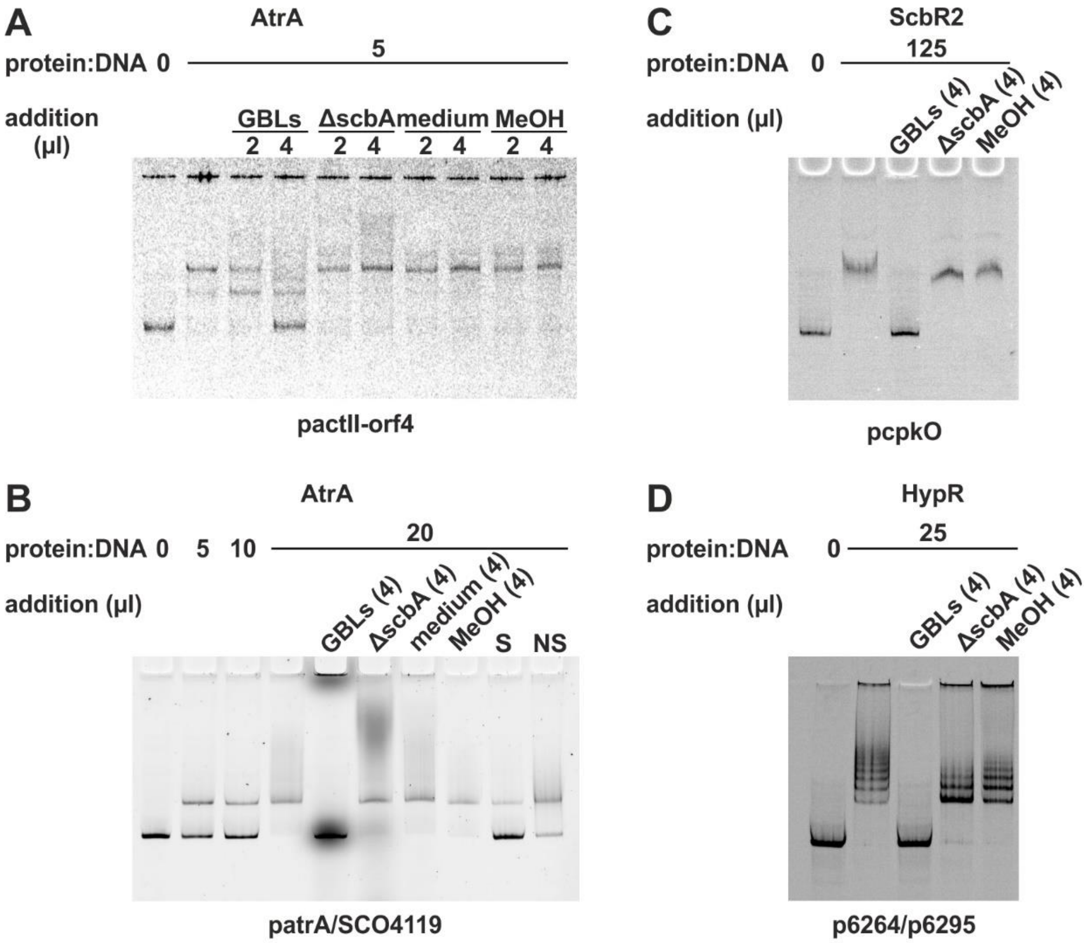

Figure S1. Electrophoretic mobility shift assay with the addition of γ-butyrolactones. (A) AtrA binding to the *actII-orf4* promoter region (0,03 pmol of probe/well), (B) AtrA binding to the *atrA/SCO4119* promoter region, (C) ScbR2 binding to the *cpkO* promoter region, (D) HypR binding to the SCO6294/SCO6295 promoter region. GBLs – extract from M1154 strain. Extracts from the *ΔscbA* strain and Glu-MM medium, as well as methanol (MeOH), were used as controls. S, NS – 10-fold excess of specific and nonspecific competitor DNA, respectively.

Figure S2. HPLC-ESI-MS extracted ion chromatograms of GBLs from *S. coelicolor* cultures. Line colours represent calculated m/z values of the four ions ($[M+Na]^+$, $[M+H]^+$, $[M-H2O+H]^+$, $[M-2H2O+H]^+$) of GBLs (SCB1-SCB8) according to Sidda et al. (6), as indicated below chromatograms. Peaks are numbered with SCB numbers. Asterisks indicate putative new compounds SCB9, SCB10 and SCB11.

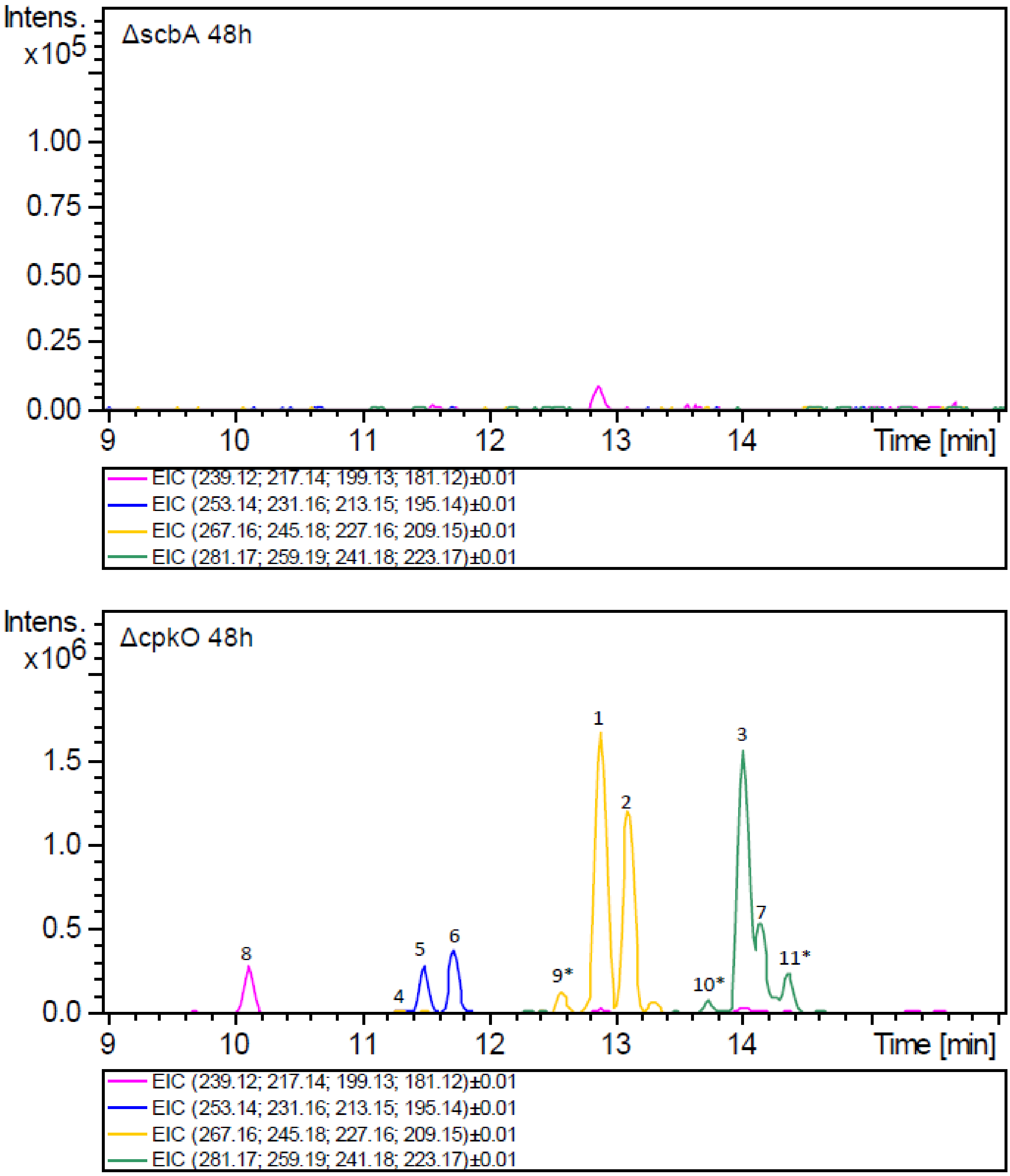

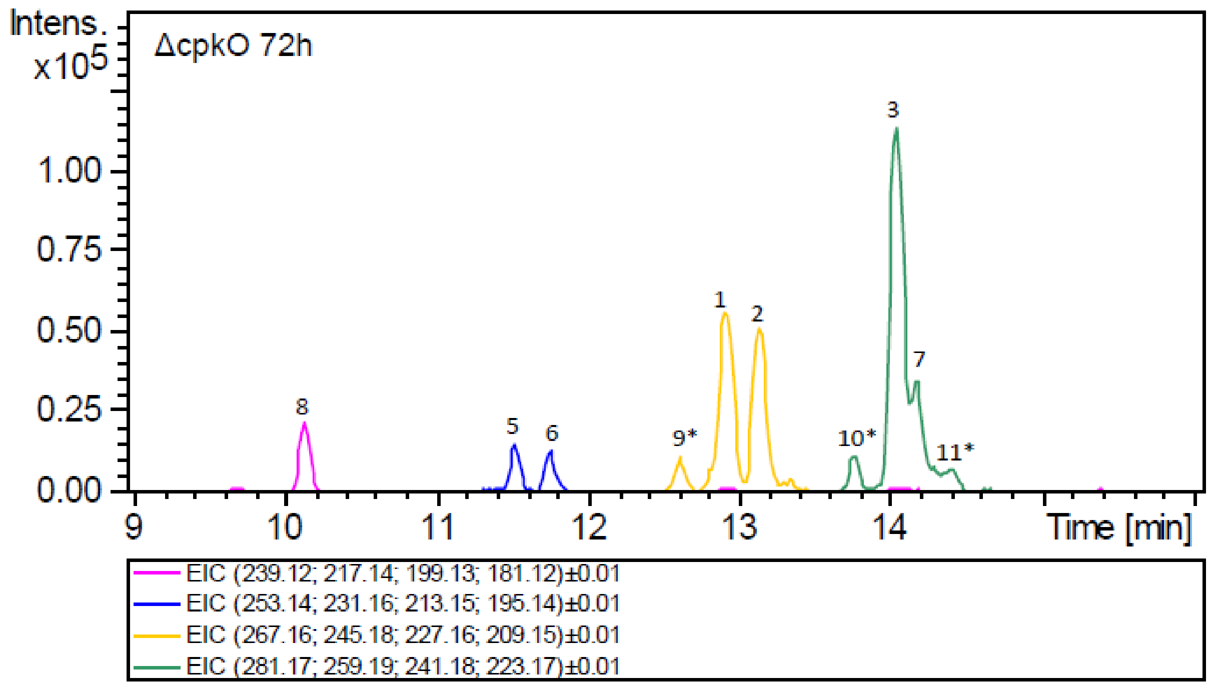
Intens.
x10^5
ΔcpkO 72h
1.00
0.75
0.50
0.25
0.00
9
10
11
12
13
14
Time [min]
8
5
6
9*
1
2
10*
3
7
11*
EIC (239.12; 217.14; 199.13; 181.12)±0.01
EIC (253.14; 231.16; 213.15; 195.14)±0.01
EIC (267.16; 245.18; 227.16; 209.15)±0.01
EIC (281.17; 259.19; 241.18; 223.17)±0.01

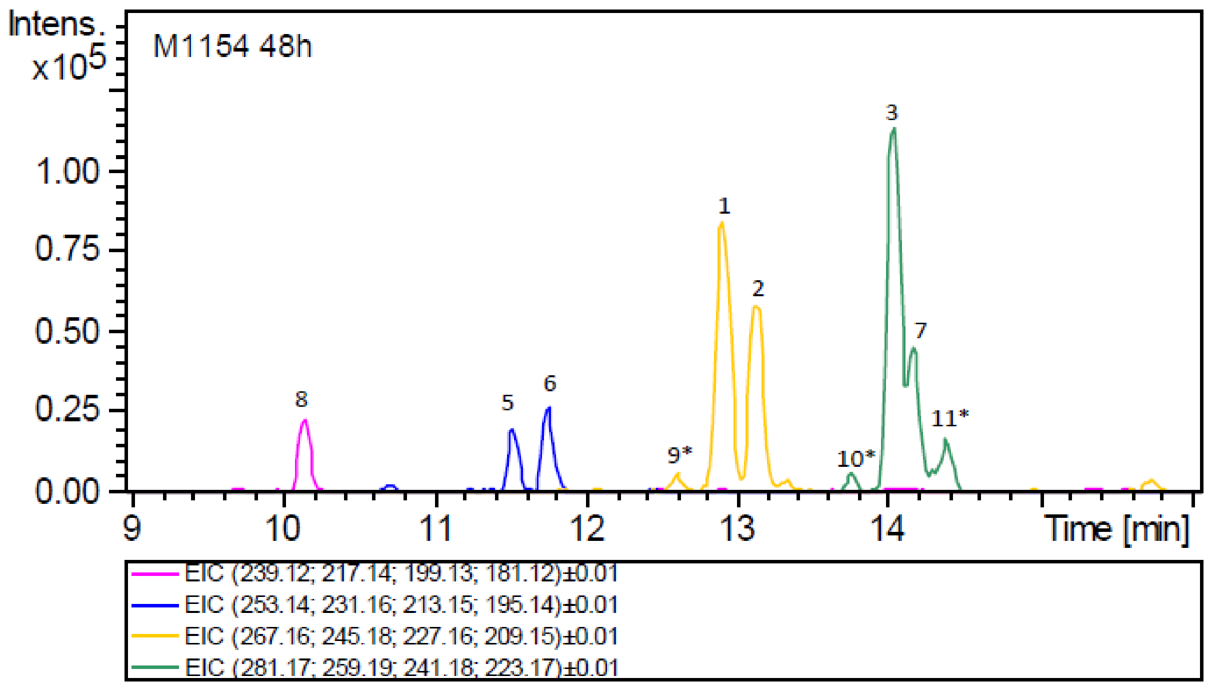
Intens.
x10^5
M1154 48h
1.00
0.75
0.50
0.25
0.00
9
10
11
12
13
14
Time [min]
8
5
6
9*
1
2
10*
3
7
11*
EIC (239.12; 217.14; 199.13; 181.12)±0.01
EIC (253.14; 231.16; 213.15; 195.14)±0.01
EIC (267.16; 245.18; 227.16; 209.15)±0.01
EIC (281.17; 259.19; 241.18; 223.17)±0.01

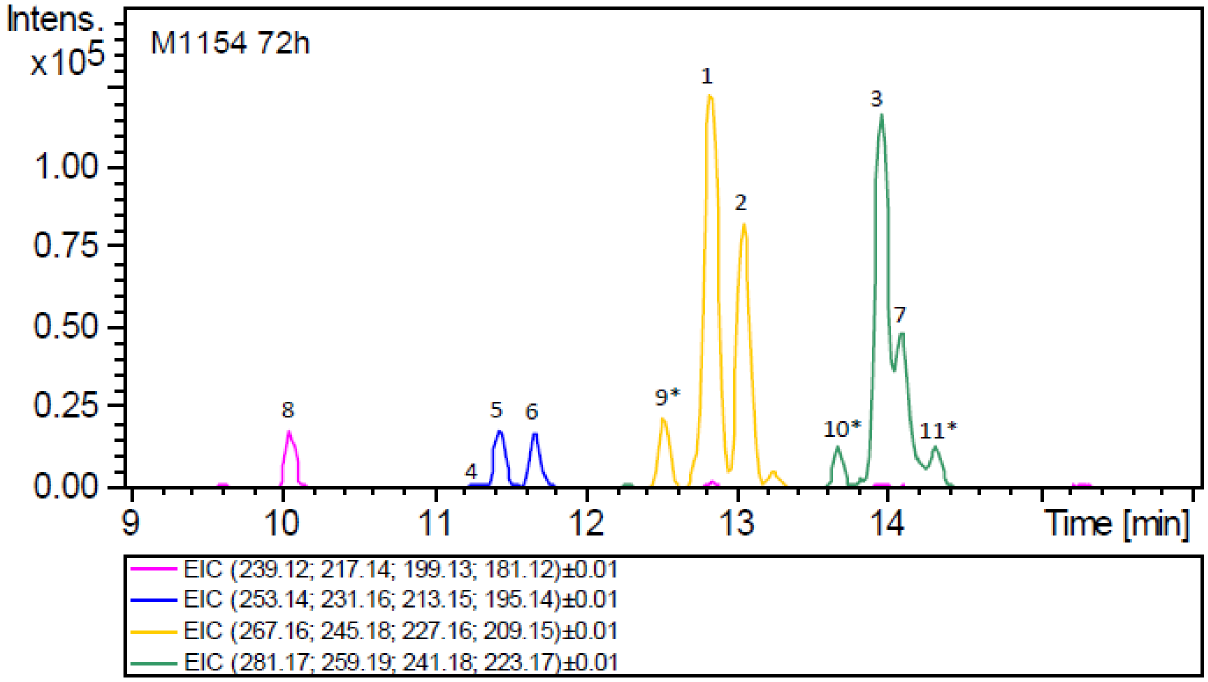

Intens.
x10^5
M1154 72h
1.00
0.75
0.50
0.25
0.00
1
2
3
7
8
4
5
6
9*
10*
11*
9
10
11
12
13
14
Time [min]
EIC (239.12; 217.14; 199.13; 181.12)±0.01
EIC (253.14; 231.16; 213.15; 195.14)±0.01
EIC (267.16; 245.18; 227.16; 209.15)±0.01
EIC (281.17; 259.19; 241.18; 223.17)±0.01

Figure S3. HPLC-ESI-MS extracted ion chromatograms of GBLs from *S. coelicolor* M1154 cultures (initial GBLs) and from samples recovered from affinity capture experiment (protein names as indicated). Line colours represent calculated m/z values of the four ions ($[M+Na]^+$, $[M+H]^+$, $[M-H2O+H]^+$, $[M-2H2O+H]^+$) of GBLs (SCB1-SCB8) according to Sidda et al. (6), as indicated below chromatograms. Peaks are numbered with SCB numbers. Asterisks indicate putative new compounds SCB9, SCB10 and SCB11.

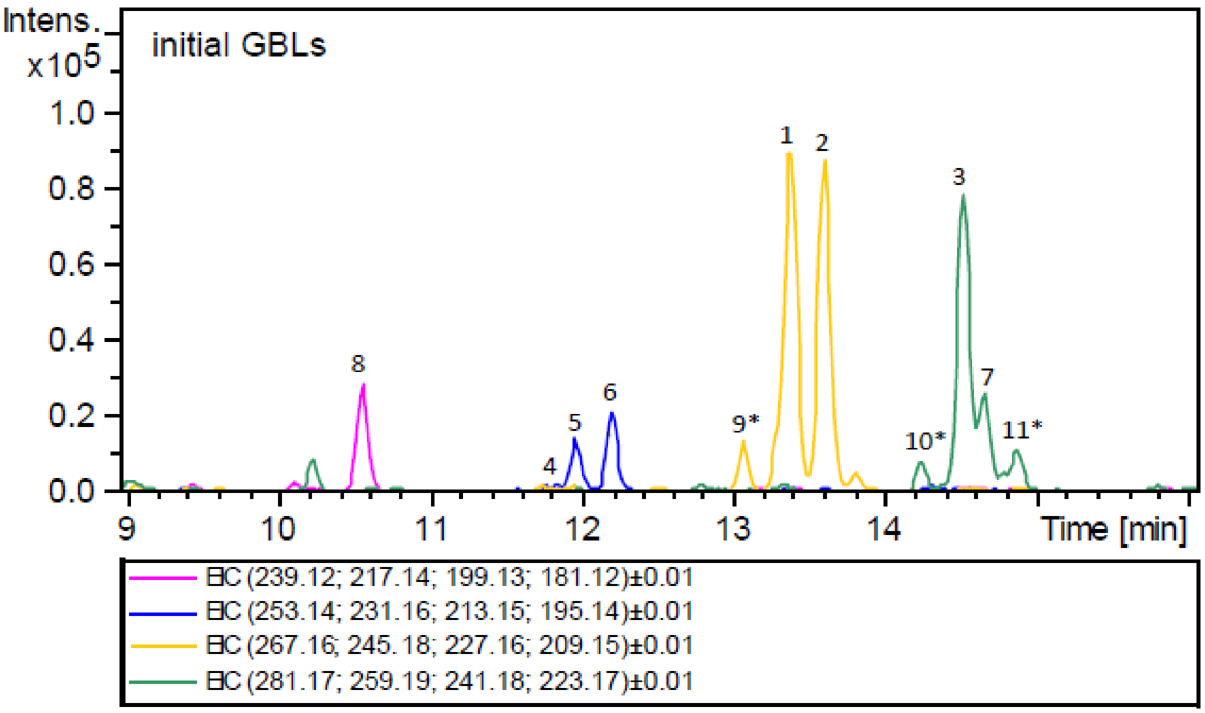

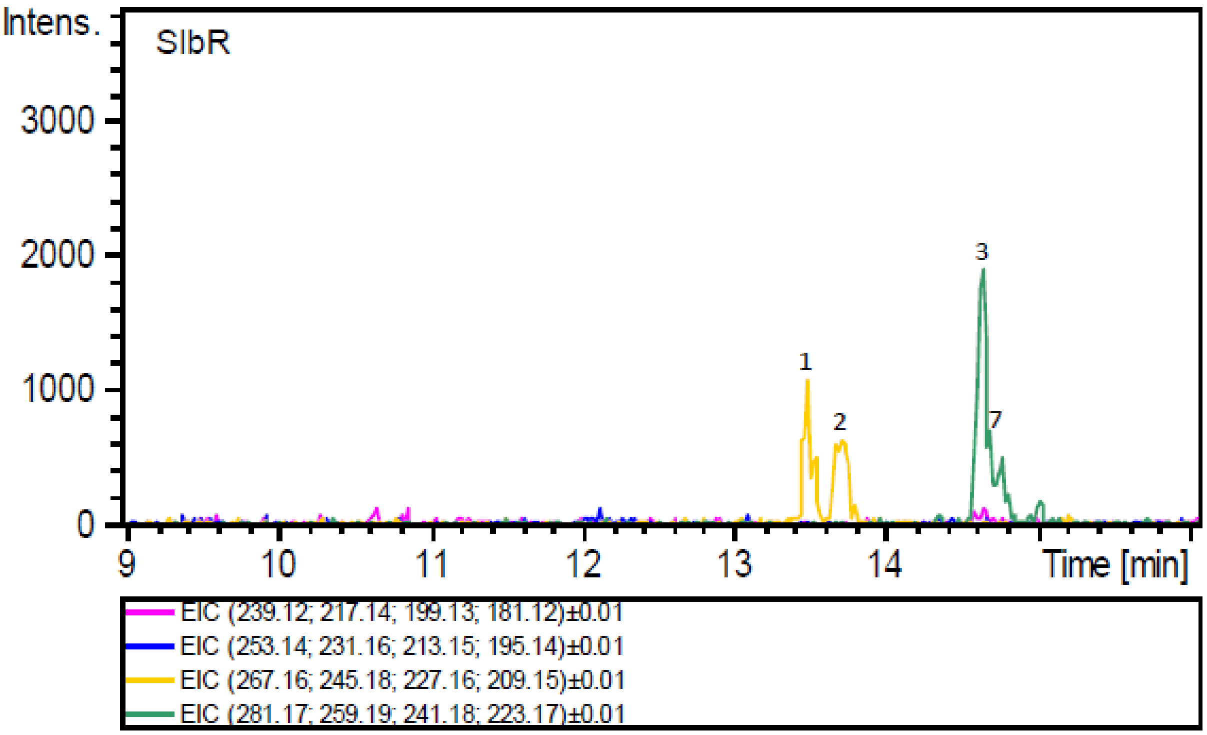

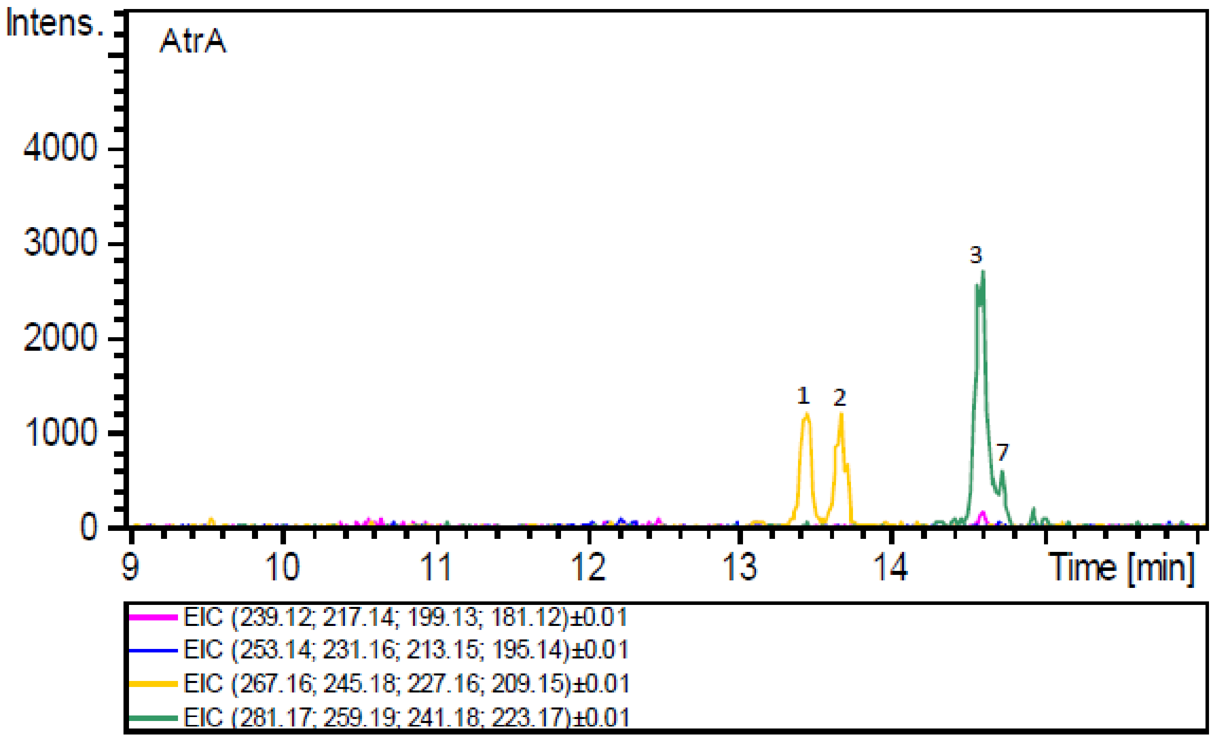

Intens.
AtrA
4000
3000
2000
1000
0
1
2
3
7
9
10
11
12
13
14
Time [min]
EIC (239.12; 217.14; 199.13; 181.12)±0.01
EIC (253.14; 231.16; 213.15; 195.14)±0.01
EIC (267.16; 245.18; 227.16; 209.15)±0.01
EIC (281.17; 259.19; 241.18; 223.17)±0.01

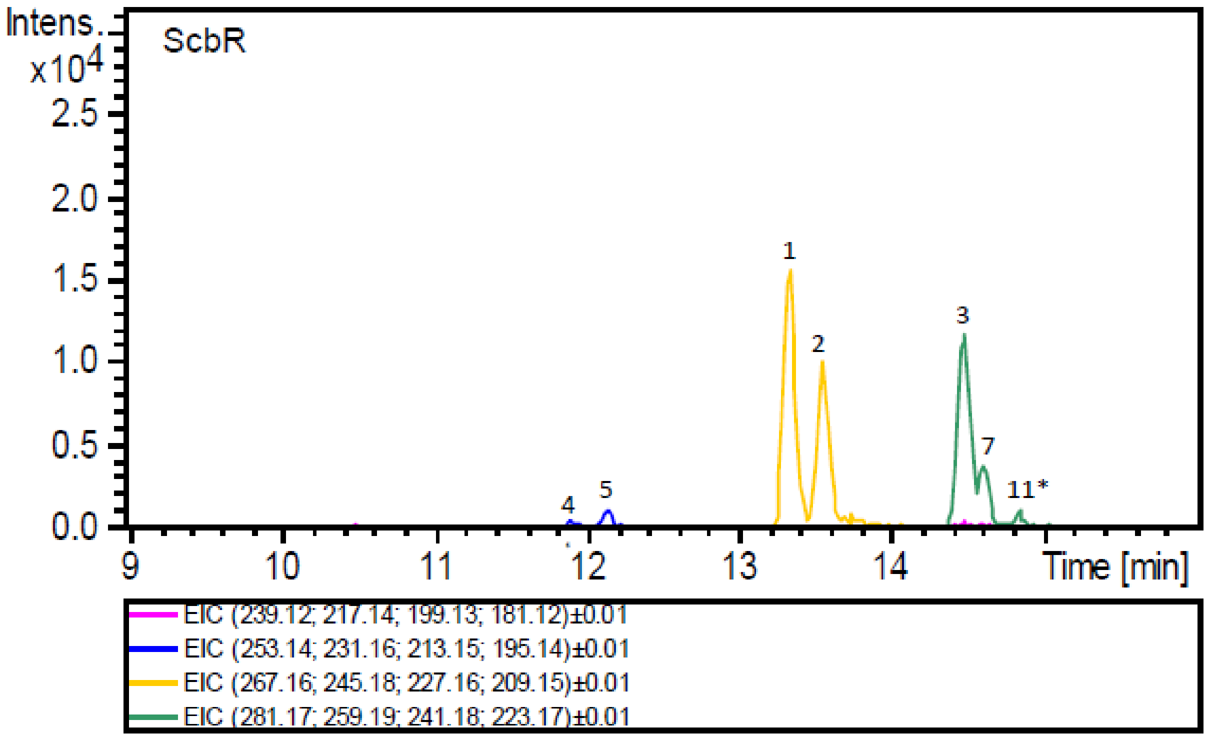

Intens.
x10^4
ScbR
2.5
2.0
1.5
1.0
0.5
0.0
1
2
3
4
5
7
11*
9
10
11
12
13
14
Time [min]
EIC (239.12; 217.14; 199.13; 181.12)±0.01
EIC (253.14; 231.16; 213.15; 195.14)±0.01
EIC (267.16; 245.18; 227.16; 209.15)±0.01
EIC (281.17; 259.19; 241.18; 223.17)±0.01

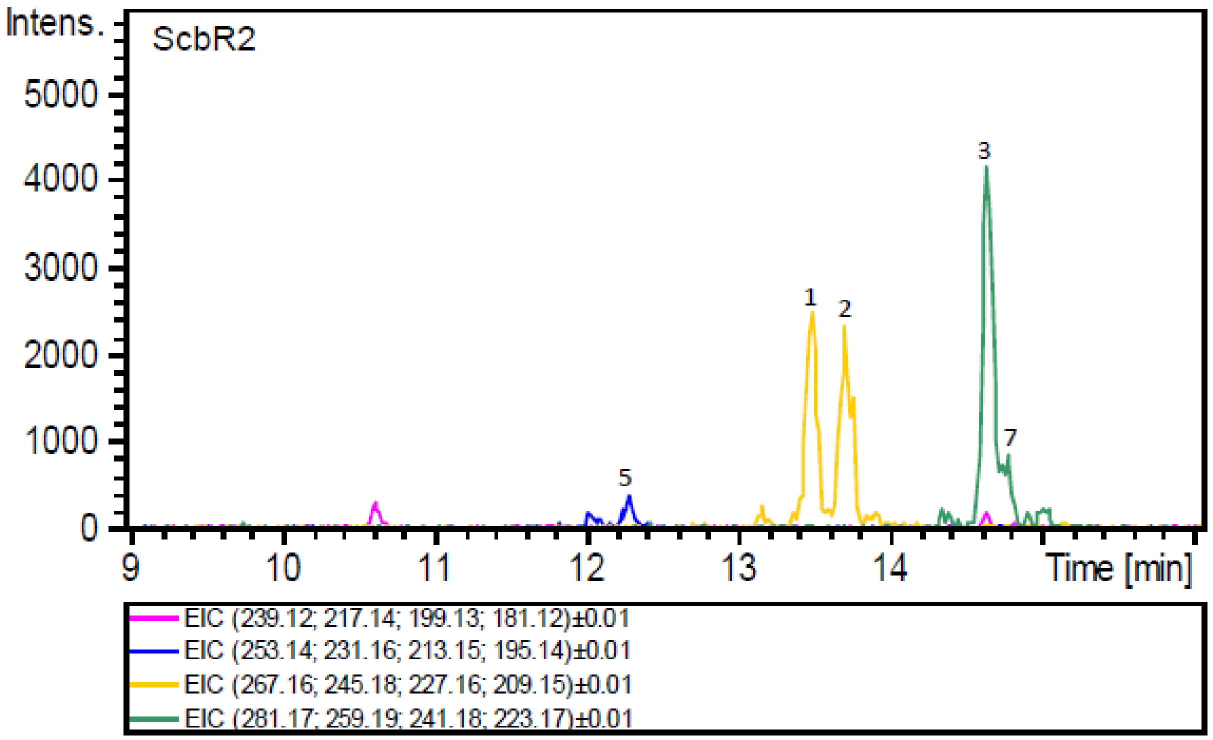
Intens.
ScbR2
5000
4000
3000
2000
1000
0
3
1
2
7
5
9
10
11
12
13
14
Time [min]
EIC (239.12; 217.14; 199.13; 181.12)±0.01
EIC (253.14; 231.16; 213.15; 195.14)±0.01
EIC (267.16; 245.18; 227.16; 209.15)±0.01
EIC (281.17; 259.19; 241.18; 223.17)±0.01

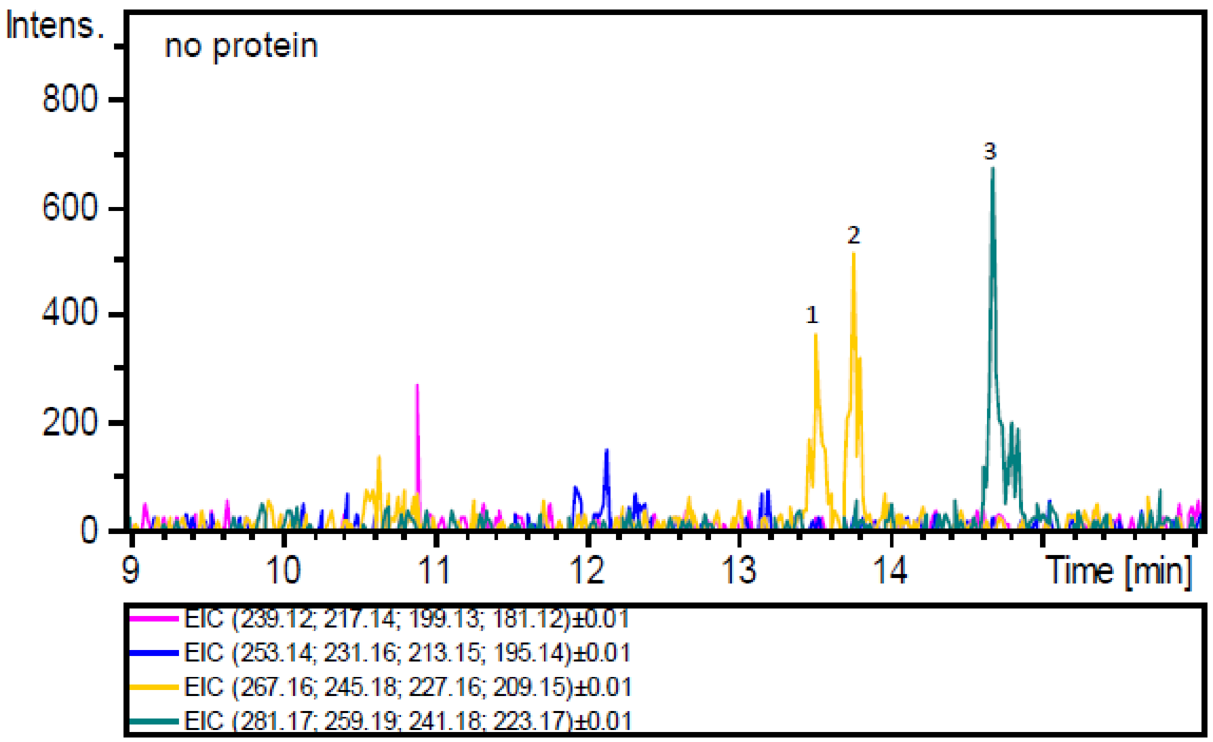
Intens.
no protein
800
600
400
200
0
3
2
1
9
10
11
12
13
14
Time [min]
EIC (239.12; 217.14; 199.13; 181.12)±0.01
EIC (253.14; 231.16; 213.15; 195.14)±0.01
EIC (267.16; 245.18; 227.16; 209.15)±0.01
EIC (281.17; 259.19; 241.18; 223.17)±0.01

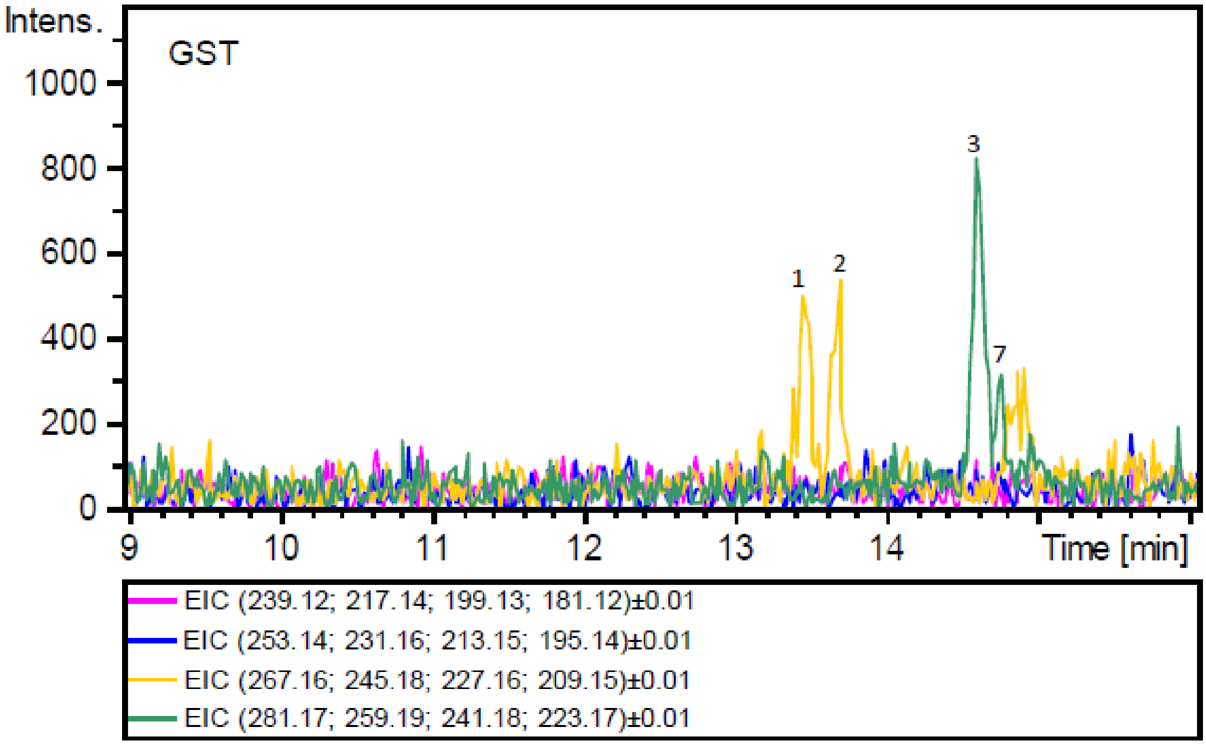
Intens.
GST
1000
800
600
400
200
0
1
2
3
7
9
10
11
12
13
14
Time [min]
EIC (239.12; 217.14; 199.13; 181.12)±0.01
EIC (253.14; 231.16; 213.15; 195.14)±0.01
EIC (267.16; 245.18; 227.16; 209.15)±0.01
EIC (281.17; 259.19; 241.18; 223.17)±0.01

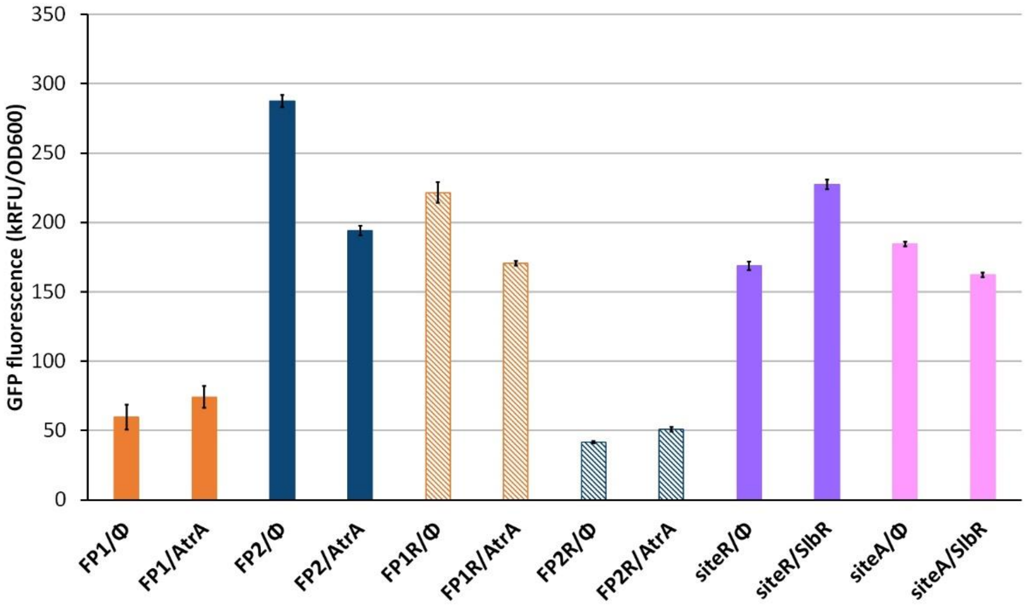

Figure S4. GFP assay results of AtrA and SlbR interactions with different binding sites. Φ – empty plasmid pMK48; FP1R, FP2R – FP1 and FP2 sites, respectively, cloned in reversed orientation into pMK49 plasmid.

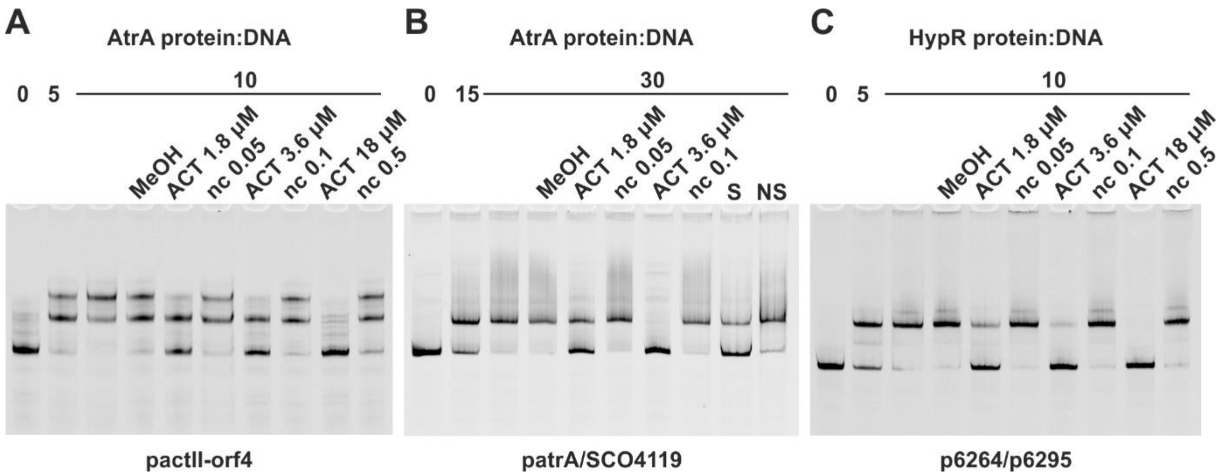

Figure S5. Electrophoretic mobility shift assay with the addition of actinorhodin. (A) AtrA binding to the *actII-orf4* promoter region, (B) AtrA binding to the *atrA/SCO4119* promoter region, (C) HypR binding to the SCO6294/SCO6295 promoter region. ACT – extract from Δ*scbA-atrA*$_{OE}$ strain, ACT concentration in the sample is shown; nc – negative control, extract from Δ*atrA* strain not producing ACT, dilution factors corresponding to the dilution of ACT containing extract in the preceding lanes are indicated; S, NS – 10-fold excess of specific and nonspecific competitor DNA, respectively.

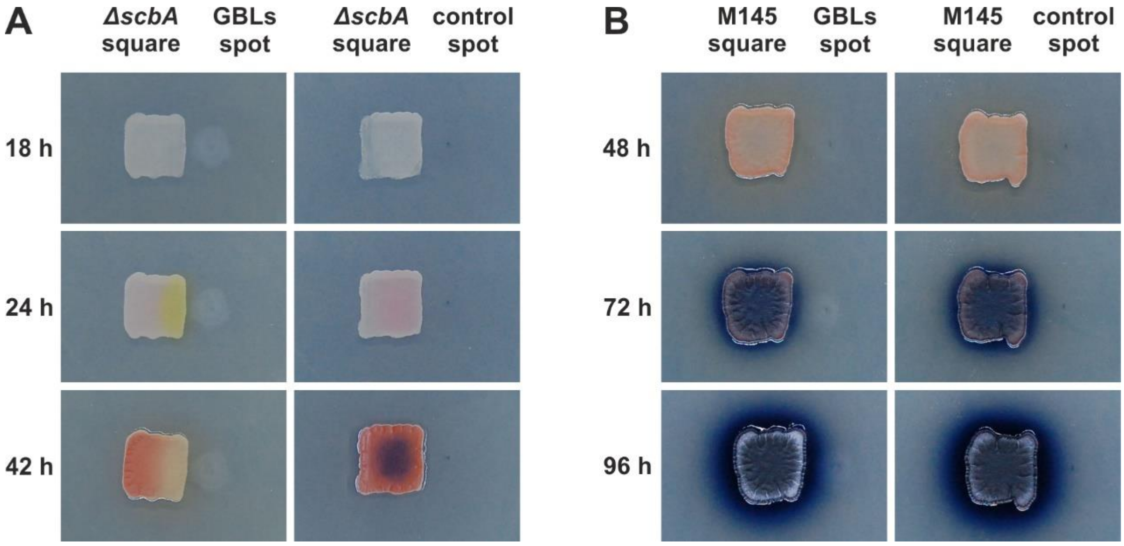

Figure S6. Effect of GBLs on *S. coelicolor* A3(2). (A) Control of GBL diffusion. GBL-rich extract spotted next to a square patch of Δ*scbA* strain grown for 18 h induced yCPK production in the neighbouring part of the square. (B) GBL-rich extract spotted next to a square patch of M145 strain grown for 48 h did not influence ACT production.

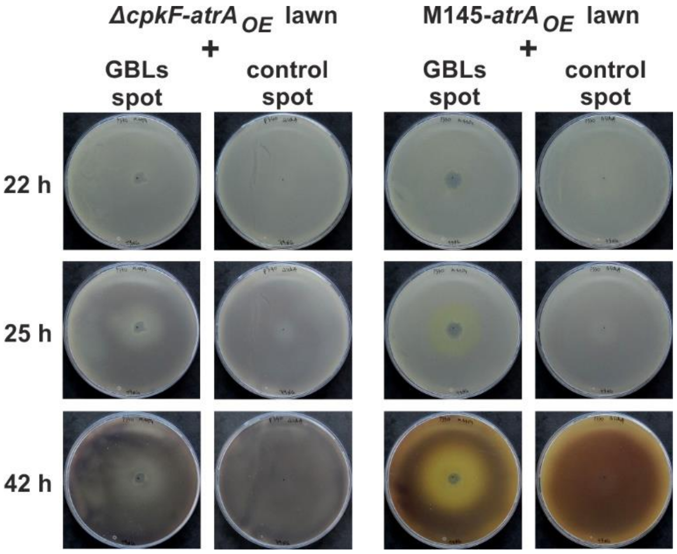

Figure S7. Effect of GBLs on *S. coelicolor* A3(2) Δ*cpkF-atrA*$_{OE}$, which is unable to produce extracellular CPK due to the lack of CpkF transporter and on M145-*atrA*$_{OE}$ strain. The negative control extract was obtained from the *ΔscbA* strain. Extracts were added after 18 h of growth.

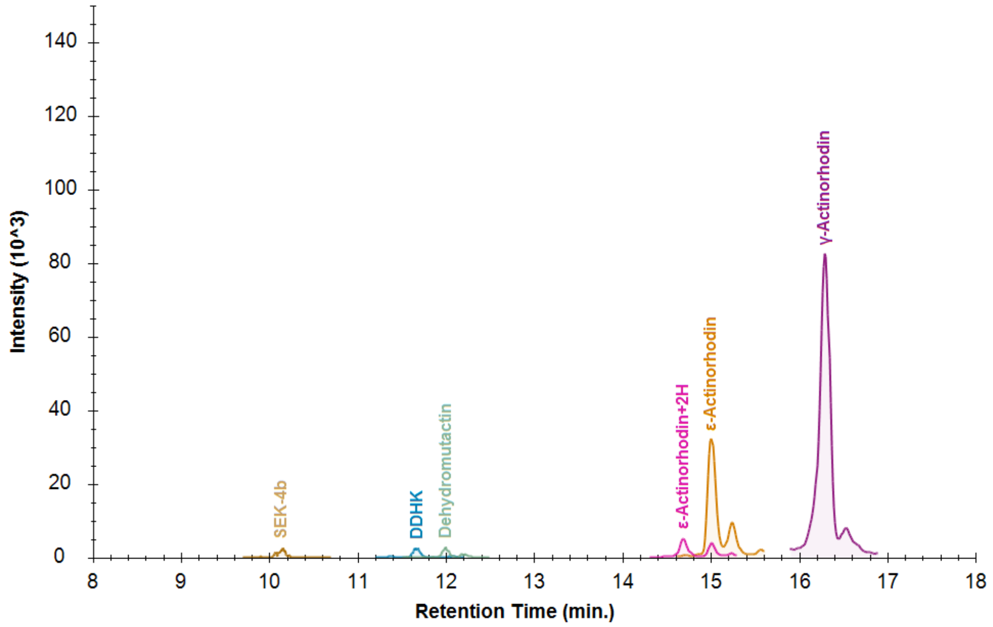

Figure S8. Representative extracted ion chromatograms of actinorhodin (two forms of ε-ACT and γ-ACT), its intermediate (6-deoxydihydrokalafungin, DDHK) and shunt products (SEK-4b and dehydromutactin) from a plate culture of Δ*scbA-atrA*$_{OE}$ (sample from zone C –without the influence of GBLs).

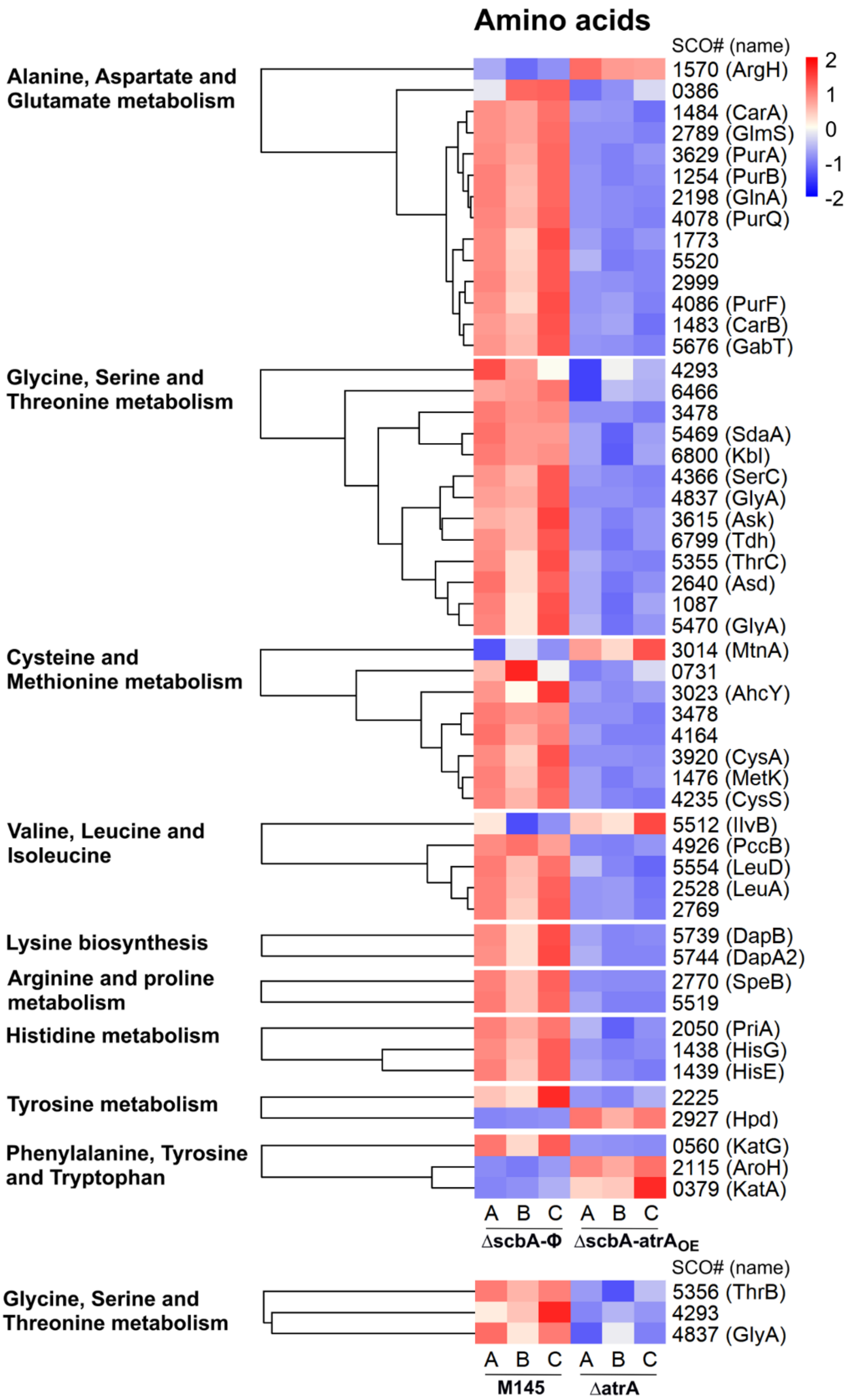

Figure S9. Statistically significant abundance changes in amino acid metabolism proteins. Where applicable, results from one or both proteomic comparisons are shown: Δ*atrA* vs M145 strain at 50 h timepoint and/or Δ*scbA*-*atrA*$_{OE}$ vs Δ*scbA*-Φ strain at 27 h timepoint. The data were row-wise z-score normalized and hierarchical clustering of rows was performed to group proteins with similar abundance patterns.

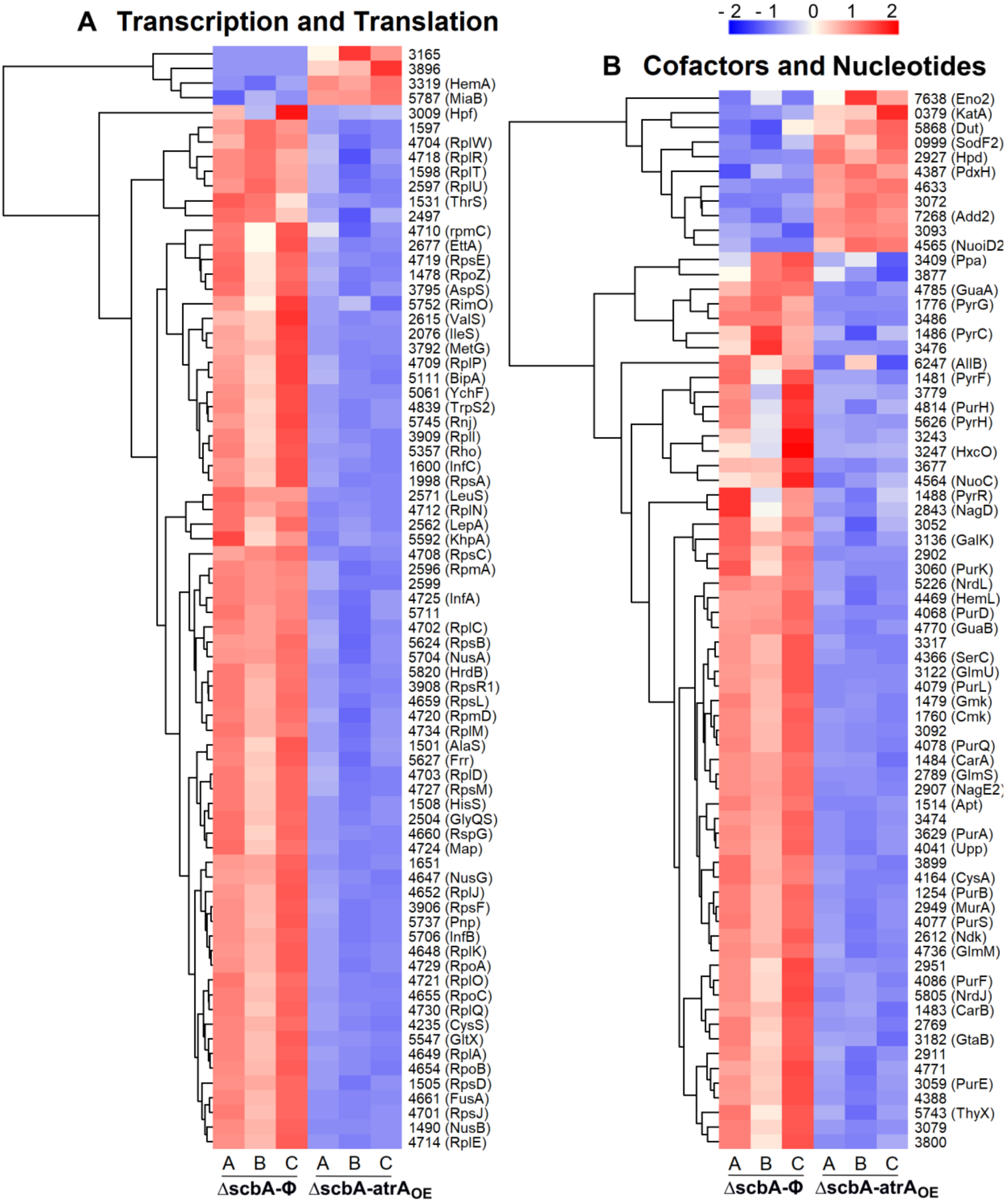

Figure S10. Statistically significant abundance changes in proteins associated with transcription and translation (A) and the synthesis of cofactors and nucleotides (B). Results are shown from the comparison Δ*scbA-atrA*$_{OE}$ vs Δ*scbA*-Φ strain at 27 h timepoint. The data were row-wise z-score normalized and hierarchical clustering of rows was performed to group proteins with similar abundance patterns.

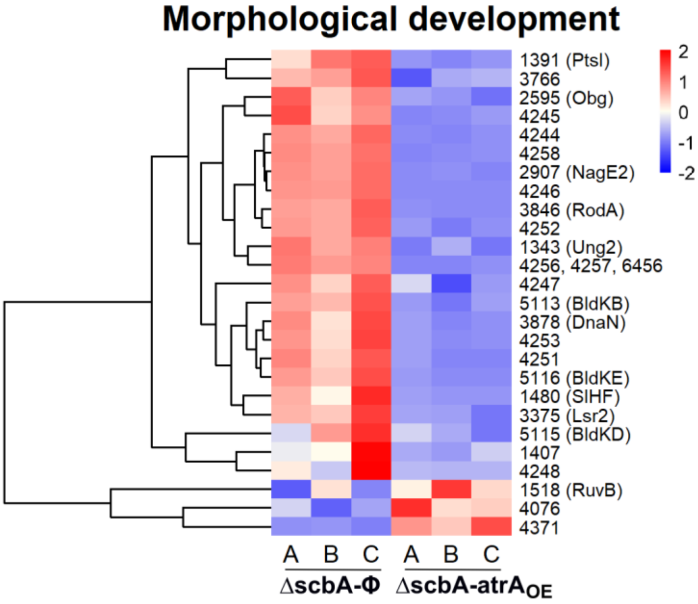

Figure S11. Statistically significant abundance changes in proteins associated with morphological development. Results are shown from the comparison Δ*scbA-atrA*$_{OE}$ vs Δ*scbA*-Φ strain at 27 h timepoint. The data were row-wise z-score normalized and hierarchical clustering of rows was performed to group proteins with similar abundance patterns.

4. SUPPLEMENTAL MATERIAL REFERENCES

1. Sambrook Joseph, Russell Davis. 2001. Molecular Cloning: A Laboratory Manual.

2. Kieser T, Bibb MJ, Buttner MJ, Chater KF, Hopwood DA. 2000. Practical Streptomyces genetics. THE JOHN INNES FOUNDATION.

3. Pawlik K, Kotowska M, Kolesiński P. 2010. Streptomyces coelicolor A3(2) Produces a New Yellow Pigment Associated with the Polyketide Synthase Cpk. Microb Physiol 19:147–151.

4. Kotowska M, Ciekot J, Pawlik K. 2014. Type II thioesterase ScoT is required for coelimycin production by the modular polyketide synthase Cpk of Streptomyces coelicolor A3(2). Acta Biochim Pol 61:141–147.

5. Rappsilber J, Ishihama Y, Mann M. 2003. Stop and go extraction tips for matrix-assisted laser desorption/ionization, nanoelectrospray, and LC/MS sample pretreatment in proteomics. Anal Chem 75:663–670.

6. Sidda JD, Poon V, Song L, Wang W, Yang K, Corre C. 2016. Overproduction and identification of butyrolactones SCB1-8 in the antibiotic production superhost: Streptomyces M1152. Org Biomol Chem 14:6390–6393.

7. Pfaffl MW. 2001. A new mathematical model for relative quantification in real-time RT-PCR. Nucleic Acids Res 29:E45.

8. Uguru GC, Stephens KE, Stead JA, Towle JE, Baumberg S, McDowall KJ. 2005. Transcriptional activation of the pathway-specific regulator of the actinorhodin biosynthetic genes in Streptomyces coelicolor. Mol Microbiol 58:131–150.

9. Ahn SK, Cuthbertson L, Nodwell JR. 2012. Genome Context as a Predictive Tool for Identifying Regulatory Targets of the TetR Family Transcriptional Regulators. PLoS One 7.

10. Bednarz B, Millan-Oropeza A, Kotowska M, Świat M, Quispe Haro JJ, Henry C, Pawlik K. 2021. Coelimycin Synthesis Activatory Proteins Are Key Regulators of Specialized Metabolism and Precursor Flux in Streptomyces coelicolor A3(2). Front Microbiol 12:787.

11. Chambers MC, MacLean B, Burke R, Amodei D, Ruderman DL, Neumann S, Gatto L, Fischer B, Pratt B, Egertson J, Hoff K, Kessner D, Tasman N, Shulman N, Frewen B, Baker TA, Brusniak MY, Paulse C, Creasy D, Flashner L, Kani K, Moulding C, Seymour SL, Nuwaysir LM, Lefebvre B, Kuhlmann F, Roark J, Rainer P, Detlev S, Hemenway T, Huhmer A, Langridge J, Connolly B, Chadick T, Holly K, Eckels J, Deutsch EW, Moritz RL, Katz JE, Agus DB, MacCoss M, Tabb DL, Mallick P. 2012. A cross-platform toolkit for mass spectrometry and proteomics. Nat Biotechnol 30:918–920.

12. Wang M, Carver JJ, Phelan V V., Sanchez LM, Garg N, Peng Y, Nguyen DD, Watrous J, Kapono CA, Luzzatto-Knaan T, Porto C, Bouslimani A, Melnik A V., Meehan MJ, Liu WT, Crüsemann M, Boudreau PD, Esquenazi E, Sandoval-Calderón M, Kersten RD, Pace LA, Quinn RA, Duncan KR, Hsu CC, Floros DJ, Gavilan RG, Kleigrewe K, Northen T, Dutton RJ, Parrot D, Carlson EE, Aigle B, Michelsen CF, Jelsbak L, Sohlenkamp C, Pevzner P, Edlund A, McLean J, Piel J, Murphy BT, Gerwick L, Liaw CC, Yang YL, Humpf HU, Maansson M, Keyzers RA, Sims AC, Johnson AR, Sidebottom AM, Sedio BE, Klitgaard A, Larson CB, Boya CAP, Torres-Mendoza D, Gonzalez DJ, Silva DB, Marques LM, Demarque DP, Pociute E, O'Neill EC, Briand E, Helfrich EJN, Granatosky EA, Glukhov E, Ryffel F, Houson H, Mohimani H, Kharbush JJ, Zeng Y, Vorholt JA, Kurita KL, Charusanti P, McPhail KL, Nielsen KF, Vuong L, Elfeki M, Traxler MF, Engene N, Koyama N, Vining OB, Baric R, Silva RR, Mascuch SJ, Tomasi S, Jenkins S, Macherla V, Hoffman T, Agarwal V, Williams PG, Dai J, Neupane R, Gurr J, Rodríguez AMC, Lamsa A, Zhang C, Dorrestein K,

Duggan BM, Almaliti J, Allard PM, Phapale P, Nothias LF, Alexandrov T, Litaudon M, Wolfender JL, Kyle JE, Metz TO, Peryea T, Nguyen DT, VanLeer D, Shinn P, Jadhav A, Müller R, Waters KM, Shi W, Liu X, Zhang L, Knight R, Jensen PR, Palsson B, Pogliano K, Linington RG, Gutiérrez M, Lopes NP, Gerwick WH, Moore BS, Dorrestein PC, Bandeira N. 2016. Sharing and community curation of mass spectrometry data with Global Natural Products Social Molecular Networking. Nat Biotechnol 34:828–837.

13. Marshall AP, Carlson EE. 2023. Metabolomics Reveals a "Trimeric" γ-Actinorhodin from Streptomyces coelicolor M145. Chembiochem 24.

14. Adams KJ, Pratt B, Bose N, Dubois LG, St. John-Williams L, Perrott KM, Ky K, Kapahi P, Sharma V, Maccoss MJ, Moseley MA, Colton CA, Maclean BX, Schilling B, Thompson JW. 2020. Skyline for Small Molecules: A Unifying Software Package for Quantitative Metabolomics. J Proteome Res 19:1447–1458.

15. Datsenko KA, Wanner BL. 2000. One-step inactivation of chromosomal genes in Escherichia coli K-12 using PCR products.

16. MacNeil DJ, Gewain KM, Ruby CL, Dezeny G, Gibbons PH, MacNeil T. 1992. Analysis of Streptomyces avermitilis genes required for avermectin biosynthesis utilizing a novel integration vector. Gene 111:61–68.

17. Gomez-Escribano JP, Bibb MJ. 2011. Engineering Streptomyces coelicolor for heterologous expression of secondary metabolite gene clusters. Microb Biotechnol 4:207–215.

18. Takano E, Chakraburtty R, Nihira T, Yamada Y, Bibb MJ. 2001. A complex role for the γ-butyrolactone SCB1 in regulating antibiotic production in Streptomyces coelicolor A3(2). Mol Microbiol 41:1015–1028.

19. Gust B, Challis GL, Fowler K, Kieser T, Chater KF. 2003. PCR-targeted Streptomyces gene replacement identifies a protein domain needed for biosynthesis of the sesquiterpene soil odor geosmin. Proc Natl Acad Sci U S A 100:1541–1546.

20. Pawlik K, Kotowska M, Chater KF, Kuczek K, Takano E. 2007. A cryptic type I polyketide synthase (cpk) gene cluster in Streptomyces coelicolor A3(2). Arch Microbiol 187:87–99.

21. Kotowska M, Wenecki M, Bednarz B, Ciekot J, Pasławski W, Buhl T, Pawlik KJ. 2024. Coelimycin inside out — negative feedback regulation by its intracellular precursors. Appl Microbiol Biotechnol 108.

22. Hong HJ, Hutchings MI, Hill LM, Buttner MJ. 2005. The role of the novel fem protein VanK in vancomycin resistance in Streptomyces coelicolor. Journal of Biological Chemistry 280:13055–13061.

23. Pawlik KJ, Zelkowski M, Biernacki M, Litwinska K, Jaworski P, Kotowska M. 2021. GntR-like SCO3932 Protein Provides a Link between Actinomycete Integrative and Conjugative Elements and Secondary Metabolism. International Journal of Molecular Sciences 2021, Vol 22, Page 11867 22:11867.

24. Kotowska M, Swiat M, Zarba-Paslawska J, Jaworski P, Pawlik K. 2019. A GntR-Like Transcription Factor HypR Regulates Expression of Genes Associated With L-Hydroxyproline Utilization in Streptomyces coelicolor A3(2). Front Microbiol 10.

25. Szafran MJ, Gongerowska M, Gutkowski P, Zakrzewska-Czerwińska J, Jakimowicz D. 2016. The Coordinated Positive Regulation of Topoisomerase Genes Maintains Topological Homeostasis in Streptomyces coelicolor. J Bacteriol 198:3016.

26. Gibson DG, Smith HO, Hutchison CA, Venter JC, Merryman C. 2010. Chemical synthesis of the mouse mitochondrial genome. Nat Methods 7:901–903.

27. Wilbanks LE, Hennigan HE, Martinez-Brokaw CD, Lakkis H, Thormann S, Eggly AS, Buechel G, Parkinson EI. 2023. Synthesis of Gamma-Butyrolactone Hormones Enables Understanding of Natural Product Induction. ACS Chem Biol 18:1624–1631.

28. Takano E, Nihira T, Hara Y, Jones JJ, Gershater CJL, Yamada Y, Bibb M. 2000. Purification and structural determination of SCB1, a γ-butyrolactone that elicits antibiotic production in Streptomyces coelicolor A3(2). Journal of Biological Chemistry 275:11010–11016.

29. Takano E. 2006. γ-Butyrolactones: Streptomyces signalling molecules regulating antibiotic production and differentiation. Curr Opin Microbiol 9:287–294.

30. Gruzina VD, Gorbatyuk E V., Efremenkova O V., Filippova SN, El'-Registan GI, Dudnik Y V. 2003. A new regulatory function of A-factor: Stimulation of the germination of streptomycete spores. Microbiology (N Y) 72:682–685.

31. Hsiao NH, Nakayama S, Merlo ME, de Vries M, Bunet R, Kitani S, Nihira T, Takano E. 2009. Analysis of Two Additional Signaling Molecules in Streptomyces coelicolor and the Development of a Butyrolactone-Specific Reporter System. Chem Biol 16:951–960.
